# Charge storage mechanisms of a π–d conjugated polymer for advanced alkali-ion battery anodes†

**DOI:** 10.1039/d2sc03127b

**Published:** 2022-06-29

**Authors:** Roman R. Kapaev, Andriy Zhugayevych, Sergey V. Ryazantsev, Dmitry A. Aksyonov, Daniil Novichkov, Petr I. Matveev, Keith J. Stevenson

**Affiliations:** Center for Energy Science and Technology, Skolkovo Institute of Science and Technology Bolshoy Boulevard 30 bld. 1 Moscow 121205 Russia roman.kapaev@skoltech.ru; Polymer Theory Department, Max Planck Institute for Polymer Research Ackermannweg 10 Mainz 55128 Germany; Department of Chemistry, Lomonosov Moscow State University Leninskie Gory 1/3 Moscow 119991 Russia

## Abstract

The demand for fast-charging metal-ion batteries underlines the importance of anodes that work at high currents with no risk of dendrite formation. NiBTA, a one-dimensional Ni-based polymer derived from benzenetetramine (BTA), is a recently proposed promising material for safe fast-charging batteries. However, its charge–discharge mechanisms remained unclear and controversial. Here we solve the controversies by providing the first rigorous study using a combination of advanced theoretical and experimental techniques, including *operando* and *ex situ* X-ray diffraction, *operando* Raman spectroscopy and *ex situ* X-ray absorption near-edge spectroscopy (XANES). In safe potential ranges (0.5–2.0 V *vs.* M^+^/M, M = Li, Na or K), NiBTA offers high capacities, fast charge–discharge kinetics, high cycling stability and compatibility with various cations (Li^+^, Na^+^, K^+^). In the Na- and K-based cells, fast bulk faradaic processes are manifested for partially reduced states. Atomistic simulations explain the fast kinetics by facile rotations and displacements of the macromolecules in the crystal, opening channels for fast ion insertion. The material undergoes distinct crystal structure rearrangements in the Li-, Na- and K-based systems, which explains different electrochemical features. At the molecular level, the charge storage mechanism involves reversible two-electron reduction of the repeating units accompanied by a change of the absorption bandgap. The reversible reduction involves filling of the orbitals localized at the ligand moieties. No reduction of NiBTA beyond two electrons per repeating unit is observed at potentials down to 0 V *vs.* M^+^/M.

## Abstract 2

Charge storage mechanisms of NiBTA, a 1D π–d conjugated polymer derived from benzenetetramine, are studied in Li-, Na- and K-based batteries with a set of advanced experimental and computational methods.

## Graphical abstract

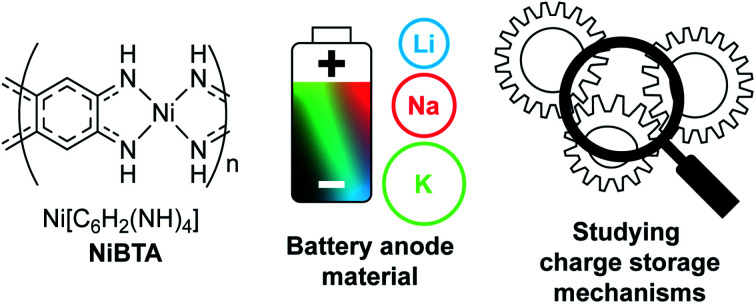

## 1. Introduction

Development of fast-charging metal-ion batteries is crucial for solving the “range anxiety” issue of electric vehicles that impedes their mass-market adoption.^1–3^ Unfortunately, conventional graphite anodes are unsuitable for fast charging because of hazardous dendrite formation at low potentials.^4–6^ High-rate capabilities can be enabled with anode materials operating in safe potential ranges (∼0.5–2.0 V *vs.* Li^+^/Li). The most popular example is Li_4_Ti_5_O_12_ (LTO), which has been employed in commercial fast-charging batteries.^7^ However, it has a limited capacity (∼175 mA h g^−1^) and a high delithiation potential (∼1.6 V *vs.* Li^+^/Li), which decreases the battery energy density.^1,7^ Research on next-generation anodes for fast-charging Li-ion batteries needs a breakthrough. It is even more desired for Na- and K-ion batteries, devices that are currently underdeveloped but are potentially more sustainable since they contain no rare lithium.^8–10^

**Fig. 1:**
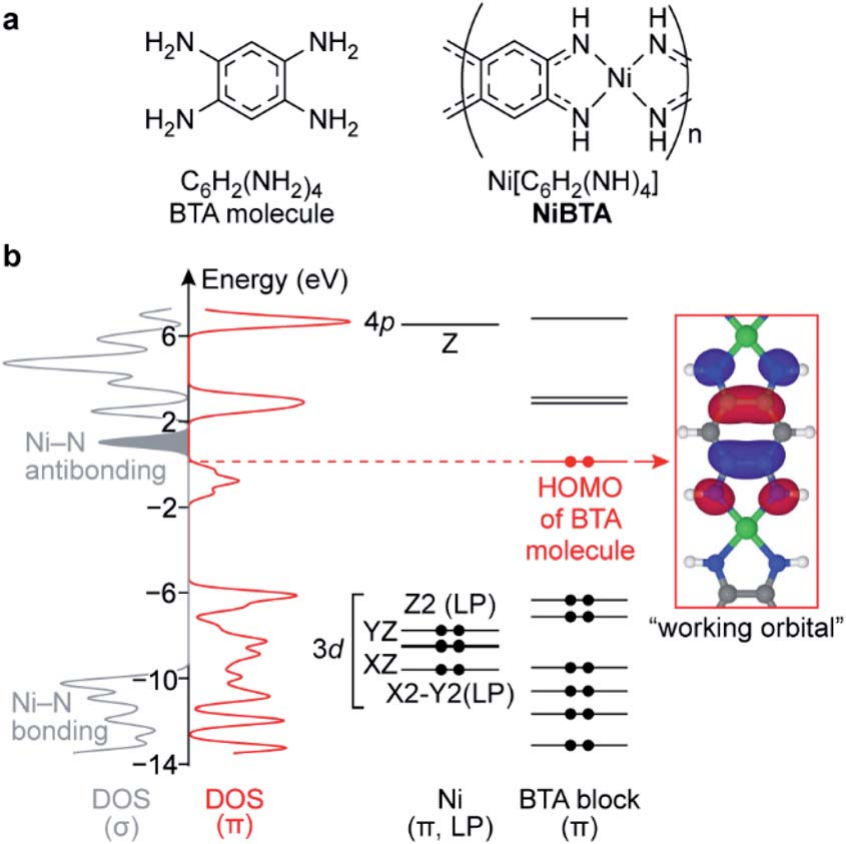
(a) Formulas of BTA molecule and NiBTA and (b) electronic structure of NiBTA. The electronic structure is partitioned into σ- and π-systems. The π-system is further decomposed into LMOs of Ni and BTA blocks. Density of Ni lone pairs (LPs) is excluded from DOS (σ). Ni LMOs are labeled by harmonic polynomials corresponding to the orbital symmetry, 3d and 4p labels are added for clarity. Electrons in BTA and Ni diagrams are shown as dots. The red dots correspond to electrons of BTA that are absent in the ligand moieties of NiBTA. 3d_*XY*_, 4s, 4p_*X*_ and 4p_*Y*_ orbitals of Ni are highly intermixed with other orbitals into σ-bands and are not shown here.

Coordination polymers with π–d conjugation^11^ have recently started to emerge as energy storage materials.^12–17^ An attractive representative is NiBTA, a Ni-based polymer derived from benzenetetramine (BTA), which is a commercially available and relatively stable ligand (Fig. 1a).^16^ However, studies of NiBTA have been scarce, unsystematic and controversial, and its charge storage mechanisms have remained unclear. Initially,^16^ two-electron reduction of the ligands was proposed for lithium-based cells in the potential range of 0.8–2.0 V *vs.* Li^+^/Li, accompanied by irreversible NH-proton substitution by Li^+^ ions. For a broader range of 0.005–3.0 V *vs.* Li^+^/Li, it was supposed that two-electron reduction of the ligands was followed by reduction of Ni^2+^ to Ni^0^, and no NH-proton substitution occurred.^14^ However, the experimental capacity (∼1200 mA h g^−1^) was higher than theoretical one for the four-electron reduction (556 mA h g^−1^). For sodium-based cells, it was suggested that the two-electron reduction of the ligands was followed by transition of Ni^2+^ to Ni^+^ in the 0.01–2.5 V *vs.* Na^+^/Na range, without further reduction to Ni^0^.^15^ For potassium-based cells, the two-electron reduction without NH-proton substitution was proposed in the 0.5–2.0 V *vs.* K^+^/K range.^17^

Suggestions about the mechanisms were made basing mainly on *ex situ* techniques, such as X-ray photoelectron spectroscopy (XPS), or indirect observations. These experimental data were ambiguous and could not give detailed information about the mechanisms. This work provides rigorous insight into the structure of NiBTA and its evolution upon charge–discharge, which was made possible by using a set of complementary experimental and theoretical methods. This is the first detailed and systematic study for π–d conjugated polymers applied in energy storage devices.

## 2. Results and discussion

### 2.1. Electronic structure

In the lowest-energy conformation, NiBTA macromolecules are planar and remain nearly planar after reduction with up to two electrons per repeating unit (Fig. S1†). The planarity allows to separate electronic σ- and π-systems. Since electronic π-couplings between the Ni and the ligand blocks are weak (Table S1†), the π-system can be further decomposed into molecular orbitals localized at Ni and at the ligands.^18,19^ These localized molecular orbitals (LMOs, Table S2†) constitute molecular orbitals (MOs) of the polymer with a minor distortion of their shapes. Band structure analysis (Fig. S3†) reveals that the wavefunction pattern weakly depends on the *k*-vector, making the LMOs easily distinguishable. The density functional theory (DFT) analysis is summarized in Fig. 1, where the σ-system is presented by its density of states (DOS), whereas the π-system is presented by its DOS and individual LMO levels.

The frontier MOs of NiBTA (Fig. S5†) are composed mainly from the LMOs of BTA (Fig. S6†). The highest occupied molecular orbital (HOMO) of BTA molecule is empty in NiBTA. It suggests redox-activity of the ligand moieties of NiBTA upon two-electron reduction. Calculations for the reduced polymer confirm this mechanism. Five occupied Ni d-orbitals (two π-conjugated, two lone pairs and Ni–N bonding MOs) lay deep in the valence band. According to population analysis (Table S5†), these orbitals remain inactive (*i.e.*, have only slight change of occupation) upon charge–discharge. Instead, by analogy with π-backbonding, Ni donates two electrons in four σ-bonds but withdraws these two electrons back from the ligand π-system.

If a formal charge at Ni was +2, its 3d_*XY*_ orbital would be empty. However, the calculations show that occupation of this deep-laying orbital is 0.8, while total occupation of Ni σ-orbitals is 1.5, much closer to two than to zero (Tables S7 and S8†). Therefore, electronic configuration of nickel is better described by a formal oxidation state of zero rather than +2, which was assigned to it previously.^14,15,17,20^ This seemingly counterintuitive zero oxidation state stresses that the redox processes for NiBTA are ligand-based, because supposed transformations of “Ni(ii)” to “Ni(i)” or “Ni(0)” lose meaning.

Above the bandgap, there are Ni–N antibonding orbitals located next to the band of the lowest unoccupied molecular orbitals (LUMO). Consequently, adding more than two electrons per repeating unit should weaken Ni–N bonds, resulting in a structural degradation or restructuring of the polymer.

For doping levels between zero and two electrons per repeating unit, the fully periodic polymer is a strongly correlated electronic system. Detailed understanding of these states requires development of a higher level of theory that is beyond the scope of this work.

### 2.2. Behavior in the potential ranges of 0.5–2.0 V *vs.* M^+^/M

#### 2.2.1. Electrochemistry

**Fig. 2:**
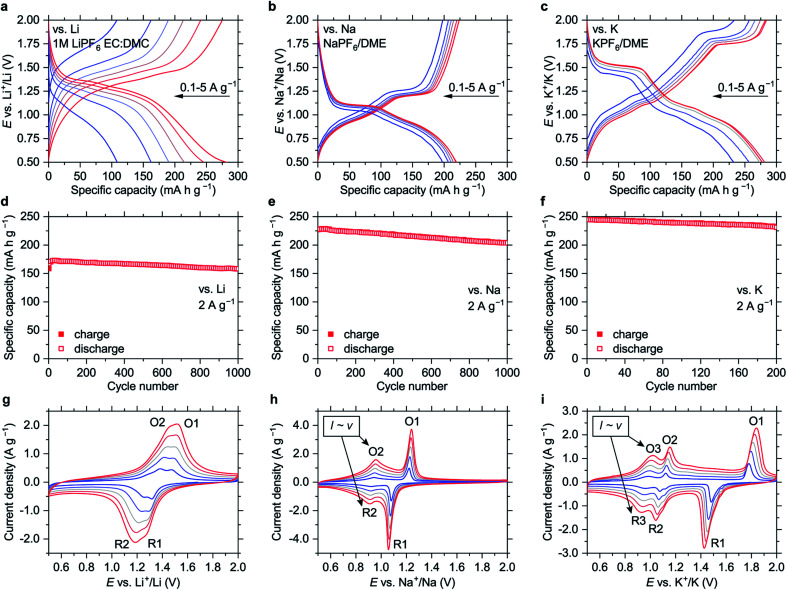
Electrochemistry of NiBTA in the 0.5–2.0 V *vs.* M^+^/M potential ranges: (a–c) charge–discharge curves at 0.1, 0.2, 0.5, 1, 2, 5 A g^−1^; (d–f) cycling performance at 2 A g^−1^; (g–i) CV profiles measured at scan rates of 0.2, 0.4, 0.6, 0.8 and 1 mV s^−1^ (higher currents correspond to higher scan rates). Peaks where the current linearly depends on the scan rate are indicated in (h) and (i). Data for the Li-, Na- and K-based cells are shown in (a, d, and g), (b, e, and h) and (c, f, and i), respectively. Prior to the CV experiments, ten scans at 1 mV s^−1^ were carried out to eliminate irreversible processes.

In the 0.5–2.0 V *vs.* M^+^/M potential ranges, the capacity per NiBTA mass reaches 280, 225 and 280 mA h g^−1^ at 0.1 A g^−1^ for Li-, Na- and K-based cells, respectively (Fig. 2a–c). This results in 265, 215 and 265 mA h g^−1^ after subtracting contributions from carbon black (Fig. S14 and S15†). Average delithiation, desodiation and depotassiation potentials are 1.35 V *vs.* Li^+^/Li, 1.1 V *vs.* Na^+^/Na and 1.35 V *vs.* K^+^/K.

The material shows high-rate capabilities at the current densities of up to 5 A g^−1^ (Fig. 2 and S18†). For lithium-based cells, the high-rate performance can be further improved *via* electrolyte optimization (Fig. S19†). The capacity fade at 2 A g^−1^ (Fig. 2d–f) is 0.005%, 0.011% and 0.028% per cycle for the Li-, Na- and K-based cells, respectively.

The polymer might be considered a promising alternative to LTO. As an anode material for Li-ion batteries that operates at safe redox potentials, it has ∼1.5 times higher specific capacity and lower average delithiation potential (1.35 V *vs.* ∼1.6 V for LTO). It also features fast charge–discharge kinetics and high cycling stability. In contrast to LTO,^21^ NiBTA is universally applicable for safe fast-charging Li-, Na- and K-ion batteries.

Cyclic voltammograms (CVs, Fig. 2g–i) reveal distinct features for different cations. CV peak profiles for the Li-based cell are typical for diffusion-controlled insertion.^22^ For the Na- and K-based systems, the features at higher potentials (O1/R1) are sharp and have a pronounced potential gap, indicating nucleation control.^22^ The features at lower potentials (O2/R2 for the Na-based cells and O3/R3 for the K-based cells) have small peak-to-peak separations (<30 mV at 0.2 mV s^−1^, Fig. S20†) and broad profiles, which are attributes of pseudocapacitive processes.^23^ Another indicator of pseudocapacitance is almost linear dependence of the peak current on the potential scan rate (Fig. S21†).^23^

If this pseudocapacitance was surface-confined, the O1/R1 features would have pseudocapacitive features or much higher integral intensities. However, the O1/R1 peaks have a signature of “battery-like” bulk processes, and the capacities of the high- and low-potential regions are comparable (Fig. 2b and c). Therefore, it appears that intercalation pseudocapacitance phenomena,^23^*i.e.*, fast bulk faradaic reactions, take place at low potentials in the Na- and K-based systems. Supposedly, initial portions of Na^+^ or K^+^ ions that insert into NiBTA act as “pillars”, expanding the structure and creating more open channels for fast diffusion of additional ions.

In the 0.5–2.0 V potential ranges, the capacities of NiBTA are close to the theoretical capacity for two-electron reduction (278 mA h g^−1^). It can be proposed that the “quinoid” forms of the ligands are reduced into “hydroquinoid” moieties, similarly to the organic quinones.^24–26^ In terms of the electronic structure, it corresponds to filling the “working orbital” of the ligands (see Section 2.1). However, this process typically gives two sets of peaks in the CV profiles, which is not the case for the K-based system (Fig. 2i).

#### 2.2.2. XRD studies and crystal structure models

**Fig. 3:**
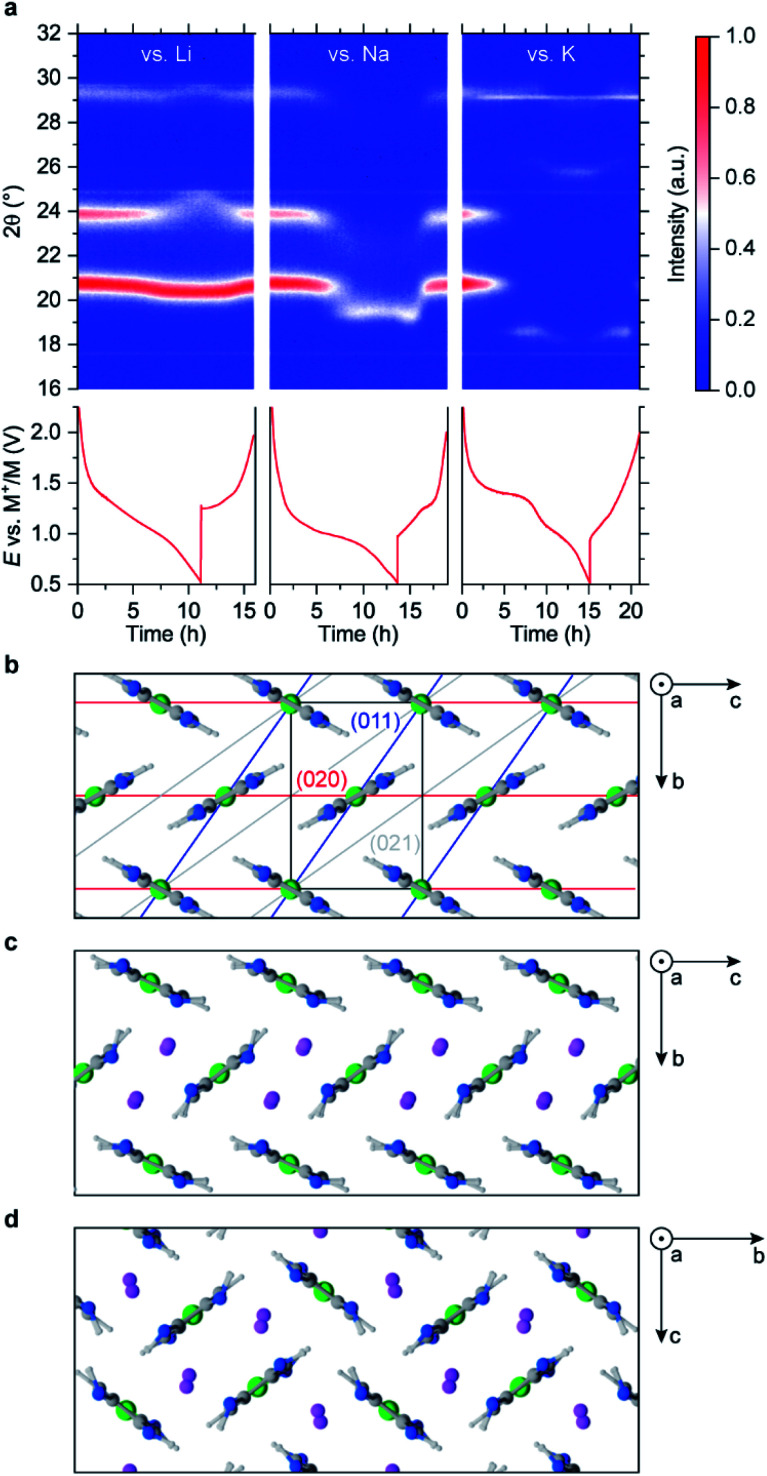
NiBTA crystal structure and its evolution: (a) *operando* XRD upon charging–discharging in Li-, Na- and K-based cells; (b) simulated crystal structure of NiBTA, shown with planes having the strongest XRD signals; (c) herringbone-type and (d) square-channeled polymorphs of fully lithiated NiBTA. Charge–discharge profiles are shown in (a) below the XRD intensity maps, time axis is common for the upper and lower plots; a narrow signal at 2θ of 29° for the K-based cell corresponds to potassium fluoride. Simulated lattices of sodiated and potassiated NiBTA are isostructural to the lithiated analogs.

Distinctions in the CV profiles can be related to different crystal structure rearrangements in the Li-, Na- and K-based cells.^27^*Operando* XRD measurements (Fig. 3a) confirm this hypothesis. The pattern of NiBTA changes only slightly upon lithiation. For the sodiation process, an obvious two-phase transition is observed when the material is roughly half-charged. For the K-based cell, two two-phase transitions are observed, which explains presence of the extra set of peaks in the CVs. Nucleation control for the Na- and K-based cells at higher potentials is likely a consequence of the major crystal structure changes that should have higher activation energy.

XRD patterns of pristine and reduced materials contain only a few broad peaks (Fig. S26–S30†), making the data ambiguous. To identify the crystal structures precisely, large crystals of NiBTA should be obtained, which is a subject of further study. To investigate the structure on the atomistic level, we performed modeling with dispersion-corrected DFT (DFT-D).

Among reasonable crystals with up to two monomers per unit cell (Fig. S23, S24, Tables S9 and S10†), the calculations show that a herringbone structure (Fig. 3b) is the lowest-energy polymorph of NiBTA, in agreement with the previous studies.^15,28^ Theoretical density of this structure is 2.16 g cm^−3^, while the experimental value for a pellet is 1.84 g cm^−3^. π-Stacking motifs (Fig. S24†), which were previously proposed for NiBTA,^20^ are energetically unfavorable. The lowest-energy π-stacked polymorph is 0.2 eV higher in energy and transforms into a herringbone structure within picoseconds in molecular dynamics (MD) simulations at room temperature.

Sliding of the macromolecules along each other in the herringbone crystal has a low activation barrier (up to 0.1 eV per monomer for the two-monomer cell, see Table S10†). Consequently, macromolecule positions along axis *a* are not fixed at room temperature. The pattern is robust only in plane *bc*, which explains why all intensive XRD peaks correspond to planes parallel to axis *a* (Table S11†). Sliding of the macromolecules broadens the XRD peaks and diminishes signals from planes that are not parallel to axis *a* (Fig. S26†).

Lithium ions are expected to insert in between (011) planes since the corresponding XRD peak (2*θ* ∼21°) remains strong but shifts to lower angles. At the same time, structural deformations that decrease the ordering of (020) planes are likely occurring, leading to weakening of the corresponding XRD peak. Several configurations were considered, including one consistent with the original crystal symmetry and another one where Li^+^ ions align with (011) planes (Table S10†). After relaxation with DFT-D, the first configuration is inconsistent with the XRD data (Fig. S28†) and substantially higher in energy than the second configuration (Table S10†). The resulting optimal herringbone structure (Fig. 3c) contains wide channels for Li^+^ ion diffusion. Alternative low-energy arrangements of the channels exist, including the square-shape tiling (Fig. 3d). There is also a π-stacked lithiated structure that is slightly lower in energy than the channeled structures (Fig. S25†). However, these structures likely do not form because of kinetic barriers required for the rearrangements of the herringbone structure. Simulated XRD pattern of the π-stacked structure poorly matches the experiment (Fig. S28†).

Larger alkaline ions deform the initial herringbone motif substantially (Fig. S25†), whereas the π-stacked configuration becomes energetically unfavorable. For Na^+^ ions, both herringbone and square-channeled polymorphs remain to have nearly the same energy (Table S10†). For K^+^ ions, the square-shape arrangement of the channels has substantially lower energy than other considered structures (Table S10†).

Qualitatively, the considered atomistic models are consistent with the observed XRD data. Upon K^+^ insertion, the main peak moves to lower angles, and the 24° peak disappears. However, the emerging peak at 26° for the K-based system is missing in these models, indicating another restructuring.

#### 2.2.3. Raman and UV-Vis-NIR spectroscopy studies

**Fig. 4:**
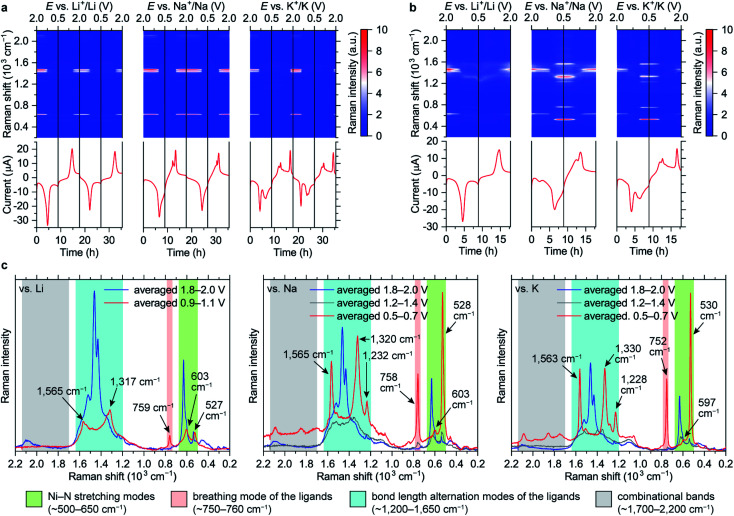
Evolution of Raman spectra of NiBTA in the 0.5–2.0 V *vs.* M^+^/M potential ranges: Raman intensity maps and current *vs.* time (*vs.* potential) profiles for the measurements with (a) the near-infrared and (b) the green lasers; (c) averaged Raman spectra for selected potential ranges during the initial discharge. The spectra in (c) were measured with the excitation wavelength of 532 nm; characteristic band regions are indicated.

From the XRD data, it is seen that the structural rearrangements of NiBTA are reversible, with signals of the initial phase reappearing upon reoxidation. *Operando* Raman spectroscopy confirms the reversibility of NiBTA reduction. With the laser excitation wavelength of 780 nm, peak intensity drops drastically upon reduction and restores after reoxidation (Fig. 4a). Spectra of the reoxidized material after the first and the second cycles are nearly identical to the initial one (Fig. S34–S36†). It shows that no irreversible structural transformations take place for NiBTA in Li-, Na- or K-ion batteries, which does not support the hypothesis about irreversible NH-proton substitution.^16^ Irreversible capacity losses originate from extrinsic factors such as SEI formation, and they can be minimized by tuning the electrolyte or electrode composition, creating artificial SEI layers, or modulating the particle size of NiBTA.^29,30^

Raman intensity of the reduced states increases with the green laser (*λ* = 532 nm, Fig. 4b and c). Spectra of the fully lithiated, sodiated and potassiated states have similar patterns, indicating that the charge storage mechanisms are similar at the molecular level. This confirms that appearance of the third set of CV peaks for the K-based cells (Fig. 2i) is caused by an additional phase transition rather than some unusual extra reduction which does not occur in the Li- and Na-based batteries. Observed variability of both Raman shifts and intensities should be associated with the differences of local environments of the macromolecules.

Since NiBTA is a narrow-gap semiconductor that absorbs near-infrared light,^20^ its resonance Raman spectra^31^ should be measured when the near-infrared laser (*λ* = 780 nm) is selected. The substantial decrease of the infrared Raman intensities upon reduction might be an indicator of the bandgap increase, which leads to deviation from the resonance condition.

To better understand the electronic structure evolution, UV-Vis-NIR spectra were acquired. While there are no issues with measuring pristine NiBTA, studying the reduced states is more challenging. Measurements of the discharged electrodes can give ambiguous results because of absorption by carbon black,^32,33^ and separating contributions from the two materials correctly is challenging. Electrochemical reduction of NiBTA without conductive fillers is problematic because of its low electronic conductivity, which for a pellet is 2.0 × 10^−7^ S cm^−1^ at room temperature (Fig. S37†). For this reason, chemical reduction of NiBTA films was carried out using excessive amount of potassium naphthalenide. Naphthalenides have low oxidation potentials of ∼0.5 V *vs.* Li^+^/Li,^34^ which makes them suitable for two-electron reduction of NiBTA. This approach has recently been shown applicable for a wide range of materials.^35–37^ Raman spectrum of NiBTA after treatment (Fig. S38†) is nearly identical to the spectrum of the fully potassiated material in the *operando* experiment, which confirms successful reduction.

**Fig. 5:**
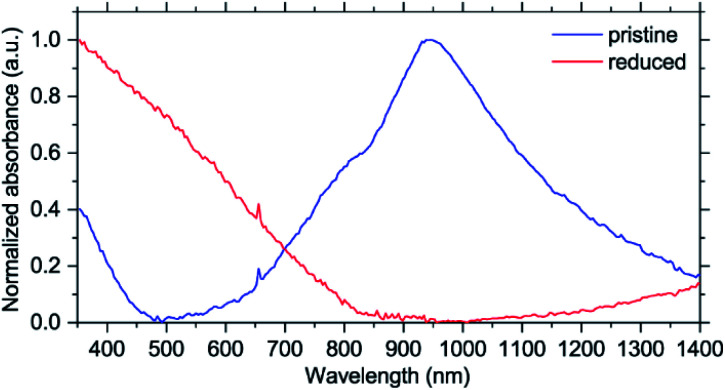
UV-Vis-NIR spectra of NiBTA in pristine and fully reduced states.

As shown in Fig. 5, pristine NiBTA has a broad absorption feature with the high-energy threshold near 600 nm and a maximum at ∼950 nm. Substantial absorption is observed at 780 nm, which should induce resonance Raman effect at this wavelength. After the reduction, this absorption band disappears, and the spectrum shows a broad absorption feature with a low-energy threshold near 800 nm and a maximum in the UV region. Observed changes of the UV-Vis-NIR spectra agree with the theoretical predictions (Fig. S39 and S40†).

#### 2.2.4. Interpretation of the UV-Vis-NIR and Raman spectra

Using oligomeric models, we provide an atomistic interpretation of the spectra using time-dependent DFT (TDDFT) for excited states and calculate off-resonance Raman activities with DFT. The observed broad absorption spectra (Fig. 5) are mainly formed by dipole-allowed electronic transitions between frontier orbitals of the bulk polymer. Absorption in the NIR range is caused presumably by a multitude of dipole–forbidden transitions and excitations originating from terminal groups that form electronic levels inside the polymer bandgap. To interpret the Raman spectra, it should be noted that the frontier MOs of NiBTA include π-conjugated MOs and the Ni–N antibonding MO. Therefore, bond-stretching modes for all non-hydrogen atoms (four Ni–N bonds and ten π-bonds in BTA) should be considered. Since NiBTA consists of loosely connected rigid blocks, modes at >200 cm^−1^ form bands that can be classified in terms of vibrations of the ligand and Ni–N stretching. Peaks at ∼1700–2200 cm^−1^ should correspond to combinational modes of the ligand and Ni–N vibrations.

The Ni–N stretching modes (∼500–650 cm^−1^ range) are well-separated in frequency from the ligand modes due to a large mass of Ni atoms. In the reduced state, the intensity of the peaks at lower frequencies increase, which is consistent with the experiment (Fig. S41†). The experimentally observed peaks have high relative intensity and are narrow, suggesting that NiN_4_ blocks are not twisted (twisted conformations should have different frequencies and lower intensity) and Ni–N bonds are strong (antibonding orbital remains empty).

Bond-stretching modes of the ligands are grouped in two bands. The lower-frequency band (∼750 cm^−1^) corresponds to a breathing mode. Since it mainly involves stretching of the two vertical C–C bonds, this mode is highly sensitive to occupation of the “working” LMO which electronic density is concentrated at these bonds (see Fig. 1). HOMO of the pristine material has zero density at the vertical C–C bonds, so the Raman signal is below detectability. For the fully reduced material, LUMO density involves these bonds, so the signal is strong. The higher-frequency band corresponds to bond-length alternation modes in a broad frequency range (∼1200–1700 cm^−1^). DFT calculations of oligomers (Fig. S41†) show multiple peaks in this range for both pristine and reduced forms, with broader distribution of frequencies for the reduced form, which is in accordance with the experiment.

#### 2.2.5. XANES studies and evoultion of charge distibutions

**Fig. 6:**
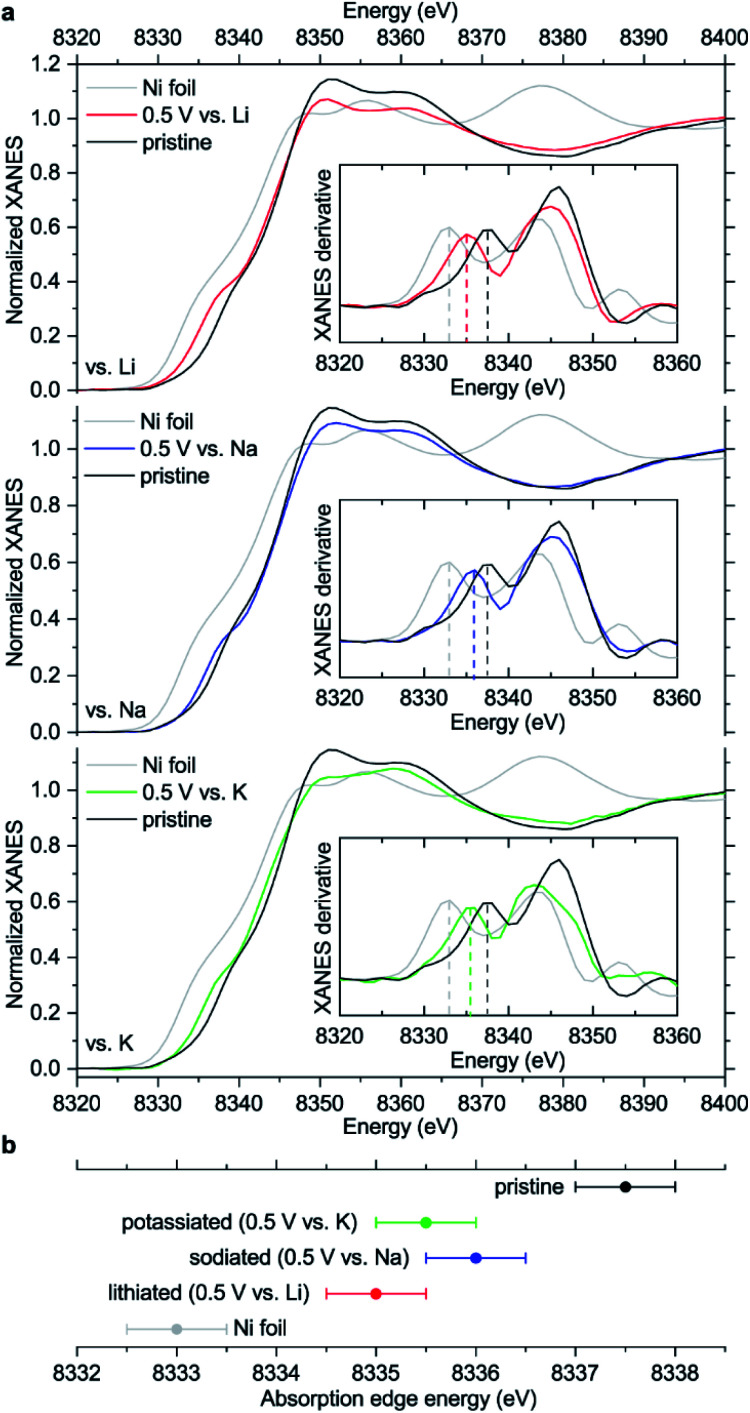
(a) XANES spectra of NiBTA-based electrodes in pristine and reduced (0.5 V *vs.* M^+^/M) states compared to Ni foil; insets: first derivatives of the XANES spectra; (b) estimated energies of XANES absorption edges. Error bars in (b) are set as halves of the energy increments during the measurement scans, which were 1 eV.

To further study the electronic structures of pristine and reduced NiBTA, we examined Ni K-edge X-ray absorption near-edge spectra (XANES), which (unlike XPS) provide reliable information about bulk materials. As shown in Fig. 6, the absorption edge energy (taken at the first inflection point) decreases by ∼2 eV after reduction of NiBTA to 0.5 V *vs.* M^+^/M. A similar shift was previously reported for NiBTA after sodiation to ∼0 V *vs.* Na^+^/Na.^15^ The spectra of reduced NiBTA cannot be represented as a linear combination of Ni and pristine NiBTA XANES, suggesting that no metallic nickel forms. The shifts to lower energies indicate an increase of electron density in the vicinity of nickel.

The observed ∼2 eV shift was previously interpreted as transition from Ni(ii) to Ni(i),^15^ basing on the literature for some inorganic compounds which proposes a linear dependence between the edge energy and oxidation state.^38–40^ However, the edge energy can vary remarkably if the ligand structure is changed, even if the formal oxidation state of a metal remains the same.^41–43^ In other words, there is no single equation for all nickel compounds linking the oxidation state with the edge energy. Moreover, the absorption edge energies for Ni^II^O and Ni^II^(OH)_2_ are shifted by ∼12–13 eV compared to metallic nickel,^44,45^ proposing a much larger ∼6 eV shift for one-electron reduction of Ni(ii) if the linear dependence is assumed.

**Fig. 7:**
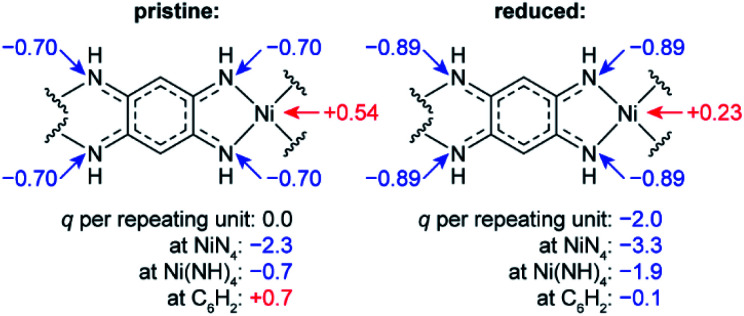
Calculated natural charges (*q*) at Ni, N atoms and selected structural units in NiBTA before and after two-electron reduction of the repeating units. See Table S6† for details.

To study how the charge distribution changes upon reduction of NiBTA, natural atomic charges were calculated for various oligomeric models (see Table S6†). These calculations show that the effective charge at Ni is +0.54 in pristine NiBTA, which decreases to +0.23 after two-electron reduction of the repeating units (Fig. 7). The same trends are revealed by analyzing Bader charges (Table S13†) and differential charge distribution maps (Fig. S42†) calculated for crystal structures. Although the absolute change for Ni is low, the relative change is nearly two-fold, which is in good agreement with the observed shifts of the absorption edges (Fig. 6b) if linear interpolation is applied.

Overall, the XANES data agree with the computations, showing that the effective charge at nickel decreases upon reduction. However, conclusion about the two-electron reduction of the ligands is still valid in terms of formal oxidation states since Ni and BTA contributions are well-separated, and the orbitals localized at Ni are only marginally involved in the redox processes (see Section 2.1).

### 2.3. Behavior in the potential ranges of 0.01–2.0 V *vs.* M^+^/M

Although operation of NiBTA in the 0.5–2.0 V *vs.* M^+^/M ranges is safer compared to deep cycling (0.01–2.0 V *vs.* M^+^/M), going below 0.5 V could be beneficial for increasing the battery energy density. Additionally, structure evolution of NiBTA at low potentials remained unclear. For these reasons, we explored the electrochemistry and structure of NiBTA during cycling in the 0.01–2.0 V *vs.* M^+^/M ranges.

As expected, broadening the potential ranges leads to an increase of the capacity per NiBTA mass unit at low currents (0.1 A g^−1^). The boost is relatively minor for the Na- and K-based cells: after subtracting the contributions from carbon, the reversible capacities stabilize at ∼300–330 mA h g^−1^ (Fig. S43†). The charge–discharge profiles remain basically the same as for cycling in the 0.5–2.0 V *vs.* M^+^/M ranges. For the Li-based cells, however, drastic changes are observed (Fig. S44†). Initial reversible capacity exceeds 1000 mA h g^−1^ at 0.1 A g^−1^. After the initial lithiation, the plateau at ∼1.2–1.5 V disappears from the discharge profiles. The capacity rapidly decays during five cycles.

**Fig. 8:**
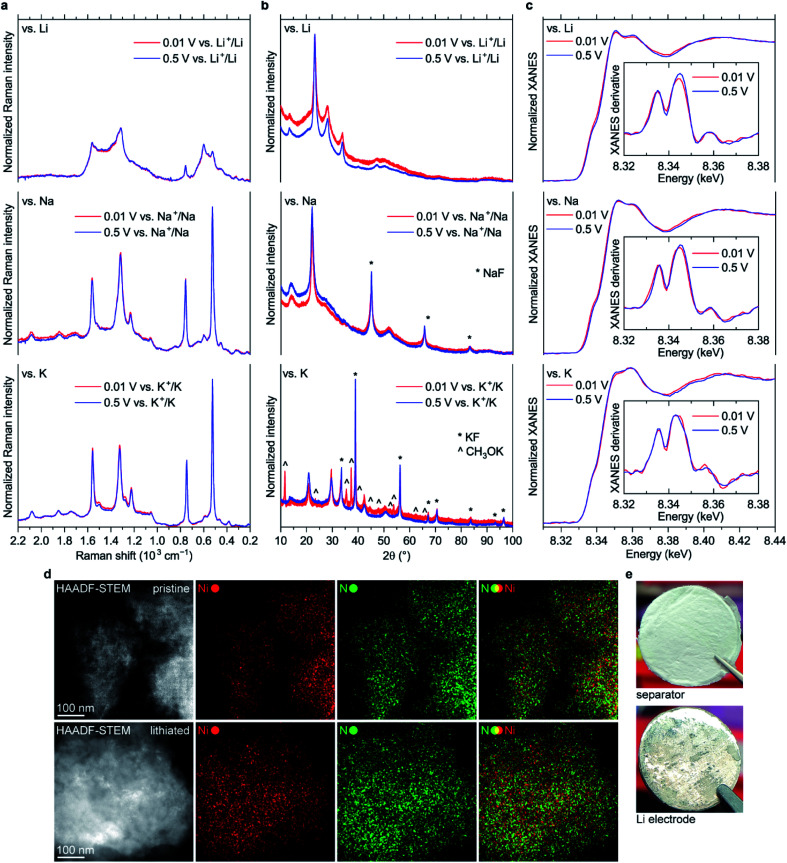
(a) Raman spectra, (b) XRD patterns and (c) XANES spectra of NiBTA-based electrodes reduced to 0.5 and 0.01 V *vs.* M^+^/M; insets in (c): first derivatives of the XANES spectra; (d) HAADF-STEM and EDX mapping images for NiBTA-based electrodes in pristine and deeply lithiated (0.01 V *vs.* Li^+^/Li) states (upper and lower images, respectively); (e) images of the separator and Li electrode from the NiBTA-based cell after five cycles in 0.01–2.0 V *vs.* Li^+^/Li range at 0.1 A g^−1^ (the electrolyte was 1 M LiPF_6_ in a 1 : 1 v/v mixture of ethylene carbonate and dimethyl carbonate). XRD peaks corresponding to sodium fluoride, potassium fluoride and potassium methoxide are indicated in (b).

Surprisingly, almost no changes of the Raman spectra are seen between 0.5 and 0.01 V *vs.* M^+^/M for all types of cells (Fig. 8a and S45†). Furthermore, there are virtually no shifts of the XRD peaks associated with NiBTA reduction products (Fig. 8b). *Ex situ* XRD patterns of the NiBTA-based electrodes after lithiation/sodiation to 0.5 V and 0.01 V are nearly identical. For the potassiated electrodes, the only major difference is presence of the peaks of potassium methoxide after deep reduction, which should be due to the electrolyte decomposition at low potentials. *Ex situ* XANES also shows that there is no major difference between the electrodes reduced to 0.5 and 0.01 V (Fig. 8c).

According to theory (see Section 2.1), NiBTA reduction beyond two electrons per repeating unit leads to Ni–N bond weakening, which should eventually result in the material decomposition. It is expected that decomposition of a coordination polymer results in detachment of the ligands from the transition metal and dissolution of organic moieties.^46^ However, no evidence of such decomposition is observed according to EDX mapping with sub-nanometer resolution, which shows that Ni and N remain in a single phase (Fig. 8d). Additionally, post-mortem analysis of the Li-based cells after deep cycling reveals no signs of the material dissolution since there is no coloring of the separator and lithium surface remains shiny (Fig. 8e).

Overall, the presented study shows strong evidence that NiBTA accepts only two electrons per repeating unit, and no further reduction occurs even at ∼0 V *vs.* M^+^/M. Therefore, the capacity higher than the theoretical one (278 mA h g^−1^) is unrelated to the intrinsic chemistry of NiBTA.

Nature of the extra capacity (especially for the Li-based cells) is unclear and is a subject of further studies. It could be supposed that this phenomenon is related to reversible electrolyte reduction catalyzed by reduced NiBTA,^47,48^ as suggested by an increased content of oxygen in the lithiated electrode compared to pristine and reoxidized states (Fig. S47†). However, it is surprising that the electrochemical output is similar for entirely different electrolyte compositions (Fig. S44†). Other reasons can include capacitive processes and charge storage in defects.^48^ However, these factors are not expected to increase the capacity dramatically, so this explanation is suitable for the Na- and K-based cells but not for the Li-based systems.

Using *operando* Raman spectroscopy, we studied the reasons of the rapid capacity fade in the Li-based cells during the deep cycling. It was previously suggested that this fast decay is related to decomposition of NiBTA upon deep reduction.^14^ However, the data show that no decomposition of lithiated NiBTA (Li_2_NiBTA) takes place. Instead, it is seen that conversion to the initial NiBTA phase upon reoxidation is incomplete, as follows from the spectra at 2.0 V after cycling (Fig. S45 and S46†). It indicates that the capacity fade is caused by hindered kinetics that leads to increased overpotentials. This could be due to formation of electrolyte decomposition products that impede diffusion or charge transfer. For the Na- and K-based cells, all changes in the Raman spectra are fully reversible (Fig. S45†), in accordance with their electrochemical output.

### 2.4. Comparison with previously proposed mechanisms

**Fig. 9:**
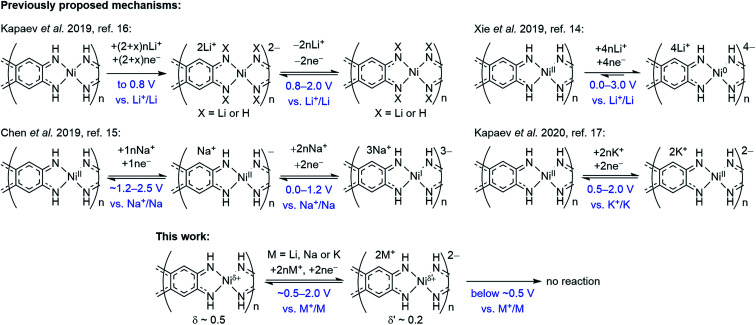
Charge storage mechanisms for NiBTA at the molecular level, as proposed in previous reports and in this work.

Overall redox reaction scheme for NiBTA that is proposed in this work is depicted in Fig. 9 along with the previously proposed mechanisms. The updated scheme is relying on the following facts:

• *Operando* Raman spectroscopy shows that the charge storage mechanism is similar for Li-, Na- and K-based cells at the molecular level.

• Raman spectroscopy and XRD reveal complete reversibility of NiBTA reduction. Therefore, no NH-proton substitution (as proposed by Kapaev *et al.*)^16^ takes place.

• NiBTA accepts only two electrons per repeating unit, as shown by Raman spectroscopy, XRD, XANES, and as suggested by electronic structure calculations. EDX mapping and post-mortem analysis show no signs of NiBTA decomposition at low potentials. No three- or four-electron reduction of the repeating units occurs, opposing the previously suggested reaction schemes for Li- and Na-based systems.^14,15^

• DFT-based population analysis shows that NiBTA reduction involves two-electron filling of the highest unoccupied LMO of the ligand (“working orbital”), whereas the formal oxidation state of nickel is zero and remains unchanged. The neutral formal charge of nickel is justified by intramolecular electron transfer from the working orbital, which is the HOMO of the BTA molecule. The natural atomic charge at Ni deviates from the formal charge, decreasing from *ca.* +0.5 to *ca.* +0.2 upon the two-electron reduction.

## 3. Conclusion

NiBTA shows promising features as an anode material for safe fast-charging lithium-, sodium- and potassium-ion batteries. In partially sodiated or potassiated states, the material undergoes fast bulk faradaic reactions, which should be beneficial for high-rate capabilities. The different behavior in the Li-, Na- and K-based systems is explained by the distinct crystal structure rearrangements. At the molecular level, the charge–discharge mechanism is similar for all types of batteries. It involves fully reversible ligand-based two-electron reduction, which is accompanied by the bandgap increase of the polymer. No further reduction of NiBTA occurs even at ∼0 V *vs.* M^+^/M. The reported results provide a deep insight into the redox chemistry of an emerging class of π–d coordination polymers.

## Data availability

The data supporting the findings of this study are available within the article and in the ESI.† The raw data are available from the authors upon reasonable request.

## Author contributions

RRK, KJS and AZ conceived the project. RRK performed the synthesis and all experimental studies except for the XANES measurements, which were carried out by DN. AZ performed all theoretical studies except for calculating charge distributions and band structures of crystalline polymers, which was carried out by DAA. SVR and DN analyzed the XANES data. PIM supervised the XANES measurements. KJS supervised the project. RRK and AZ wrote the manuscript with input from other co-authors. All authors gave approval to the final version of the paper.

## Conflicts of interest

There are no conflicts to declare.

## Supplementary Material

SC-013-D2SC03127B-s001

SC-013-D2SC03127B-s002

SC-013-D2SC03127B-s003

SC-013-D2SC03127B-s004

SC-013-D2SC03127B-s005

SC-013-D2SC03127B-s006

SC-013-D2SC03127B-s007

SC-013-D2SC03127B-s008

SC-013-D2SC03127B-s009

SC-013-D2SC03127B-s010

SC-013-D2SC03127B-s011

SC-013-D2SC03127B-s012

SC-013-D2SC03127B-s013

SC-013-D2SC03127B-s014

SC-013-D2SC03127B-s015

SC-013-D2SC03127B-s016

SC-013-D2SC03127B-s017

SC-013-D2SC03127B-s018

SC-013-D2SC03127B-s019

SC-013-D2SC03127B-s020

SC-013-D2SC03127B-s021

SC-013-D2SC03127B-s022

SC-013-D2SC03127B-s023

SC-013-D2SC03127B-s024
