# Supplementary material for: Charge storage mechanisms of a π–d conjugated polymer for advanced alkali-ion battery anodes
Source: Chem Sci. 2022 Jun 29;13(27):8161–70. doi: 10.1039/d2sc03127b (PMC9278342; doi:10.1039/d2sc03127b)
Supplement: SC-013-D2SC03127B-s024 [file SC-013-D2SC03127B-s024.pdf]

Electronic Supplementary Material (ESI) for Chemical Science.
This journal is © The Royal Society of Chemistry 2022

Supplementary Information

Charge storage mechanisms of a π-d conjugated polymer for advanced alkali-ion battery anodes

Roman R. Kapaev,[1] Andriy Zhugayevych,[1, 2] Sergey V. Ryazantsev,[1] Dmitry A. Aksyonov,[1] Daniil Novichkov,[3] Petr I. Matveev[3] and Keith J. Stevenson[1]*

[1] Center for Energy Science and Technology, Skolkovo Institute of Science and Technology, Bolshoy Boulevard 30 bld. 1, 121205 Moscow, Russia

[2] Polymer Theory Department, Max Planck Institute for Polymer Research, Ackermannweg 10, Mainz, 55128 Germany

[3] Department of Chemistry, Lomonosov Moscow State University, Leninskie Gory 1/3, Moscow, 119991 Russia

*E-mail: roman.kapaev@skoltech.ru

Contents:

List of Figures 2
List of Tables 4
Abbreviations 5
S1 Experimental 6
S2 Geometry of isolated macromolecules 11
S3 Electronic structure of standalone NiBTA macromolecules 12
S4 Population analysis 22
S5 Contributions from carbon black to the capacity 28
S6 Summary for the rate capabilities of NiBTA 32
S7 Rate capabilities of NiBTA in Li-based cells with different electrolytes 33
S8 Analysis of cyclic voltammetry data for NiBTA 34
S9 Simulated crystal structures and XRD studies 35
S10 Electronic structure of simulated crystalline polymers 48
S11 Raman spectra of NiBTA before and after cycling 50
S12 Conductivity measurements 53
S13 Raman spectra of NiBTA after chemical reduction 54
S14 Simulations of UV-Vis-NIR spectra 55
S15 Calculated Raman activities 57
S16 Charge distributions in crystal structures 58
S17 Electrochemistry in the 0.01–2.0 V vs. M^+/M potential ranges 59
S18 Raman spectroscopy in the 0.01–2.0 V vs. M^+/M potential ranges 60
S19 TEM EDX data before and after deep reduction 61
S20 Benchmarking for diradical-type oligomer Ni-OPD_2 62
S21 Geometric definitions used for the calculations 67
References 70

List of Figures

Figure S1. Geometry of isolated NiBTA macromolecules in pristine and lithiated/sodiated/potassiated states from top and side views. 11
Figure S2. Schematic electronic structure of the pristine NiBTA macromolecule. 12
Figure S3. Electronic bands of pristine NiBTA macromolecule. 14
Figure S4. Wave-function of the lowest unoccupied band of pristine NiBTA (“working” band) at Γ- and X-points. 14
Figure S5. Frontier localized molecular orbitals of the BTA monomer of the pristine polymer 16
Figure S6. Frontier MOs of the BTA molecule. 17
Figure S7. The Ni-N bonding and antibondng MOs at Γ-point. 17
Figure S8. Frontier molecular orbitals of the isolated lithiated macromolecule (Γ-point). 17
Figure S9. Electronic density of states for NiBTA calculated using various methods. 18
Figure S10. Electronic band structure of the standalone pristine macromolecule calculated using various methods. 19
Figure S11. Comparison of LMOs obtained by variation of localization procedure. 21
Figure S12. Orientation and atom indexing of the NiBTA low-molecular model used for population analysis. 22
Figure S13. Orientation and atom indexing of the molecO5 used for population analysis. 25
Figure S14. Electrochemistry of Super P in lithium-based cells in the 0.5–2.0 V vs. Li^+/Li potential range. 28
Figure S15. Electrochemistry of Super P in sodium- and potassium-based cells in the 0.5–2.0 V vs. M^+/M potential ranges. 29
Figure S16. Electrochemistry of Super P in lithium-based cells in the 0.01–2.0 V vs. Li^+/Li potential range. 30
Figure S17. Electrochemistry of Super P in sodium- and potassium-based cells in the 0.01–2.0 V vs. M^+/M potential ranges. 31
Figure S18. Capacity vs. current density calculated per NiBTA mass for various types of cells (a) and NiBTA capacity after subtracting the contribution from Super P (b). 32
Figure S19. Electrochemistry of NiBTA in Li-based cells with different electrolytes 33
Figure S20. Potential differences between oxidation and reduction CV peaks at the scan rate of 0.2 mV s^{-1}. 34
Figure S21. *I-v* analysis for NiBTA. 34
Figure S22. Different types of simulated crystal structures. 36
Figure S23. Simulated crystal structures of NiBTA that are most energetically favorable (herringbone-type structures). 39
Figure S24. Simulated crystal structures of NiBTA that are less energetically favorable (brickwork and in-plane structures). 40
Figure S25. Various simulated crystal structures of fully lithiated/sodiated/potassiated derivatives of NiBTA. 41
Figure S26. XRD pattern of NiBTA compared to XRD patterns of herringbone-type simulated structures (CoKα radiation, λ = 1.78892 Å). 42
Figure S27. XRD pattern of NiBTA compared to XRD patterns of stacked-type simulated structures (CoKα radiation, λ = 1.78892 Å). 43
Figure S28. XRD pattern of lithiated NiBTA (0.5 V vs. Li^+/Li) compared to XRD patterns of simulated structures (CoKα radiation, λ = 1.78892 Å). 44

Figure S29. XRD pattern of sodiated NiBTA (0.5 V vs. Na^+/Na) compared to XRD patterns of simulated structures (CoKα radiation, λ = 1.78892 Å). 45
Figure S30. XRD pattern of potassiated NiBTA (0.5 V vs. Na^+/Na) compared to XRD patterns of simulated structures (CoKα radiation, λ = 1.78892 Å). 46
Figure S31. Stable positions of alkali atoms near oligomers. 47
Figure S32. Electronic density of states of simulated structures. 48
Figure S33. Band structure for crystalline NiBTA (cryst-hc) (a) and Li_2NiBTA (crystLiLi-h) (b). 49
Figure S34. Normalized Raman spectra of NiBTA in the lithium-based cell before and after cycling in the 0.5–2.0 V vs. Li^+/Li potential range. 50
Figure S35. Normalized Raman spectra of NiBTA in the sodium-based cell before and after cycling in the 0.5–2.0 V vs. Na^+/Na potential range. 51
Figure S36. Normalized Raman spectra of NiBTA in the potassium-based cell before and after cycling in the 0.5–2.0 V vs. K^+/K potential range. 52
Figure S37. Direct-current polarization data for a cylindrical pellet of NiBTA. 53
Figure S38. Raman spectra of NiBTA-based films before and after chemical reduction measured with the green (left) and near-infrared (right) lasers. 54
Figure S39. Typical spectra of excitations. 55
Figure S40. Calculated bright excitation energy for oligomers of various length. 55
Figure S41. Calculated Raman activities for oligomers. 57
Figure S42. Differential charge density dn = n(Li_2NiBTA) - n(NiBTA) drawn for isosurface levels of 0.0085 (a) and 0.006 (b). 58
Figure S43. Electrochemistry of NiBTA in Na- and K-based cells in the 0.01–2.0 V vs. M^+/M potential ranges. 59
Figure S44. Electrochemistry of NiBTA in Li-based cells in the 0.01–2.0 V vs. Li^+/Li potential range. 59
Figure S45. Evolution of Raman spectra of NiBTA in the 0.01–2.0 V vs. M^+/M potential ranges for Li- (a), Na- (b) and K-based (c) cells. 60
Figure S46. *Operando* Raman spectra of NiBTA in the Li-based cell at 2.0 V vs. Li^+/Li before and after cycling. 60
Figure S47. EDX data of NiBTA before/after deep cycling in Li-based cells. 61
Figure S48. Orientation and atom indexing of Ni-OPD_2. 62
Figure S49. Frontier molecular orbitals of Ni-OPD_2. 62
Figure S50. Relaxed volume scan for determination of parameters of equation of state of the crystal. 65
Figure S51. Orientation and atom indexing of NiBTA used for the calculations. 67
Figure S52. Considered terminations of NiBTA oligomers. 68

List of Tables

Table S1. Tight-binding Hamiltonian (in eV) and overlap matrices for the nearest and next nearest neighbor LMOs shown in **Figure S2**. 13
Table S2. List of frontier electronic bands of the standalone pristine macromolecule including all π-conjugated LMOs and LPs. 15
Table S3. Energies of core MOs (in eV) for the standalone macromolecule in the pristine and reduced states. 20
Table S4. Natural atomic orbital (NAO) populations for pristine and doubly charged oligomer shown in **Figure S12**, compared also with the BTA molecule shown in **Figure S6**. 23
Table S5. Natural atomic orbitals in details. 23
Table S6. Natural atomic charges for oligomers of various length, termination, and oxidation state. 24
Table S7. Natural atomic charges and orbital (NAO) populations for pristine and doubly fully reduced NiBTA oligomers shown in **Figure S13**. 25
Table S8. Natural atomic orbitals for pristine and reduced NiBTA models shown in **Figure S13**. 26
Table S9. Types of NiBTA structures considered for simulations. 35
Table S10. Structures predicted by PBE-D3/PAW600 method. 37
Table S11. The most intensive reflections in simulated powder diffraction patterns for predicted structures. 38
Table S12. Possible configurations of K atoms attached to the oligomer shown in **Figure S31** at positions 1-7. 47
Table S13. Bader atomic charges calculated for crystalline NiBTA (cryst-hc, **Figure S23**) and Li_2NiBTA (crystLiLi-h, **Figure S25**). 58
Table S14. Methods benchmarking for IP and EA of the neutral singlet state. 63
Table S15. Methods benchmarking for singlet excitations of the neutral singlet state. 63
Table S16. Benchmarking methods for spin gaps with relaxed geometries at mmm-symmetry. 63
Table S17. Methods benchmarking for Raman active modes. 64
Table S18. Crystal geometry: experiment at 295 K vs. calculated (at 0 K). 65
Table S19. Summary table for vibrations of the crystal (Γ-point only). 66

Abbreviations

a3p	Ahlrichs triple-ζ basis Def2-TZVP
AO	atomic orbital
AMU	atomic mass unit
BLA	bond length alternation
BTA	1,2,4,5-benzenetetramine
CPCM	conduct. polarizable continuum model
CV	cyclic voltammetry
DFT	density functional theory
DME	1,2-dimethoxyethane
DMC	dimethyl carbonate
DOL	1,3-dioxolane
EA	electron affinity
EC	ethylene carbonate
EDX	energy dispersive X-ray analysis
HAADF	high-angle annular dark field
HOMO	highest occupied molecular orbital
IP	ionization potential
LiTFSI	lithium bis(trifluoromethanesulfonyl)imide
LMO	localized molecular orbital
LP	lone pair
LUMO	lowest unoccupied molecular orbital
MD	molecular dynamics
MO	molecular orbital
NAO	natural atomic orbital
NBO	natural bonding orbital
NO	natural orbital
NTO	natural transition orbital
OPD	o-phenylenediamine
PAW	projector augmented wave
PES	potential energy surface
p2p	Pople double-ζ polarized basis 6-31G*
p2p/a3p	p2p for H to Ar, a3p for heavier elements
SCF	self-consistent field
STEM	scanning transmission electron microscopy
TEM	transmission electron microscopy
TDDFT	time dependent DFT
UV-Vis-NIR	ultraviolet-visible-near infrared
XANES	X-ray absorption near edge structure
XRD	X-ray diffraction
ZPE	zero-point energy

S1 Experimental

NiBTA synthesis. The polymer was synthesized according to a previously published procedure [Kapaev2019]. XRD pattern of NiBTA was in agreement with the literature data.

Electrode preparation. For the electrochemical tests, 70% wt. of NiBTA, 15% wt. of Super P carbon black and 15% wt. of poly(vinylidene difluoride) were thoroughly mixed in *N*-methylpyrrolidone to form a homogeneous slurry, which was tape-casted onto a carbon-coated copper foil. The deposited slurry was evaporated in air at 70 °C, the electrodes were vacuum-dried at 110 °C for ~5 h, calendered at room temperature, and further vacuum-dried at 110 °C for ~2 h. NiBTA mass loading was 0.9 mg cm^{-2} for the galvanostatic experiments and 0.5 mg cm^{-2} for the cyclic voltammetry measurements.

To estimate the capacity contribution of Super P, electrodes containing 70% wt. of Super P and 30% wt. of poly(vinylidene difluoride) were prepared using the same tape-casting method with a carbon-coated copper current collector. The mass loading of Super P was 1.1 mg cm^{-2}.

For the XRD and XANES measurements, a homogeneous slurry containing 70% wt. of NiBTA, 15% wt. of Super P and 15% wt. of poly(vinylidene difluoride) dissolved in *N*-methylpyrrolidone was tape-casted onto bare aluminum foil. Thickness of the slurry layer was 600 μm. The slurry was air-dried at 70 °C and then vacuum-dried at 110 °C overnight. After calendaring at room temperature, the electrode delaminated from the Al foil. Round free-standing electrodes (d = 16 mm) were made and further dried at 110 °C overnight. NiBTA mass loading was from 7.4 to 7.7 mg cm^{-2}.

For the Raman spectroscopy measurements, a homogeneous H_2O-based slurry containing 80% wt. of NiBTA, 10% wt. of Super P and 10% wt. of sodium carboxymethylcellulose was tape-casted onto carbon-coated copper foil. The electrode was dried in air at 40 °C, then vacuum-dried at 110 °C for ~5 h and calendered at room temperature. NiBTA mass loading was ~1.0 mg cm^{-2}. Rectangular electrodes (~1 cm long, ~1 mm wide) were prepared.

Cell assembling. All procedures were performed in an argon-filled glovebox with O_2 and H_2O levels below 1 and 0.1 ppm, respectively.

For studying the electrochemical properties, coin cells (CR2032) were assembled. Alkali metals (Li, Na or K) were used as counter-electrodes, glass fiber was used for the separators. For the Li-based cells, the electrolytes were 1M $LiPF_6$ in EC:DMC (1:1 v/v) or 1M LiTFSI in DME:DOL (1:1 v/v). For the Na- and K-based cells, the electrolytes were 1.5M $NaPF_6$ in DME and 1.5M KPF_6 in DME, respectively. Each cell contained 70–80 μL of the electrolyte.

For the *ex situ* XRD and XANES measurements, disassemblable cells (ECC-Ref from EL-CELL) were assembled in a two-electrode configuration. Li, Na or K metals were used as the counter electrodes. Glass fiber separators were used, and additional layers of polyolefin-based separators were placed on top of the working electrodes to avoid sticking of the glass fiber to the electrodes. The electrolytes for the Li-based, Na-based and K-based cells were 1M $LiPF_6$ in EC:DMC (1:1 v/v), 1.5M $NaPF_6$ in DME and 1.5M KPF_6 in DME, respectively.

For the *operando* XRD measurements, a Swagelok-type cell with a Be window [Leriche2010] was assembled. Li, Na or K metals were used as the counter electrodes. Three layers of the glass fiber separators were placed between the electrodes to avoid short-circuiting of the cell because of the alkali metal dendrite growth. The electrolytes for the Li-

based, Na-based and K-based cells were 1M $LiPF_6$ in EC:DMC (1:1 v/v), 1.5M $NaPF_6$ in diglyme and 1.5M KPF_6 in diglyme, respectively. The cell contained 240 μL of the electrolyte.

For the *operando* Raman spectroscopy measurements, a cell with an optically transparent window (ECC-Opto-Std from EL-CELL) was assembled in a two-electrode configuration. The electrodes were placed in direct contact with the optically transparent window (sapphire of mineral glass), with the active material facing the window. Li, Na or K metals were used as the counter electrodes. Three layers of glass fiber separators were used. The electrolytes for the Li-based, Na-based and K-based cells were 1M $LiPF_6$ in EC:DMC (1:1 v/v), 1.5M $NaPF_6$ in diglyme and 1.5M KPF_6 in diglyme, respectively.

Galvanostatic cycling and capacity calculations. The experiments were performed with Neware BTS-4000 stations at room temperature. The current densities were set to 0.1, 0.2, 0.5, 1, 2 or 5 A g^{-1}. The potential ranges were 0.5–2.0 V or 0.01–2.0 V vs. M^+/M (M = Li, Na or K). Prior to the prolonged cycling at 2 A g^{-1}, the cells were subjected to five cycles at 0.1 A g^{-1} to avoid low initial capacities associated with the activation effects [Kapaev2019]. The current densities and capacities were calculated basing on the mass of NiBTA unless stated otherwise.

Calculating capacity contributions from carbon black. Specific capacities of Super P carbon black are shown in **Figure S14**, **Figure S15**, **Figure S16** and **Figure S17**. Contributions from carbon to the capacities of the NiBTA-based electrodes were calculated as follows:

$$Q_{\mathrm{corr}} = Q_{\mathrm{NiBTA}} - \frac{\omega_{\mathrm{SP}}}{\omega_{\mathrm{NiBTA}}} Q_{\mathrm{SP}} \quad (1)$$

where Q_{corr} is the capacity after subtracting the contribution from Super P, Q_{NiBTA} is the capacity per NiBTA mass, Q_{SP} is the capacity of Super P in the control experiments measured at the same current density in the same potential range, ω_{SP} and ω_{NiBTA} are mass fractions of Super P and NiBTA in the electrode, respectively.

Cyclic voltammetry. The experiments were performed with BioLogic VMP3 at room temperature. The potential ranges were 0.5–2.0 V vs. M^+/M (M = Li, Na or K). Prior to the main experiments, ten scans at 1 mV s^{-1} were carried out to eliminate irreversible processes. The current densities were calculated basing on the mass of NiBTA.

Sample preparation for the *ex situ* XRD measurements. The electrodes were discharged at room temperature to 0.5 V or 0.01 V vs. M^+/M (M = Li, Na or K) at a constant current density of 30 mA g^{-1}. The cells were disassembled in an argon-filled glovebox, the electrodes were then washed with ~4 mL of DME and dried in the glovebox environment at room temperature. The electrodes were placed onto a Mylar film in a sample holder for the Huber Guinier Camera 670, and the holder was sealed with a Kapton tape to prevent decomposition in air during the measurements. The sample was then taken out from the glovebox.

***Ex situ* XRD measurements.** The XRD patterns were measured with a Huber Guinier Camera 670 that operates with $CoK_{\alpha 1}$ radiation (λ = 1.78892 Å). The measurements were carried out directly after the Kapton-sealed electrodes were removed from the glovebox. Single scans were measured in the 2θ range of 4–100° each ten minutes to monitor possible changes caused by oxidation. The resolution was 0.005°. The measurements continued overnight. The scans that were measured before changes in the XRD patterns started to occur were then averaged.

***Operando* XRD measurements.** The cell for the XRD measurements was placed inside a Bruker D8 ADVANCE powder X-ray diffractometer that operates with CuK_{α} radiation. The cell was connected to BioLogic SP-150. The XRD patterns were collected at room temperature during galvanostatic discharging to 0.5 V vs. M^+/M (M = Li, Na or K) and subsequent charging to 2.0 V M^+/M. The current density was 30 mA g^{-1} basing on the mass of NiBTA. Single scans were measured each 10 minutes, the resolution was 0.04°, the 2θ range was 12-32° for the Li-based cell and 14–38° for the Na- and K-based cells. The XRD patterns were baseline-corrected with Bruker DIFFRAC.EVA software.

Sample preparation for the *ex situ* XANES measurements. The electrodes were discharged at room temperature to 0.5 V or 0.01 V vs. M^+/M (M = Li, Na or K) at a constant current density of 30 mA g^{-1}. The cells were disassembled in an argon-filled glovebox and sealed between two layers of Kapton tape. Pristine electrode was sealed between Kapton layers as well.

***Ex situ* XANES measurements.** The XANES spectra were obtained with a laboratory spectrometer of the Department of Radiochemistry, Moscow State University. An X-ray tube with a silver anode with a power of 1.5 kW was used as an X-ray source. The X-ray tube, monochromator crystal, and silicon drift detector (Amptek) were placed in a Rowland circle, 0.5 m in diameter. The radiation energy was chosen using the reflection [444] of a spherically bent silicon crystal (with a bend radius of 0.5 m) oriented at a Bragg angle of 71.6°. To avoid damage to the samples by the white beam, the samples were placed in front of the detector where they were exposed only to the monochromatic beam. The beam size was 5 mm by 5 mm. The data were collected in the transmission mode, at each energy point of the XANES region the signal was accumulated for 5 s. For all samples, 5 spectra were collected and combined using the IFEFFIT software.

***Operando* Raman spectroscopy measurements.** The cell for Raman spectroscopy measurements was placed in a Thermo Scientific DXRxi Raman Imaging microscope and connected to BioLogic SP-150. The spectra were collected at room temperature during CV measurements in the potential ranges of 0.5–2.0 V or 0.01–2.0 V vs. M^+/M (M = Li, Na or K). The potential scan rate was 0.047 mV s^{-1}. Prior to the CV measurements, linear sweep voltammetry from open-circuit voltage to 2.0 V vs. M^+/M was performed with the scan rate of 1 mV s^{-1}. The laser excitation wavelength λ was 532 nm or 780 nm, the laser power was 1 mW. Single scans were measured every 7 minutes (~20 mV). The range of Raman shifts was 50–2,200 cm^{-1} (for λ = 780 nm) or 50–3,400 cm^{-1} (for λ = 532 nm). The spectra were baseline-corrected with Bruker OPUS software.

Sample preparation for UV-Vis-NIR spectroscopy measurements. To prepare the NiBTA-based films, 36 mg of NiBTA and 4 mg of sodium carboxymethylcellulose were dispersed in 4 mL of deionized H_2O *via* ultrasonication. The suspension was deposited with a spin-coater onto glass substrates (~2x2 cm pieces, 600 μL per piece). The rotation rate of the spin-coater was 2,000 rpm. The alkaliation solution was prepared in an argon-filled glovebox. Naphthalene (0.8 mmol, 103 mg) was dissolved in diglyme (8 mL). Metallic potassium (~50–100 mg) was introduced to the solution. The mixture was stirred at room temperature for 2 h, resulting in a dark-green solution of potassium naphthalenide. Excess of potassium was removed afterwards. The NiBTA-based film was introduced to the pre-alkaliation solution and kept at room temperature for 2 days in an argon-filled glovebox. The film was removed from the solution, washed with ~4 mL of DME and dried in an argon-filled glovebox at room temperature.

UV-Vis-NIR spectroscopy measurements. The spectra were collected in transmittance mode using an AvaSpec-2048-2 fiber-optic spectrometer that was placed in a N_2-filled glovebox. The samples were transferred directly from an Ar-filled glovebox in a sealed vial.

Pellet preparation and conductivity measurements. Cylindrical pellets of NiBTA (d = 10 mm) were prepared by cold pressing of the powder (146 mg) with commercial pressing equipment (Carver). Applied load was 5 metric tons. Prior to the conductivity measurements, top and bottom of the pellet were coated with 30–50 nm of gold using a Quorum Q150T ES magnetron. Sides of the pellet were covered with an adhesive tape before the sputtering. The coated pellet was placed in a symmetrical cell between two copper disks. Direct-current polarization was applied using BioLogic VMP3. The voltage was changed in 50 mV steps between −1 and 1 V, the current was measured over 5 s at each step.

Microscopy studies and elemental analysis. The microscopy and energy-dispersive X-ray spectroscopy measurements were performed with a Titan Themis Z transmission electron microscope at 200 kV. Samples were ground in a mortar in an organic solvent (ethanol for the pristine electrode, anhydrous dimethyl carbonate for the cycled electrodes) and dispersed onto Cu square mesh grid covered with holey carbon. The electrodes after cycling were prepared in an Ar-filled glovebox and transferred to the TEM chamber with a short air exposure of 1–2 seconds. Standard double tilt TEM holder was used. Imaging mode was high-angle annular dark field (HAADF) STEM, using HAADF detector at an effective camera length of 115 mm. Super-X EDX detector was used for the EDX mapping. Thermo Fisher Scientific Velox software was used both for visualization and analysis of EDX data.

Computations. For calculations of oligomers and standalone polymer, we used CAM-B3LYPp2p/a3p method as implemented in Gaussian 16 package. The CAM-B3LYP functional [Yanai2004] was chosen because it has the lowest mismatch between orbital and total energies, see **Table S14**, and proven reliability for π-conjugated molecules [Tukachev2019, Zhugayevych2018]. Comparison with other functionals was performed for critical calculations, at least with PBE0 which is more reliable beyond the class of π-conjugated semiconductors [Vasilchenko2021]. The basis 6-31G*, referred here as 'p2p', commonly used for light elements is unreliable for heavier ones. For them we used a more robust Def2-TZVP basis abbreviated as 'a3p'. Because scalability is critical, we used 'p2p' basis for light elements up to Ar and 'a3p' for heavier ones, denoting this combination as 'p2p/a3p'. It should be noted that the standard 'p2p' basis uses 6 d-orbitals, whereas in the 'p2p/a3p' combination every l-shell contains $2l + 1$ orbitals.

For calculations of crystals, we used PBE-D3/PAW600 method as implemented in VASP 5.4 package, here 600 means 600 eV plane wave energy cutoff. Despite PBE functional gives typically inaccurate electronic structure of π-conjugated systems, the combination PBE-D3 is robust for prediction of intermolecular packing [Halaby2021, Zhugayevych2018]. Use of hybrid functionals is limited due to poor convergence of wave-function for all considered basis sets and programs. PBE/PAW600 was also used for calculation of differential charge density, Bader charges [Tang2009] and band structure [Ong2013] of crystal structures.

Localization of molecular orbitals is performed by projection onto basis of selected block of atoms using "MolMod:-LocalizeMO" program which code is available at http://zhugayevych.me/maple/MolMod/MolMod.ini, see **Figure S11** for details.

In all figures of the current work, the wave-function isovalue is typically 0.05 for LMO and 0.02 for MO. In case of comparison, all figures are plotted with the same isovalue.
Powder diffraction patterns for predicted structures were simulated in VESTA program.
Computational spectroscopy was performed for oligomers. While intramolecular vibrational frequencies depend on the local atomic environment, allowing reliable modeling with oligomers, absorption spectra and Raman intensities of “soft” materials require statistical sampling over large ensembles. Therefore, considered oligomer models provide only interpretation of the experimental spectra. For excited states, we used TDDFT.
Benchmarking of the computations was performed using Ni-OPD_2 as a model compound. The benchmarking results are available in Section S20, p. **62**. Geometric definitions used for the calculations are provided in Section S21, p. **67**.

S2 Geometry of isolated macromolecules

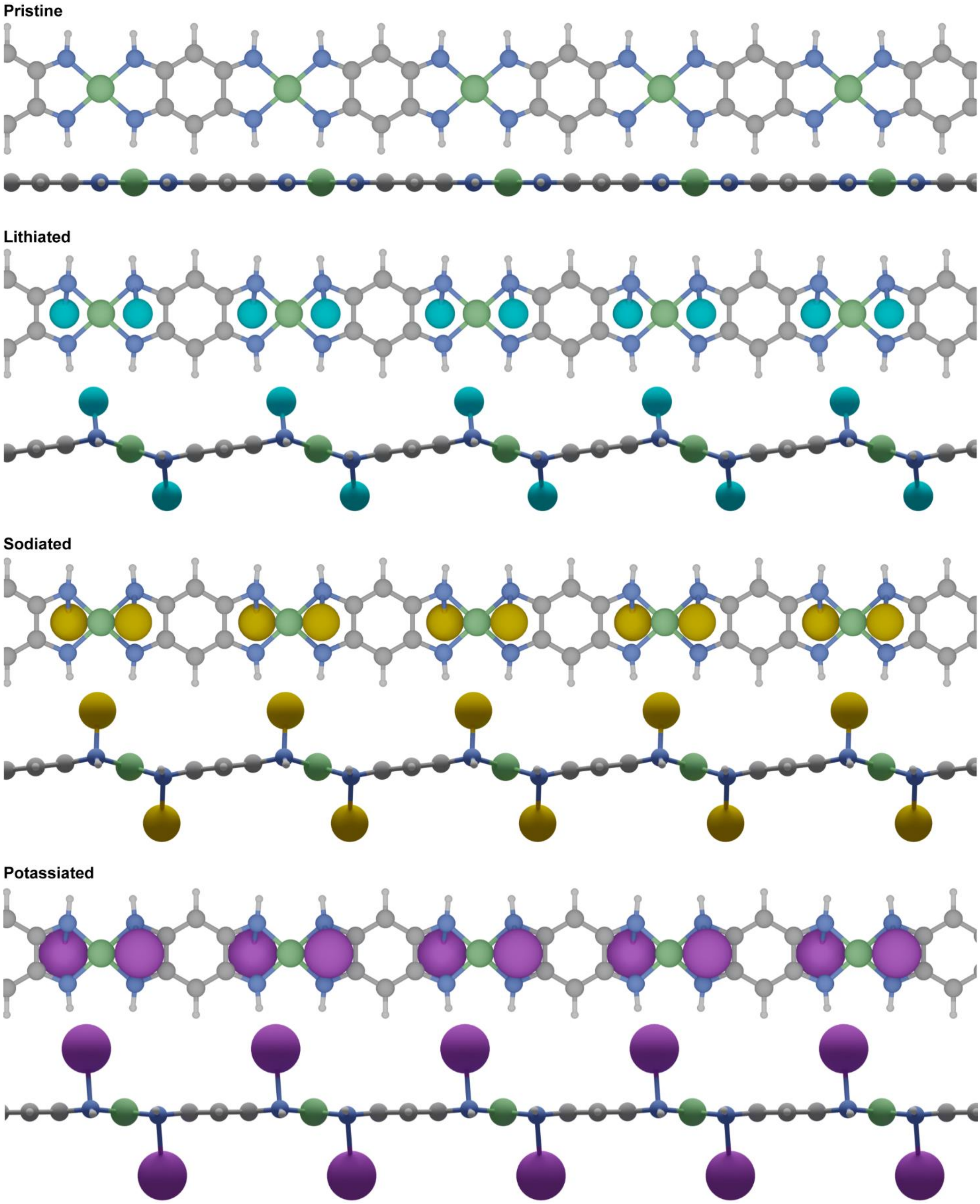

Figure S1. Geometry of isolated NiBTA macromolecules in pristine and lithiated/sodiated/potassiated states from top and side views. Crystal structures are shown in **Figure S23** and **Figure S25**.

S3 Electronic structure of standalone NiBTA macromolecules

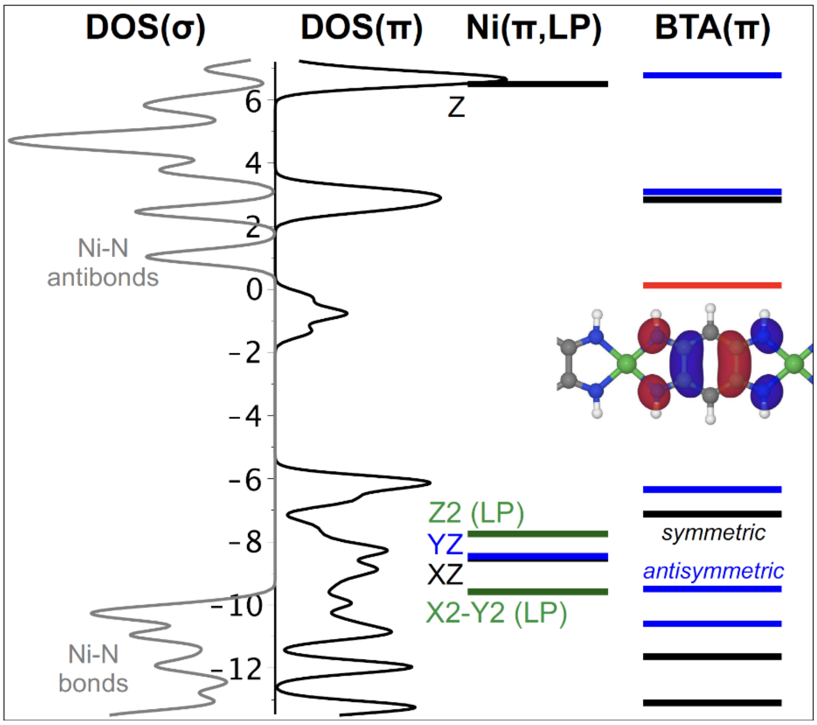

Figure S2. Schematic electronic structure of the pristine NiBTA macromolecule. See also Figure 1 in the main text. The energy is in eV. The π-conjugated system is represented by DOS and by monomer LMOs: Ni (left) and BTA (right). The symmetric LMOs (black colored) belong to B1u (z) or B2g (xz) representations of mmm group, whereas antisymmetric LMOs (blue colored) belong to B3g (yz) or Au (xyz) representations. The two Ni LPs are shown in green. Their density is excluded from DOS(σ). The "working" LMO is shown in red: it is empty for the pristine polymer, but occupied upon 2e-reduction, and also it is occupied in the BTA molecule.

Table S1. Tight-binding Hamiltonian (in eV) and overlap matrices for the nearest and next nearest neighbor LMOs shown in **Figure S2**. The matrix elements are indexed by the first row and column containing energies of LMOs. For example, the "working" LMOs (0.112 eV) are coupled "through space" by transfer integral -0.167 eV and through Ni Z orbital (6.483 eV) by -1.663 eV.

Hamiltonian

.	-8.538	-8.472	6.483	-13.117	-11.647	-10.604	-9.515	-7.137	-6.354	0.112	2.824	3.074	6.772
-13.117	-0.042	.	.										
-11.647	-0.079	.	.										
-10.604	.	0.045	.										
-9.515	.	0.104	.										
-7.137	-0.356	.	.										
-6.354	.	0.206	.										
0.112	.	.	-1.663										
2.824	.	.	0.873										
3.074	.	.	.										
6.772	.	.	.										
-8.538	0.027	.	.										
-8.472	.	-0.002	.										
6.483	.	.	-0.010										
-13.117	-0.007	.	.	-0.009	0.011	.	.	-0.022	.	.	.	.	.
-11.647	-0.009	.	.	-0.011	0.009	.	.	-0.022	.	.	.	.	.
-10.604	.	0.001	.	.	.	0.034	-0.050	.	0.042	.	.	.	.
-9.515	.	0.001	.	.	.	0.050	-0.072	.	0.058	.	.	.	.
-7.137	-0.018	.	.	-0.022	0.022	.	.	-0.055	.	.	.	.	.
-6.354	.	0.001	.	.	.	0.042	-0.058	.	0.043	.	.	.	.
0.112	.	.	0.016	.	.	.	.	.	.	-0.167	-0.072	.	.
2.824	.	.	-0.005	.	.	.	.	.	.	0.072	0.024	.	.
3.074	.	.	.	.	.	.	.	.	.	.	.	0.154	-0.343
6.772	.	.	.	.	.	.	.	.	.	.	.	0.343	-0.487

Overlaps

.	-8.538	-8.472	6.483	-13.117	-11.647	-10.604	-9.515	-7.137	-6.354	0.112	2.824	3.074	6.772
-13.117	-0.037	.	.										
-11.647	-0.054	.	.										
-10.604	.	0.037	.										
-9.515	.	0.051	.										
-7.137	-0.044	.	.										
-6.354	.	0.042	.										
0.112	.	.	-0.163										
2.824	.	.	0.093										
3.074	.	.	.										
6.772	.	.	.										
-8.538	-0.013	.	.										
-8.472	.	0.003	.										
6.483	.	.	-0.002										
-13.117	0.002	.	.	-0.003	0.005	.	.	-0.008	.	.	.	.	.
-11.647	0.003	.	.	-0.005	0.009	.	.	-0.014	.	.	.	.	.
-10.604	.	.	.	.	.	-0.003	0.005	.	-0.006	.	.	.	.
-9.515	.	.	.	.	.	-0.005	0.008	.	-0.009	.	.	.	.
-7.137	0.007	.	.	-0.008	0.014	.	.	-0.020	.	.	.	.	.
-6.354	.	.	.	.	.	-0.006	0.009	.	-0.010	.	.	.	.
0.112	.	.	-0.002	.	.	.	.	.	.	-0.023	-0.005	.	.
2.824	.	.	.	.	.	.	.	.	.	0.005	0.002	.	.
3.074	.	.	.	.	.	.	.	.	.	.	.	0.030	-0.044
6.772	.	.	.	.	.	.	.	.	.	.	.	0.044	-0.054

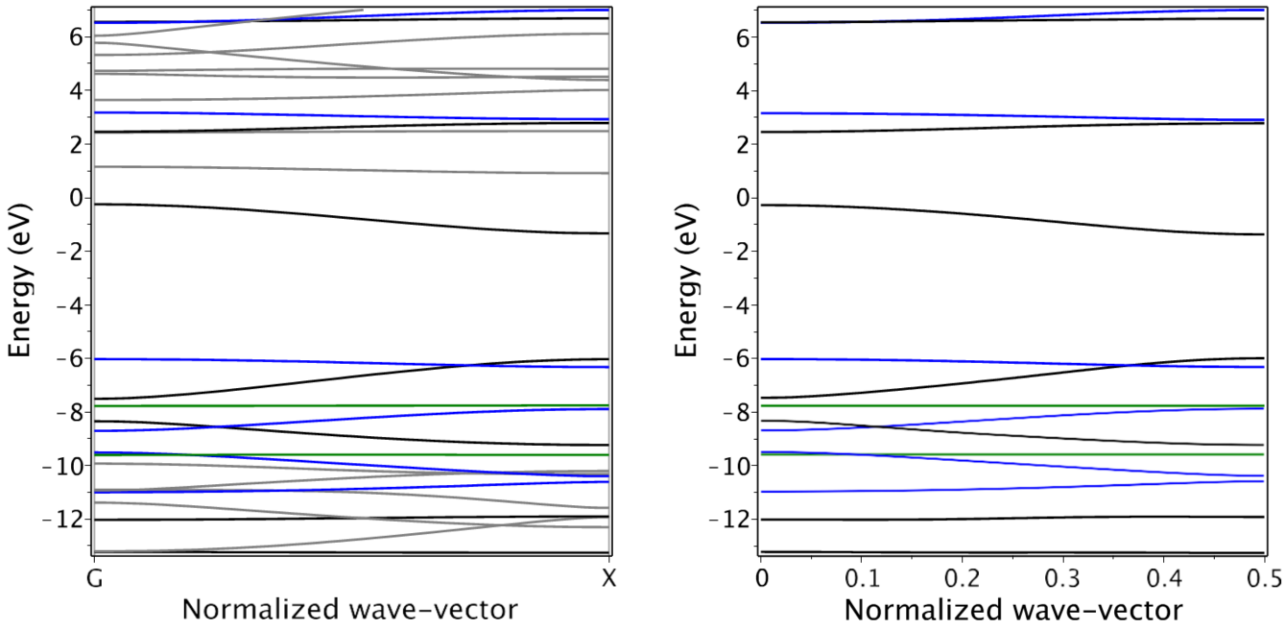

Figure S3. Electronic bands of pristine NiBTA macromolecule. Band structure for all electrons is shown on the left, the bands only for valence π-orbitals and Ni LPs are shown on the right. Color codes correspond to **Figure S2**. Band structures of crystalline polymers (**Figure S33**) reveal that pronounced dispersion of energy bands occurs only along the polymer chains, so considering isolated macromolecules is reliable for assessing the electronic structure of NiBTA. It is seen that width of frontier bands is smaller than ~1 eV, many of them are almost flat, and π-bands are not entangled in the sense that the wave-function pattern weakly depends on the k-vector. This allows to clearly see the constituting LMOs even visually (see **Figure S4**).

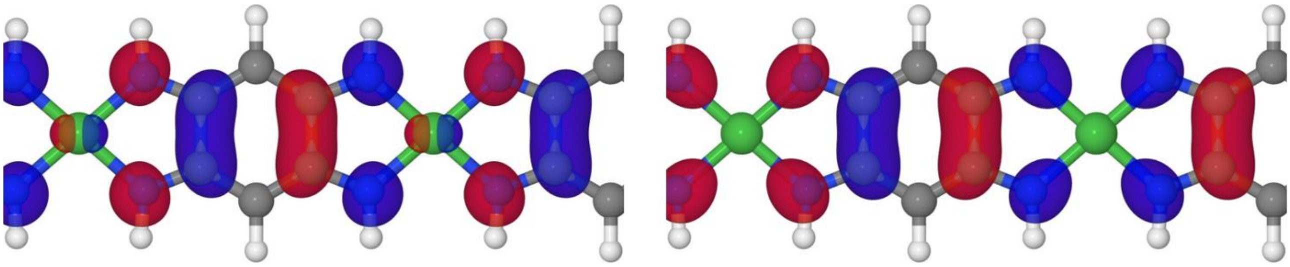

Figure S4. Wave-function of the lowest unoccupied band of pristine NiBTA ("working" band) at Γ- and X-points.

Table S2. List of frontier electronic bands of the standalone pristine macromolecule including all π-conjugated LMOs and LPs. The double horizontal line separates occupied and unoccupied bands. Bands are numbered from the bottom of the valence band according to their energy at Γ-point. The next three columns include energies in eV: the band energy at Γ- and X-points, E_{Γ} and E_{X} (X means end of the first Brillouin zone), and the LMO energy, E_{LMO}. In 'type' column the antibonding asterisk is omitted for clarity, and prime labels blue-colored orbitals in **Figure S2** and **Figure S3**. LMOs are numbered starting from 1 within their groups: π-BTA, π-Ni, LP-Ni. The π-system includes LMOs of 4 symmetries: Au (xyz), B1u (z), B2g (xz), B3g (yz); the other 4 symmetries describe σ-system: Ag (1), B1g (xy), B2u (y), B3u (x). Symmetry notations for σ-bands correspond to the wave-function symmetry at Γ-point around Ni atom (the same symmetry is at X-point).

#	E_{Γ}	E_{X}	E_{LMO}	type	LMO#	symmetry	description
16	-13.23	-13.26	-13.12	π-BTA	1	B1u	
17	-13.20	-11.92		σ		X	
18	-12.02	-11.90	-11.65	π-BTA	2	B2g	
19	-11.38	-12.29		σ		XY	NiN bonds
20	-10.99	-10.61	-10.60	π-BTA'	3	B3g	
21	-10.93	-10.20		σ		Y	
22	-10.90	-11.58		σ		X2	
23	-9.92	-10.31		σ		X	highest occupied σ-band
24	-9.60	-9.60	-9.60	LP-Ni	1	Ag	Ni e_2 LP (X2-Y2)
25	-9.51	-10.40	-9.51	π-BTA'	4	Au	
26	-8.70	-7.89	-8.47	π-Ni'	2	B3g	Ni YZ
27	-8.34	-9.23	-8.54	π-Ni	1	B2g	Ni XZ
28	-7.77	-7.75	-7.76	LP-Ni	2	Ag	Ni e_2 LP (Z2)
29	-7.51	-6.03	-7.14	π-BTA	5	B1u	
30	-6.02	-6.32	-6.35	π-BTA'	6	B3g	
31	-0.25	-1.34	0.11	π-BTA	7	B2g	"working" band
32	1.15	0.91		σ		XY	NiN antibonds
33	2.43	2.48		σ		1	diffuse orbitals
34	2.46	2.79	2.82	π-BTA	8	B1u	
35	3.17	2.93	3.07	π-BTA'	9	Au	
36	3.65	4.02		σ		Y	
. . .	. . .	. . .		σ			
43	6.56	6.69	6.48	π-Ni	3	B1u	Ni Z
42	6.52	7.00	6.77	π-BTA'	10	B3g	

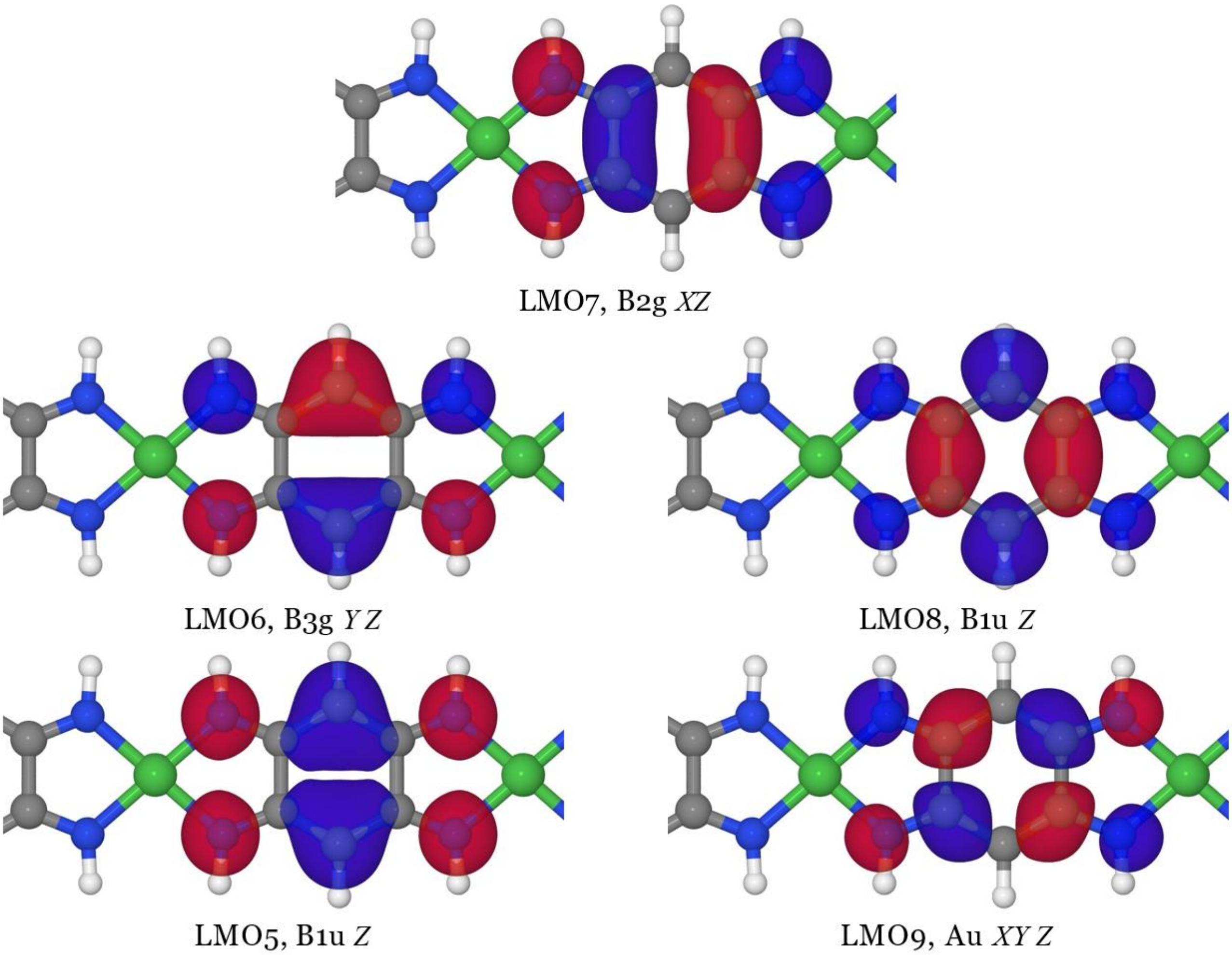

Figure S5. Frontier localized molecular orbitals of the BTA monomer of the pristine polymer. The LMO7 is empty for pristine NiBTA and occupied for charged NiBTA as well as for BTA molecule (each nitrogen has two hydrogens).

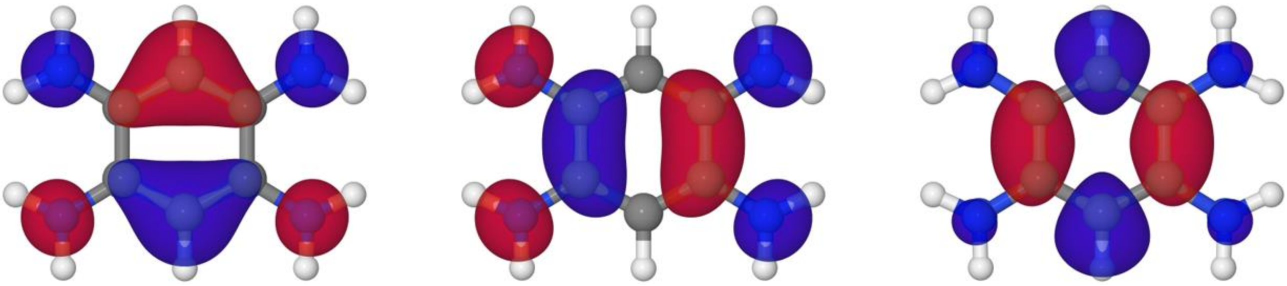

Figure S6. Frontier MOs of the BTA molecule. From left to right: HOMO-1, HOMO, LUMO, matching LMO6, LMO7, LMO9 from **Figure S5**. The planar conformer is shown here which is 0.84 eV higher in energy than the lowest energy conformer with slightly buckled NH_2 groups.

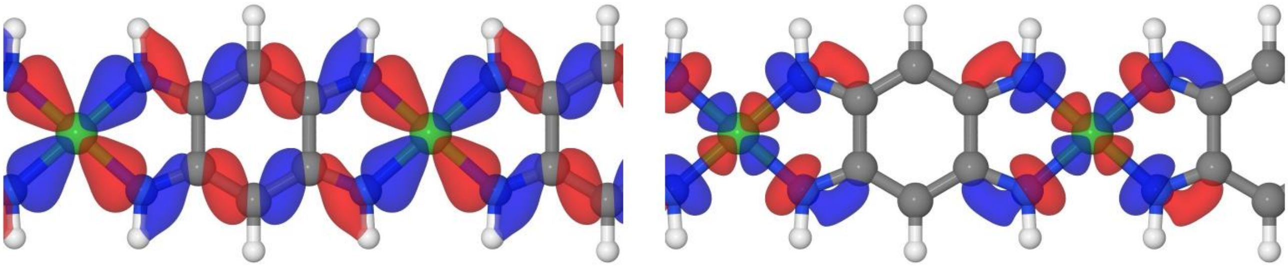

Figure S7. The Ni-N bonding and antibondng MOs at Γ-point.

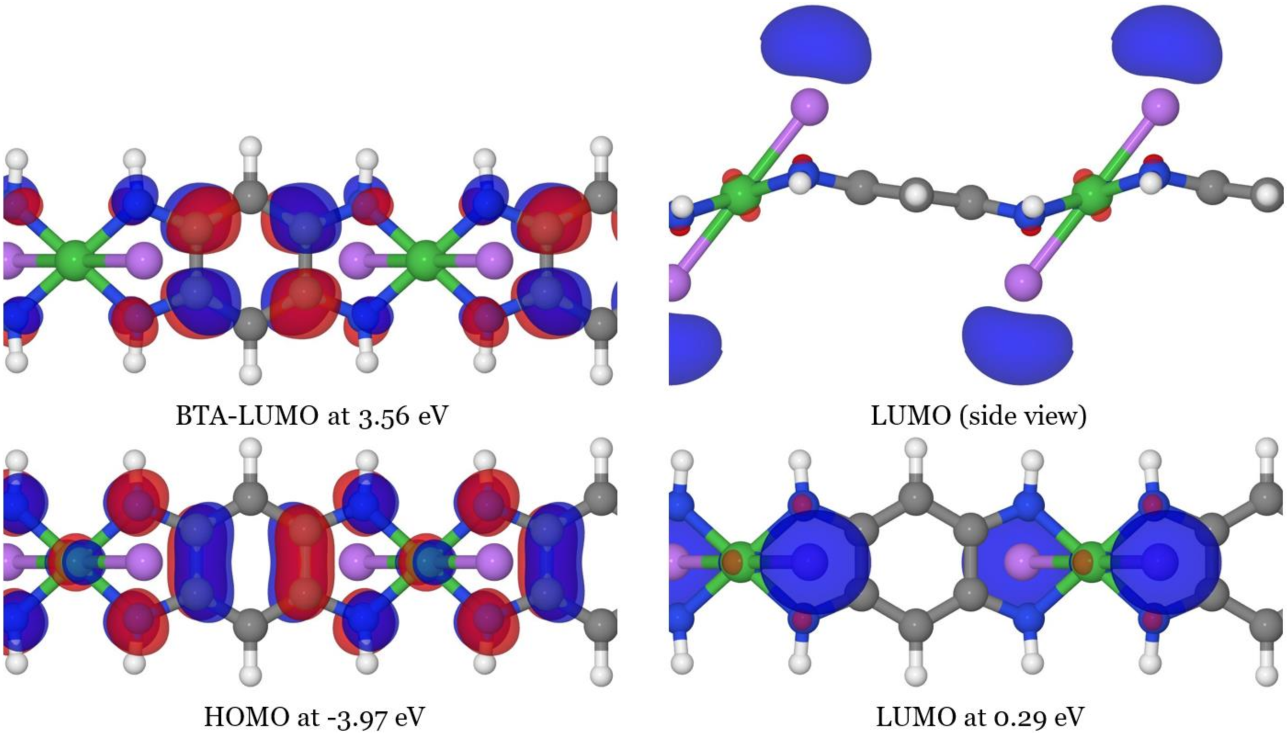

Figure S8. Frontier molecular orbitals of the isolated lithiated macromolecule (Γ-point). The BTA-LUMO is the lowest MO localized on BTA.

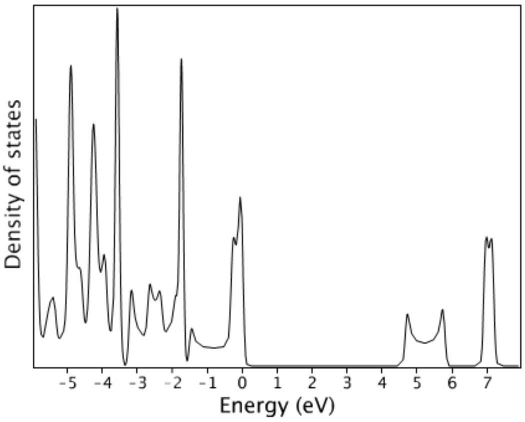

(a) polym CAM-B3LYP/p2p/a3p

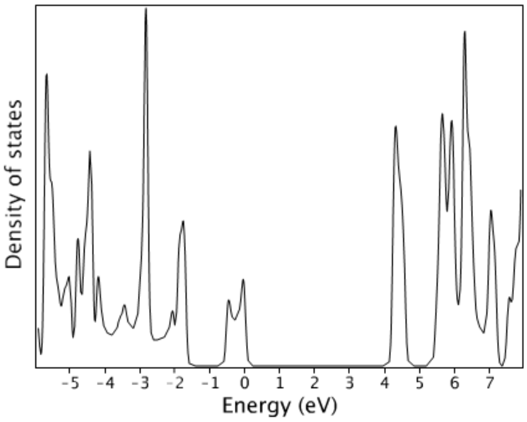

(b) polymLiLi CAM-B3LYP/p2p/a3p

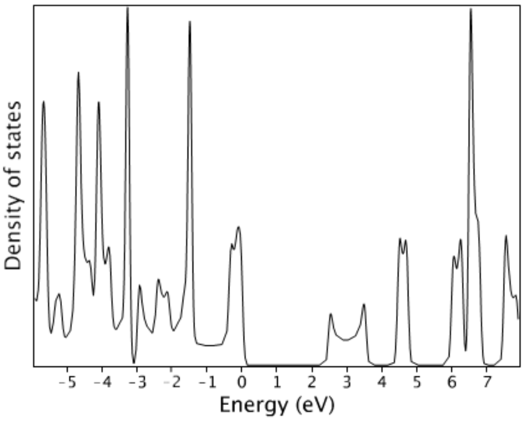

(c) polym PBE0/p2p/a3p

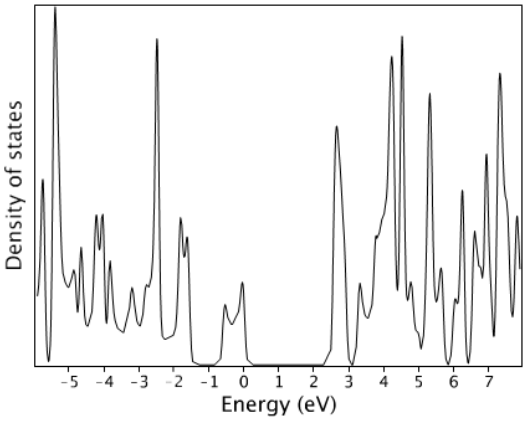

(d) polymLiLi PBE0/p2p/a3p

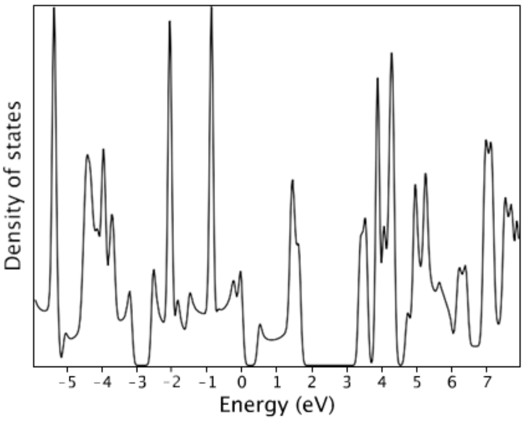

(e) polym PBE/a3p

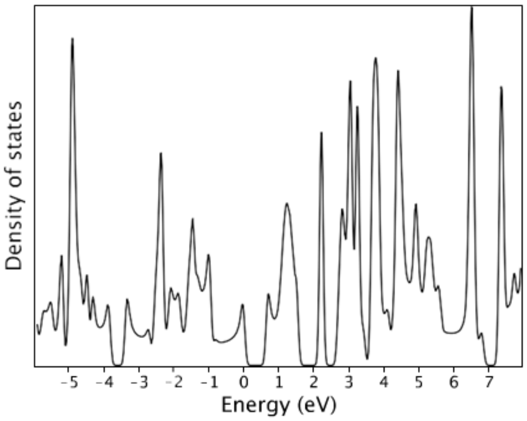

(f) polymLiLi PBE/a3p

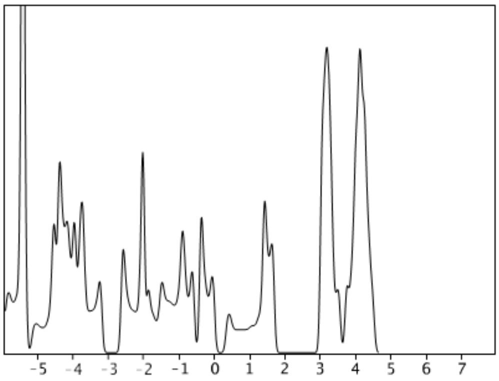

(g) polym PBE-D3/PAW600

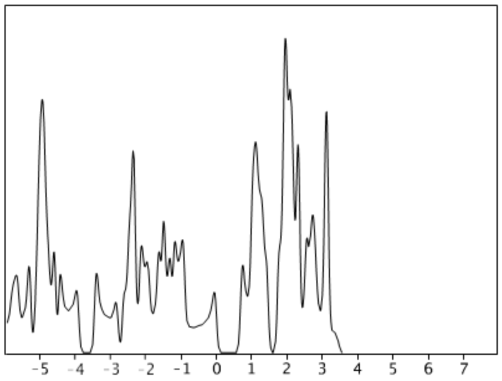

(h) polymLiLi PBE-D3/PAW600

Figure S9. Electronic density of states for NiBTA calculated using various methods.

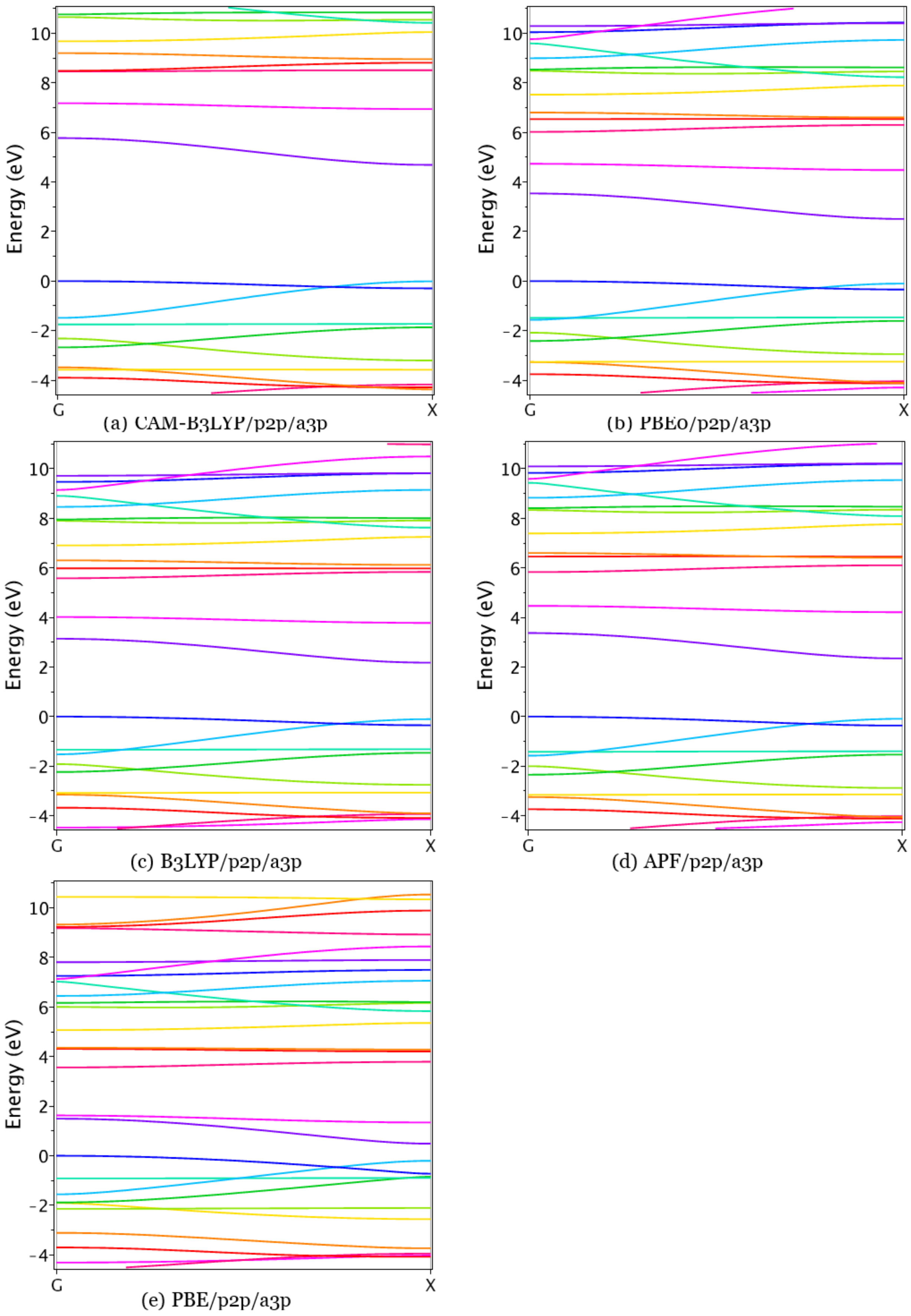

Figure S10. Electronic band structure of the standalone pristine macromolecule calculated using various methods.

Table S3. Energies of core MOs (in eV) for the standalone macromolecule in the pristine and reduced states. No wave-function convergence was achieved for potassiated polymer. The two numbers for the valence band (VB) maximum indicate the energy of the highest and the next highest MO at Γ-point, so that VBmax1 corresponds to the same-shape MO for both pristine and reduced states.

	Ni1s	N1s	C1s(σ)	VBmin	VBmax1	VBmax2
pristine	-8160.33	-391.70	-279.80(1.11)	-27.97	-6.02	
lithiated	-8159.16	-390.18	-278.37(0.43)	-26.25	-5.68	-3.97
sodiated	-8158.57	-389.54	-277.64(0.40)	-25.46	-4.94	-3.23

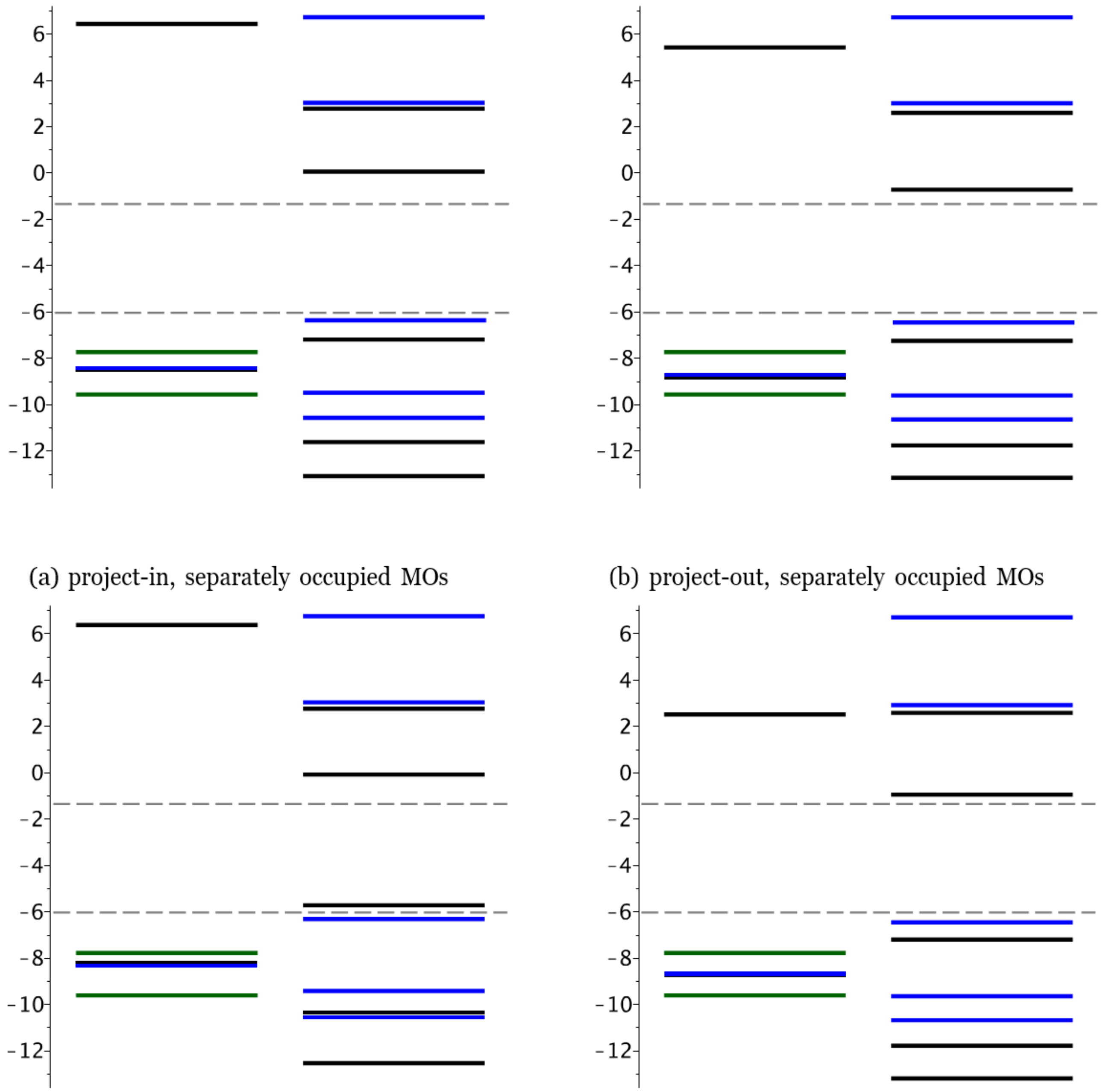

(a) project-in, separately occupied MOs

(b) project-out, separately occupied MOs

(c) project-in, all MOs at once

(d) project-out, all MOs at once

Figure S11. Comparison of LMOs obtained by variation of localization procedure. We can either localize occupied and unoccupied MOs separately or localize all MOs at once; also, we can either maximize projection onto the selected subspace ("project-in") or minimize the projection out of this subspace. The variant (d) produces the most localized LMOs, whereas the variant (a) results in the most compact tight-binding Hamiltonian. In the latter case, the LMO energies are close to energies of those MOs to which they contribute mostly. For this reason, we use the variant (a) as the default in the present work.

S4 Population analysis

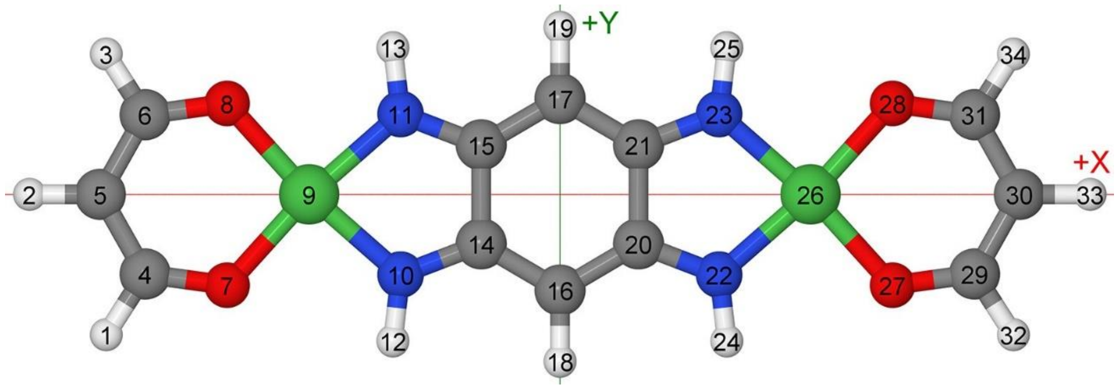

Figure S12. Orientation and atom indexing of the NiBTA low-molecular model used for population analysis. The analysis for a longer oligomer (shown in **Figure S13**, data shown in **Table S7** and **Table S8**) reveals the same features as for the short structure (**Table S4** and **Table S5**), indicating that the latter is a suitable model for analyzing qualitative trends.

Table S4. Natural atomic orbital (NAO) populations for pristine and doubly charged oligomer shown in **Figure S12**, compared also with the BTA molecule shown in **Figure S6**. Here *q* is natural atomic charge and other columns show population in each electronic subsystem. If we assume that covalently bound C atom is in electronic configuration C2*s*2p^3, triply coordinated N atom in N2*s*2p^4 (a strong σ-acceptor but also a strong π-donor), O atom in O2s^2p^4, and Ni in its present coordination is in Ni3d^{10} configuration (a weak donor in both channels), then the normal population of the π-system is 36. The missing 4 electrons are withdrawn from N and O atoms at about 0.5 electron per atom. At the same time, these atoms have excessive population in σ-system resulting in their negative charge. This π-to-σ transfer does not occur in the BTA molecule, therefore the role of transition metal is critical for the process. Indeed, the Ni-N bonds are formed from Ni sp^2d hybrids, but the population of Ni 4*sp* orbitals is very low implying extremely polar bonds. However, the total charge on Ni is not very high (+0.5 to +0.7) meaning a strong back-donation via 3d_{xz} and 3d_{yz} orbitals (π-back-bonding), whose population is close to maximum. Finally, the e_2-orbitals (Z2 and X2-Y2) remain intact (negligible depletion) as lone pairs deep in the valence band and should be electronically inert.

			pristine				dianion				BTA		
atom		mul	*q*	σ	π	LP	*q*	σ	π	LP	q	σ	π
1	H	4	+0.19	0.80			+0.19	0.81					
2	H	2	+0.25	0.75			+0.25	0.75					
4	C	4	+0.35	2.85	0.77		+0.34	2.86	0.78				
5	C	2	-0.54	3.29	1.24		-0.56	3.29	1.26				
7	O	4	-0.60	4.99	1.60		-0.62	5.02	1.59				
9	Ni	2	+0.65	1.40	4.01	3.93	+0.47	1.50	4.10	3.91			
*	H	4									+0.39	0.61	
10	N	4	-0.70	4.18	1.51		-0.86	4.07	1.78		-0.87	3.99	1.87
12	H	4	+0.41	0.59			+0.37	0.63			+0.40	0.59	
14	C	4	+0.28	2.84	0.86		+0.10	2.82	1.06		+0.12	2.81	1.05
16	C	2	-0.45	3.18	1.25		-0.39	3.18	1.20		-0.31	3.16	1.14
18	H	2	+0.24	0.76			+0.20	0.79			+0.22	0.78	
	Total		0.00	83.78	31.93	7.86	-2.00	83.84	33.92	7.82	0.00	39.89	13.96
	Model		0	80	36	8	-2	82	36	8	0	40	14
Bond lengths			NiN	CN	CC	CC*	NiN	CN	CC	CC*	CN	CC	CC*
	in Å		1.86	1.31	1.50	1.39	1.84	1.39	1.41	1.40	1.39	1.40	1.39

* Here the CC* is the bond length C14-C16 and CC is the bond length C14-C15.

Table S5. Natural atomic orbitals in details. See **Table S4** for explanations.

			pristine		dianion	
atom number	atom type	orbital	energy, eV	occupancy	energy, eV	occupancy
1	H	1s	1.94	0.80	2.30	0.81
2	H	1s	3.05	0.75	3.49	0.75
4	C	2s	-4.69	0.91	-4.29	0.91
4	C	2px	0.18	0.81	0.58	0.82
4	C	2py	-1.75	1.13	-1.33	1.13
4	C	2pz	-2.91	0.77	-2.56	0.78
5	C	2s	-4.87	0.98	-4.50	0.98
5	C	2px	-2.22	1.22	-1.79	1.22
5	C	2py	-1.89	1.09	-1.48	1.09
5	C	2pz	-3.63	1.24	-3.31	1.26
7	O	2s	-25.57	1.64	-25.01	1.64
7	O	2px	-9.69	1.53	-9.09	1.53

7	O	2py	-9.83	1.82	-9.31	1.84
7	O	2pz	-8.76	1.60	-8.25	1.59
9	Ni	3dx2y2	-10.22	1.99	-8.92	1.99
9	Ni	3dz2	-8.32	1.94	-7.08	1.92
9	Ni	3dyz	-8.89	1.97	-7.73	1.97
9	Ni	3dxz	-8.98	1.98	-7.86	1.99
9	Ni	3dxy	-4.32	0.74	-3.30	0.78
9	Ni	4s	5.48	0.35	5.30	0.40
9	Ni	4px	13.18	0.16	13.75	0.17
9	Ni	4py	13.20	0.15	13.50	0.16
9	Ni	4pz	5.73	0.07	5.88	0.14
10	N	2s	-15.28	1.35	-13.51	1.34
10	N	2px	-6.84	1.40	-4.64	1.35
10	N	2py	-6.36	1.43	-4.26	1.38
10	N	2pz	-6.58	1.51	-5.52	1.78
12	H	1s	4.96	0.59	5.82	0.63
14	C	2s	-2.47	0.82	-0.07	0.81
14	C	2px	-0.49	0.98	1.39	0.96
14	C	2py	-1.37	1.03	0.64	1.05
14	C	2pz	-2.54	0.86	-0.99	1.06
16	C	2s	-3.12	0.92	-1.75	0.92
16	C	2px	-1.03	1.06	0.46	1.08
16	C	2py	-1.76	1.21	-0.18	1.18
16	C	2pz	-3.21	1.25	-1.77	1.20
18	H	1s	2.96	0.76	3.43	0.79

Table S6. Natural atomic charges for oligomers of various length, termination, and oxidation state. Here the terminations are according to **Figure S52** with the number at the end of the oligomer name indicating the number of Ni atoms (molecOBe is the same as molecOLi but with Be instead of Li). In the potassiated molecOBe9 two K atoms are perfectly aligned with the central Ni atom by symmetry (otherwise those positions are unstable).

oligomer	state	Ni	N	NiN_4	NiN_4H_4	C_6H_2	monomer	alkali ions	total
molecO3	pristine	+0.54	-0.70	-2.25	-0.68	+0.69	+0.01		+0.01
molecO5	pristine	+0.54	-0.70	-2.25	-0.68	+0.69	+0.00		+0.00
molecO9	pristine	+0.54	-0.70	-2.25	-0.69	+0.69	+0.00		+0.00
molecOLi9	pristine	+0.54	-0.70	-2.25	-0.69	+0.69	+0.00		+0.00
molecO5	charged	+0.23	-0.89	-3.32	-1.93	-0.06	-1.99		-1.99
molecOLi9	potassiated	+0.30	-0.92	-3.36	-1.85	+0.06	-1.80	+1.80	+0.00
molecOBe9	potassiated	+0.06	-0.87	-3.42	-1.90	+0.02	-1.88	+1.87	-0.01
molecO5	difference	-0.32	-0.19	-1.07	-1.24	-0.75	-1.99		-1.99
molecOLi9	difference	-0.24	-0.22	-1.11	-1.17	-0.63	-1.80	+1.80	+0.00

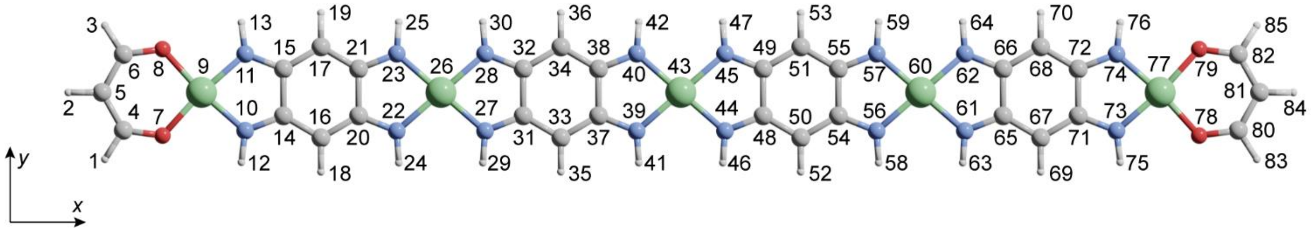

Figure S13. Orientation and atom indexing of the molecO5 used for population analysis. As seen from **Table S6**, charge distributions at central Ni and BTA blocks are the same for longer oligomers and other chain terminations indicating that length of the selected oligomer is sufficient for adequate modeling of NiBTA.

Table S7. Natural atomic charges and orbital (NAO) populations for pristine and doubly fully reduced NiBTA oligomers shown in **Figure S13**. Here *q* is natural atomic charge and other columns show population in each electronic subsystem.

		pristine				reduced (2e$^-$ per repeating unit)			
atom	mul.	*q*	*σ*	*π*	LP	*q*	*σ*	*π*	LP
1H	4	0.19	0.80			0.19	0.81		
2H	2	0.25	0.75			0.25	0.75		
4C	4	0.35	2.85	0.77		0.34	2.86	0.78	
5C	2	-0.54	3.29	1.24		-0.57	3.29	1.26	
7O	4	-0.60	4.99	1.60		-0.62	5.02	1.59	
9Ni	2	0.65	1.40	4.01	3.93	0.45	1.51	4.11	3.91
10N	4	-0.70	4.17	1.51		-0.87	4.06	1.79	
12H	4	0.40	0.59			0.37	0.63		
14C	4	0.28	2.84	0.86		0.08	2.82	1.08	
16C	4	-0.45	3.18	1.25		-0.4	3.18	1.21	
18H	4	0.24	0.76			0.20	0.80		
20C	4	0.28	2.84	0.86		0.11	2.83	1.04	
22N	4	-0.70	4.18	1.50		-0.88	4.10	1.76	
24H	4	0.39	0.61			0.35	0.65		
26Ni	2	0.54	1.51	4.01	3.93	0.23	1.69	4.16	3.89
27N	4	-0.70	4.18	1.50		-0.89	4.09	1.77	
29H	4	0.39	0.61			0.35	0.65		
31C	4	0.28	2.84	0.86		0.09	2.83	1.06	
33C	4	-0.45	3.18	1.25		-0.41	3.17	1.22	
35H	4	0.23	0.77			0.19	0.81		
37C	4	0.28	2.84	0.86		0.10	2.83	1.06	
39N	4	-0.70	4.18	1.50		-0.89	4.10	1.77	
41H	4	0.39	0.61			0.35	0.65		
43Ni	1	0.54	1.51	4.01	3.93	0.23	1.70	4.16	3.89

Table S8. Natural atomic orbitals for pristine and reduced NiBTA models shown in **Figure S13**.

			pristine		reduced (2e$^-$ per repeating unit)	
atom number	atom type	orbital	energy, eV	occupancy	energy, eV	occupancy
1	H	1s	1.94	0.80	2.35	0.81
2	H	1s	3.05	0.75	3.54	0.75
4	C	2s	-4.68	0.91	-4.20	0.91
4	C	2px	0.19	0.81	0.67	0.82
4	C	2py	-1.74	1.13	-1.24	1.13
4	C	2pz	-2.90	0.77	-2.48	0.78
5	C	2s	-4.86	0.98	-4.42	0.98
5	C	2px	-2.21	1.22	-1.70	1.22
5	C	2py	-1.88	1.09	-1.40	1.09
5	C	2pz	-3.62	1.24	-3.24	1.26
7	O	2s	-25.56	1.64	-24.9	1.64
7	O	2px	-9.68	1.53	-8.99	1.53
7	O	2py	-9.81	1.82	-9.20	1.84
7	O	2pz	-8.75	1.60	-8.15	1.59
9	Ni	3dx2y2	-10.2	1.99	-8.76	1.99
9	Ni	3dz2	-8.30	1.94	-6.92	1.92
9	Ni	3dyz	-8.87	1.97	-7.57	1.97
9	Ni	3dxz	-8.96	1.98	-7.71	1.99
9	Ni	3dxy	-4.30	0.74	-3.16	0.79
9	Ni	4s	5.50	0.35	5.35	0.40
9	Ni	4px	13.20	0.16	13.86	0.17
9	Ni	4py	13.22	0.15	13.59	0.16
9	Ni	4pz	5.75	0.07	5.96	0.15
10	N	2s	-15.25	1.35	-13.32	1.34
10	N	2px	-6.80	1.40	-4.41	1.35
10	N	2py	-6.33	1.43	-4.03	1.38
10	N	2pz	-6.56	1.51	-5.34	1.79
12	H	1s	4.98	0.59	5.93	0.63
14	C	2s	-2.42	0.82	0.26	0.80
14	C	2px	-0.46	0.98	1.71	0.96
14	C	2py	-1.34	1.03	0.95	1.05
14	C	2pz	-2.51	0.86	-0.72	1.08
16	C	2s	-3.10	0.92	-1.47	0.92
16	C	2px	-1.01	1.06	0.76	1.08
16	C	2py	-1.74	1.20	0.16	1.18
16	C	2pz	-3.20	1.25	-1.52	1.21
18	H	1s	2.91	0.76	3.54	0.80
20	C	2s	-2.44	0.82	0.25	0.81
20	C	2px	-0.43	0.98	1.82	0.97
20	C	2py	-1.32	1.03	1.08	1.05
20	C	2pz	-2.50	0.86	-0.56	1.04
22	N	2s	-15.21	1.35	-12.75	1.35

22	N	2px	-6.76	1.42	-3.83	1.38
22	N	2py	-6.21	1.42	-3.36	1.38
22	N	2pz	-6.49	1.50	-4.75	1.76
24	H	1s	4.63	0.61	5.94	0.65
26	Ni	3dx2y2	-9.74	1.98	-7.29	1.98
26	Ni	3dz2	-7.94	1.95	-5.55	1.91
26	Ni	3dyz	-8.52	1.99	-6.33	1.99
26	Ni	3dxz	-8.49	1.95	-6.44	1.99
26	Ni	3dxy	-4.14	0.79	-1.90	0.83
26	Ni	4s	6.64	0.37	5.99	0.46
26	Ni	4px	15.47	0.18	16.47	0.21
26	Ni	4py	12.97	0.17	13.97	0.20
26	Ni	4pz	6.24	0.06	6.64	0.18
27	N	2s	-15.19	1.35	-12.65	1.35
27	N	2px	-6.74	1.41	-3.69	1.37
27	N	2py	-6.19	1.42	-3.23	1.37
27	N	2pz	-6.49	1.50	-4.67	1.77
29	H	1s	4.63	0.61	5.98	0.65
31	C	2s	-2.40	0.82	0.51	0.81
31	C	2px	-0.42	0.98	2.05	0.97
31	C	2py	-1.30	1.03	1.30	1.05
31	C	2pz	-2.48	0.86	-0.38	1.06
33	C	2s	-3.10	0.92	-1.27	0.92
33	C	2px	-1.01	1.06	0.98	1.08
33	C	2py	-1.73	1.20	0.40	1.17
33	C	2pz	-3.20	1.25	-1.34	1.22
35	H	1s	2.85	0.77	3.57	0.81
37	C	2s	-2.40	0.82	0.50	0.81
37	C	2px	-0.42	0.98	2.05	0.97
37	C	2py	-1.30	1.03	1.31	1.05
37	C	2pz	-2.48	0.86	-0.37	1.06
39	N	2s	-15.19	1.35	-12.62	1.35
39	N	2px	-6.74	1.41	-3.66	1.37
39	N	2py	-6.19	1.42	-3.19	1.37
39	N	2pz	-6.48	1.50	-4.64	1.77
41	H	1s	4.63	0.61	5.99	0.65
43	Ni	3dx2y2	-9.73	1.98	-7.21	1.98
43	Ni	3dz2	-7.93	1.95	-5.48	1.91
43	Ni	3dyz	-8.51	1.99	-6.26	1.99
43	Ni	3dxz	-8.49	1.95	-6.37	1.99
43	Ni	3dxy	-4.13	0.79	-1.83	0.83
43	Ni	4s	6.65	0.37	5.97	0.46
43	Ni	4px	15.47	0.18	16.52	0.21
43	Ni	4py	12.98	0.17	14.01	0.20
43	Ni	4pz	6.24	0.06	6.67	0.18

S5 Contributions from carbon black to the capacity

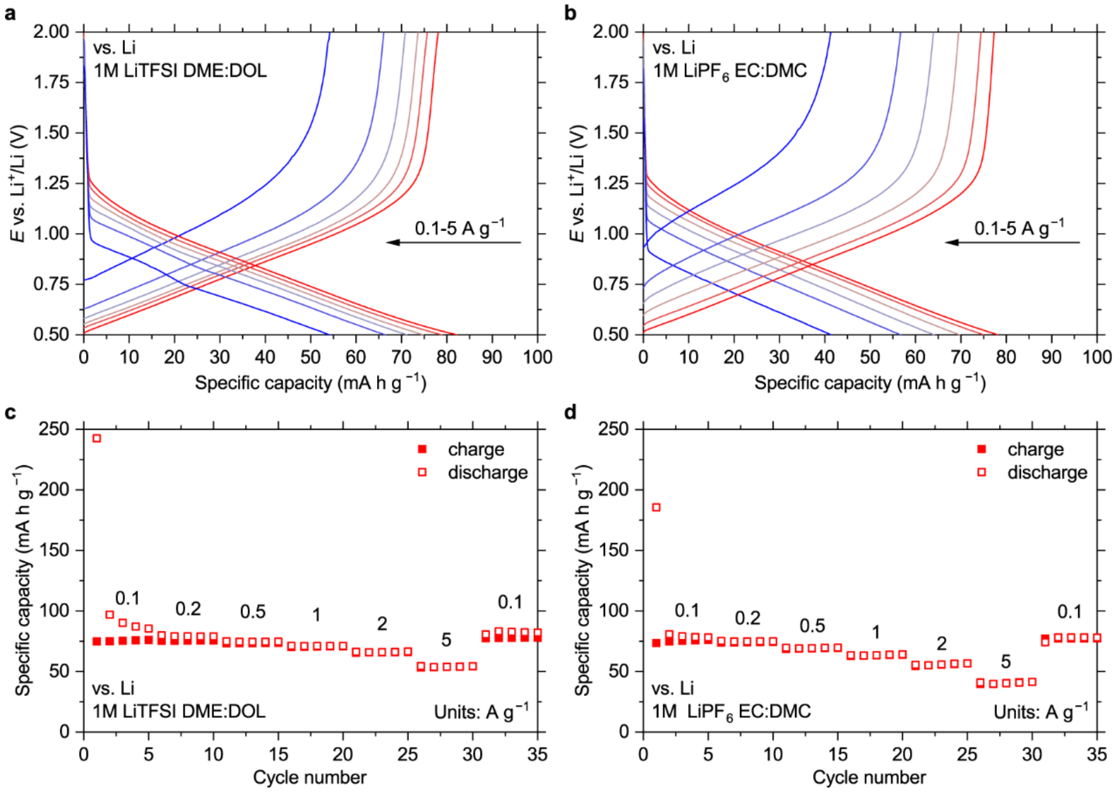

Figure S14. Electrochemistry of Super P in lithium-based cells in the 0.5–2.0 V vs. Li^+/Li potential range.Charge-discharge curves with the ether-based (a) and carbonate-based (b) electrolytes at 0.1, 0.2, 0.5, 1, 2 and 5 A g^{-1}; dependencies of charge and discharge capacities on the cycle number at varying current densities for the cells with ether-based (c) and carbonate-based (d) electrolytes.

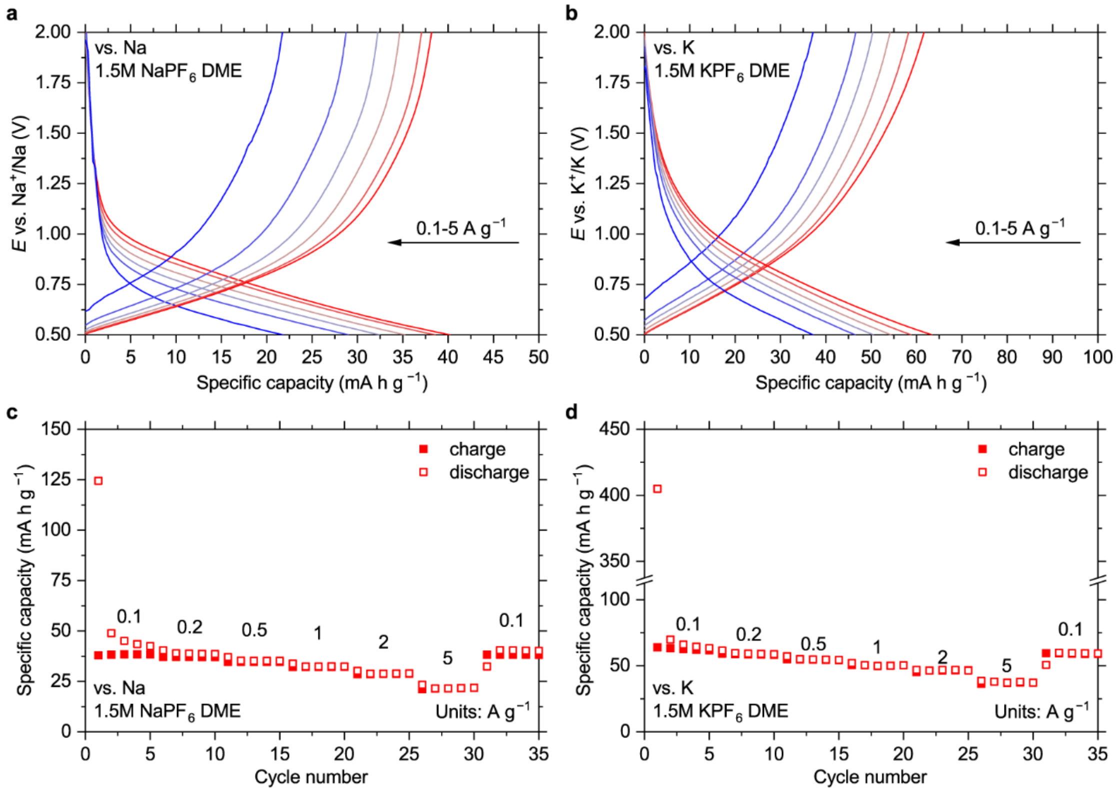

Figure S15. Electrochemistry of Super P in sodium- and potassium-based cells in the 0.5–2.0 V vs. M^+/M potential ranges.

Charge-discharge curves in sodium-based (a) and potassium-based cells (b) at 0.1, 0.2, 0.5, 1, 2 and 5 A g^{-1}; dependencies of charge and discharge capacities on the cycle number at varying current densities for sodium-based (c) and potassium-based cells (d).

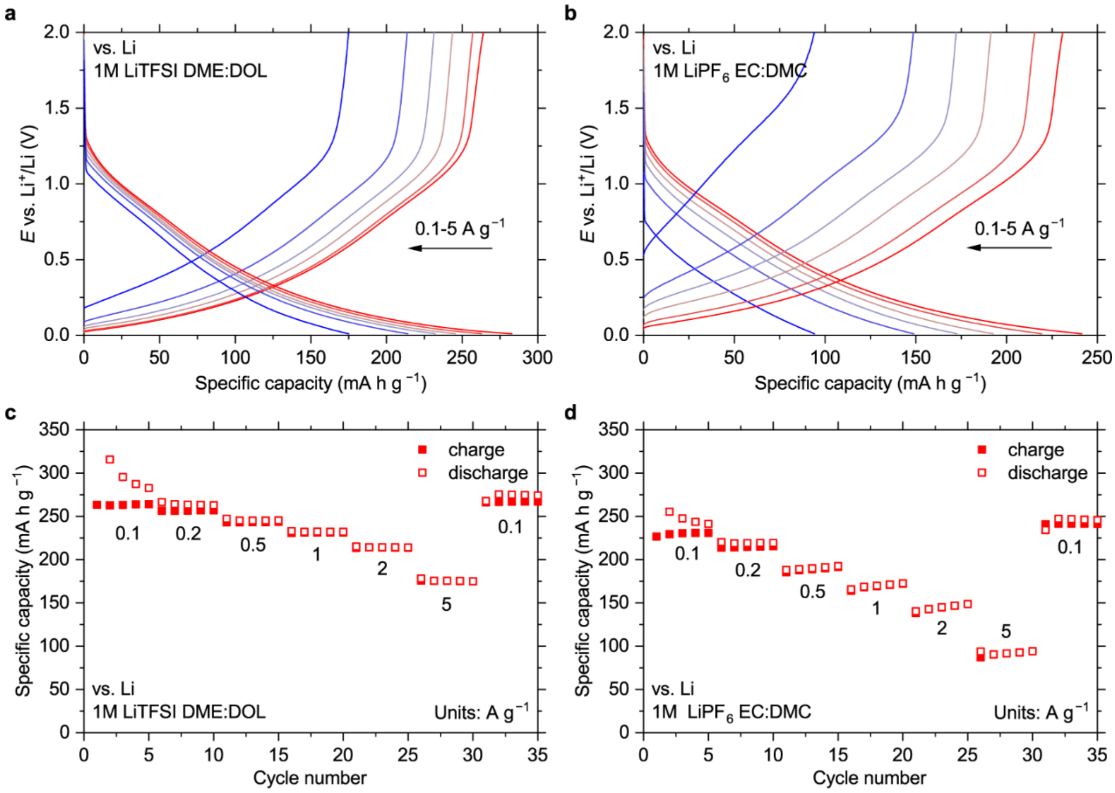

Figure S16. Electrochemistry of Super P in lithium-based cells in the 0.01–2.0 V vs. Li^+/Li potential range.Charge-discharge curves with the ether-based (a) and carbonate-based (b) electrolytes at 0.1, 0.2, 0.5, 1, 2 and 5 A g^{-1}; dependencies of charge and discharge capacities on the cycle number at varying current densities for the cells with ether-based (c) and carbonate-based (d) electrolytes.

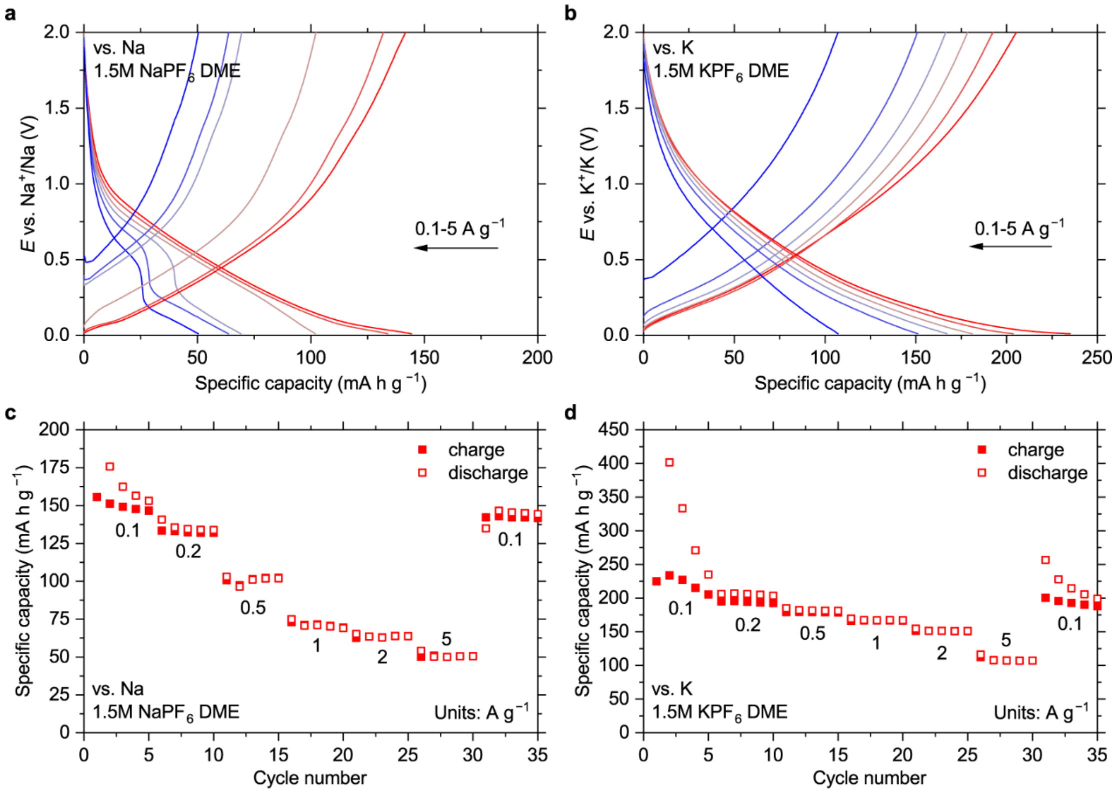

Figure S17. Electrochemistry of Super P in sodium- and potassium-based cells in the 0.01–2.0 V vs. M^+/M potential ranges. Charge-discharge curves in sodium- (a) and potassium-based cells (b) at 0.1, 0.2, 0.5, 1, 2 and 5 A g^{-1}; dependencies of charge and discharge capacities on the cycle number at varying current densities for sodium-based (c) and potassium-based cells (d).

S6 Summary for the rate capabilities of NiBTA

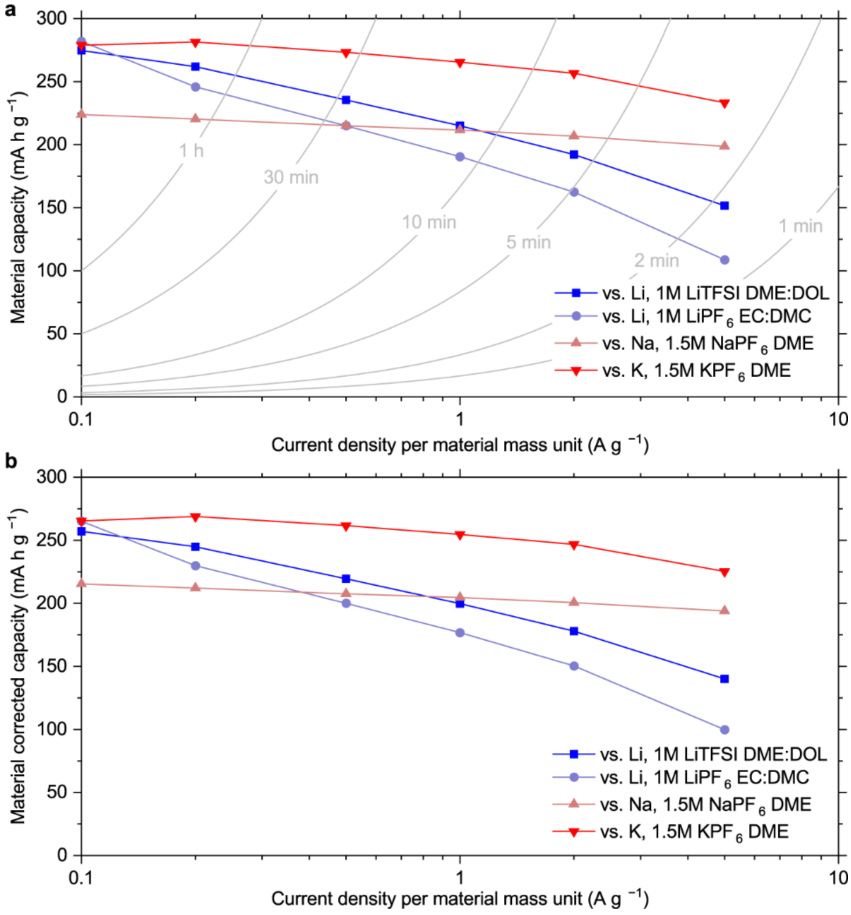

Figure S18. Capacity vs. current density calculated per NiBTA mass for various types of cells (a) and NiBTA capacity after subtracting the contribution from Super P (b). The cells were cycled in in the 0.5–2.0 V vs. M^+/M potential ranges.

S7 Rate capabilities of NiBTA in Li-based cells with different electrolytes

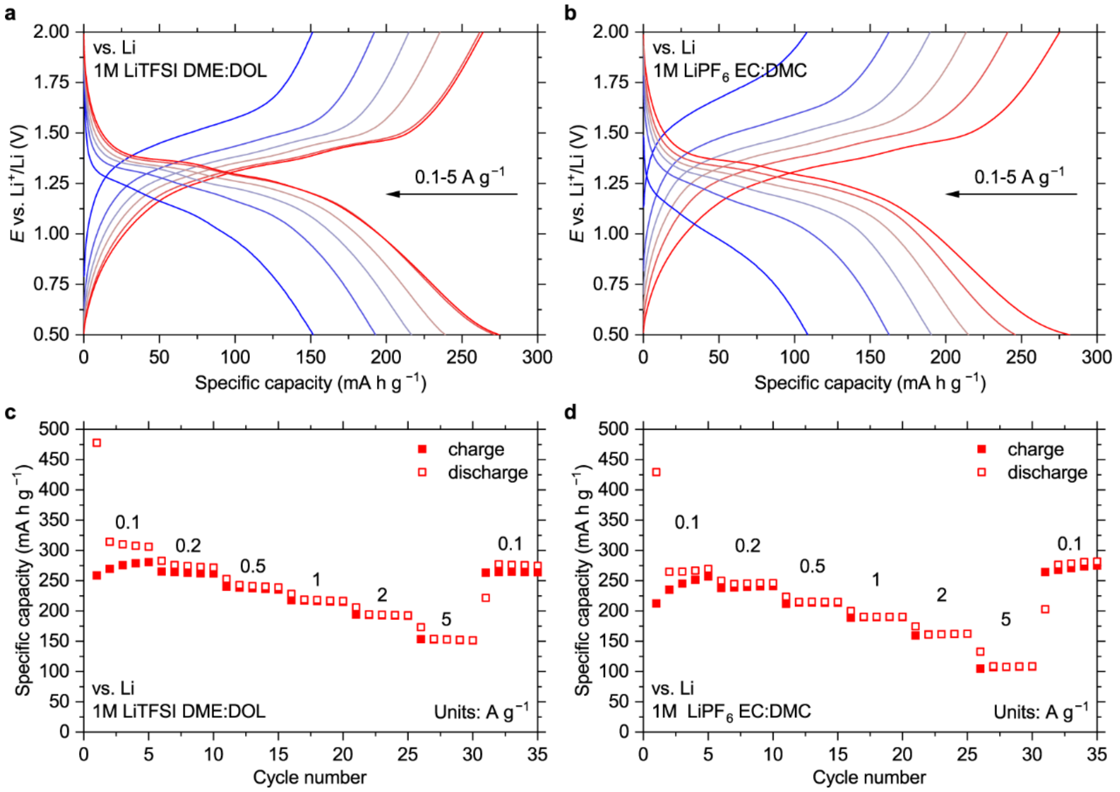

Figure S19. Electrochemistry of NiBTA in Li-based cells with different electrolytes. Charge-discharge curves of with the ether-based (a) and carbonate-based (b) electrolytes at 0.1, 0.2, 0.5, 1, 2 and 5 A g^{-1}; dependencies of charge and discharge capacities on the cycle number at varying current densities for the cells with ether-based (c) and carbonate-based (d) electrolytes. The cells were cycled in in the 0.5–2.0 V vs. Li$^+$/Li potential range.

S8 Analysis of cyclic voltammetry data for NiBTA

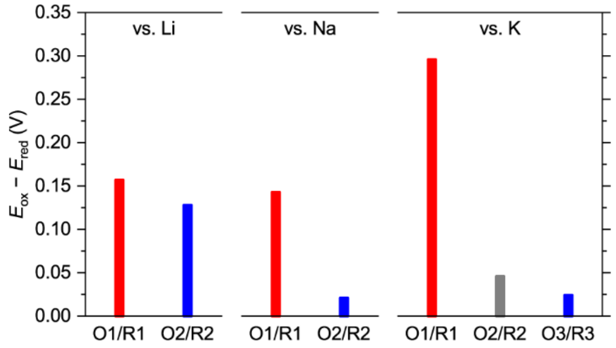

Figure S20. Potential differences between oxidation and reduction CV peaks at the scan rate of 0.2 mV s^{-1}. The cells were cycled in in the 0.5–2.0 V vs. M^{+}/M potential ranges.

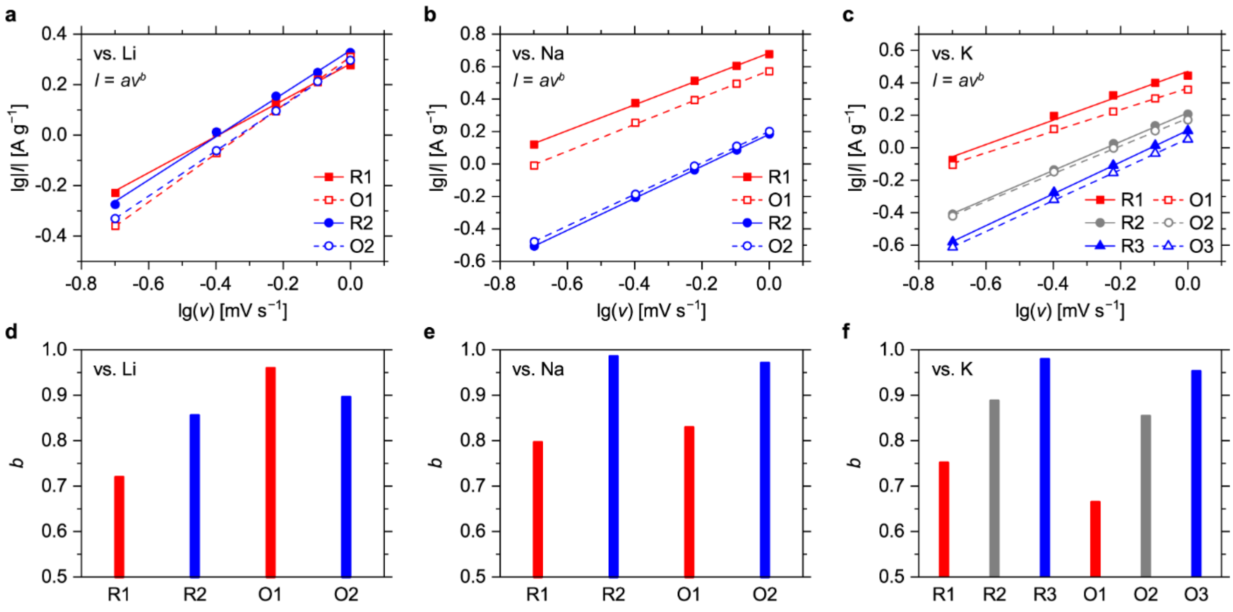

Figure S21. *I*-*v* analysis for NiBTA. Dependencies of lg(I) vs. lg(v) for the CV peaks (a-c); b values estimated from the linear fit (lg(I) = b*lg(v) + lg(a)) (d-f). Data for the Li-, Na- and K-based cells are shown in (a, d), (b, e) and (c, f), respectively. CVs were measured in the 0.5–2.0 V vs. M^{+}/M potential ranges at the scan rates of 0.2, 0.4, 0.6, 0.8 and 1 mV s^{-1}.

S9 Simulated crystal structures and XRD studies

Table S9. Types of NiBTA structures considered for simulations. Here *Z* is number of monomers per unit cell. The "shift" is a mutual shift of two polymers along each other; they are given in fraction of the monomer length for the nearest polymers along *z*- and *y*-directions respectively ('1/4' shifts are approximate). Subscript *x* in symmetry group means (*a*, *b*, *c*) → (*c*, *a*, *b*) transformation from the default International Tables for Crystallography settings.

name	symmetry	*Z*	shifts		description
Herringbone type crystals, see **Figure S22**a					
cryst-h	P-1	2	0	any	low-symmetry herringbone ('h')
cryst-ha	$Pnnm_x$	2	0	1/2	
cryst-hb	$Pbam_x$	2	0	0	
cryst-hc	P21/n	2	0	1/4	
cryst-ho	–	4	1/2	1/4	'hc' doubled in *z* and kept orthorhombic
Channeled crystals, see **Figure S22**b					
cryst-s	P1121/n	4	±1/4	1/2	kept orthorhombic, square pattern ('s')
π-Stack type crystals, see **Figure S22**c, d					
cryst-wa	Immm	2	0	1/2	brickwork pattern ('w')
cryst-wb	Ammm	2	0	0	
cryst-pa	Fmmm	4	1/2	1/2	in-plane orderding ('p')
cryst-pb	Bmmm	2	1/2	0	
Isolated *π*-stacks					
stack-w	cmmm	2	1/2		brickwork pattern ('w')
stack-wx	c2/m11	2	1/2		tilted polymers
stack-p	pmmm	1	0		
stack-px	p-1	1	any		
Isolated macromolecules					
polym	pmmm	1			
polymKK	$p2/m11_x$	1			alkali ions distort the planarity

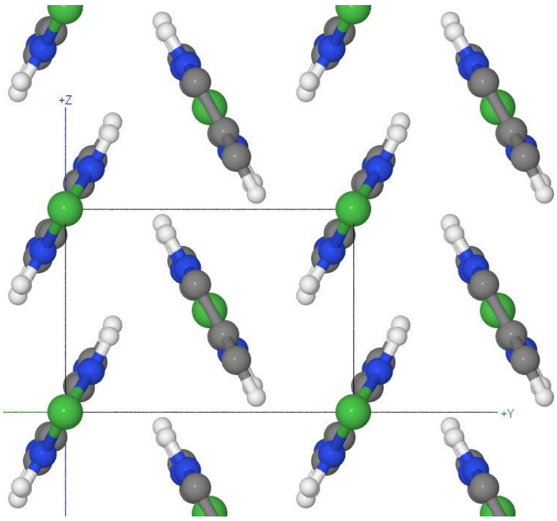

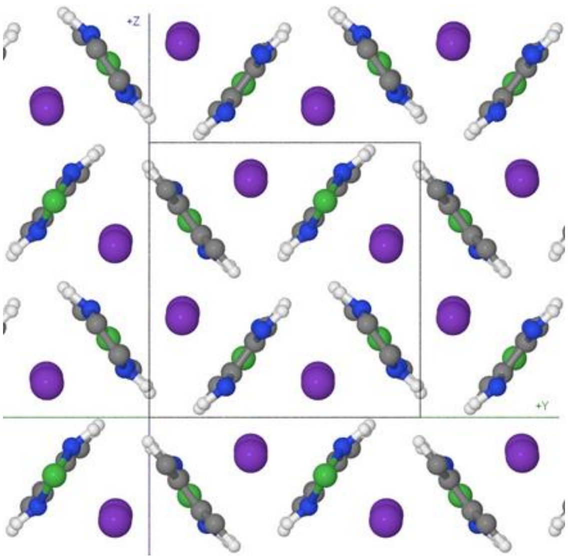

(a) herringbone (cryst-hc)

(b) channeled (crystKK-s)

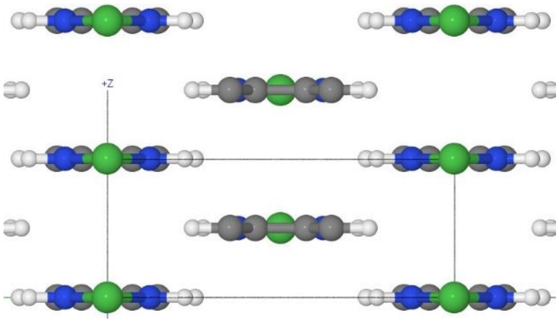

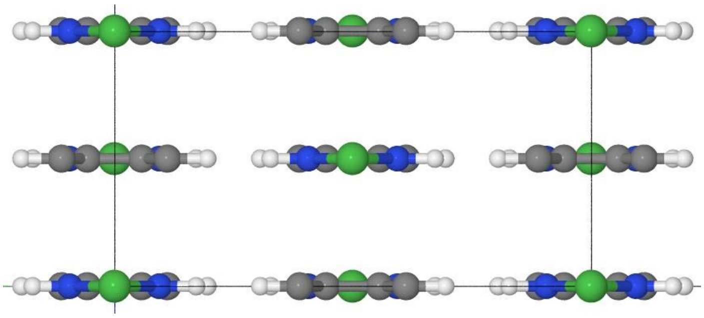

(c) brickwork (cryst-wa)

(d) in-plane (cryst-pa)

Figure S22. Different types of simulated crystal structures.

Table S10. Structures predicted by PBE-D3/PAW600 method. Here *D* is number of translation vectors, *Z* is number of monomers per unit cell, *a* is the monomer length and Δ*a* is its difference with respect to the isolated pristine macromolecule (7.674 Å), *b* and *c* are lengths of translation vectors perpendicular to the polymer with *c* being the *π*-stacking direction, c_x and c_z are along-polymer and perpendicular projections of the vector *c*, *ϕ* is the polymer setting angle relative to *ab* plane, *V* is the volume per atom not counting alkaline atoms, *E* is the total energy per monomer relative to the lowest energy polymorph. The stability is tested by 10 ps MD, listed is the number of polymers in the supercell ('x2' means double cell in the polymer direction) followed by the polymorph to which the original structure transforms. The last column shows the consistency of the predicted stable structures with experimental XRD data: '+' means consistent, '–' means not expected to be observed or is not observed, '?' indicates some inconsistencies. Multiple '+' for herringbone structures are due to the sliding of polymers along each other at room temperature.

name	*D*	*Z*	Δ*a* mÅ	*b* Å	c_z Å	c_x/a	*ϕ* deg	*V* Å^3	*E* meV	stability	exp
Pristine NiBTA											
cryst-ho	3	4	-22	7.46	10.41	0	-60.6	8.74	0	2	+
cryst-hc	3	2	-23	7.43	5.22	-0.50	-60.6	8.73	1	2, 8x2 slides	+
cryst-hb	3	2	-23	7.42	5.40	0	-58.9	9.03	53	2	+
cryst-ha	3	2	-17	7.47	5.34	0	-60.5	8.97	107	2 hb	
cryst-wa	3	2	-6	10.04	4.00	0	0	9.05	203	2 h	
cryst-wb	3	2	-23	10.35	4.35	0	0	10.14	447	2 h	
cryst-pa	3	4	+3	13.03	6.95	0	0	10.23	654	4 h	
stack-wx	2	2	-1		7.28	0	27.2		725		
stack-px	2	1	-3		3.65	0.16	27.3		726		
cryst-pb	3	2	-8	7.01	6.95	0	0	10.99	727		
stack-w	2	2	-6		7.00	0	0		865		
stack-p	2	1	-8		3.51	0	0		891		
polym	1	1	0				0		1192		
With two Li atoms per Ni											
crystLiLi-wa	3	2	200	10.37	3.77	0	0	9.06	0	4	–
crystLiLi-ho	3	4	81	7.13	11.13	0	-63.9	9.04	21	4	+
crystLiLi-h	3	2	104	7.36	5.46	-0.52	-66.4	9.06	30		+
crystLiLi-s	3	4	110	10.53	7.65	0	-39.3	9.22	44	4	–
crystLiLi-hc	3	2	96	7.89	5.09	-0.50	-63.9	9.17	262	2	
stackLiLi-p	2	1	203		3.79	0	0		774		
stackLiLi-w	2	2	184		7.71	0	0		1014		
polymLiLi	1	1	77				0		2902		
With two Na atoms per Ni											
crystNaNa-h	3	2	129	8.25	5.22	-0.52	-65.2	9.85	0		?
crystNaNa-s	3	4	132	10.24	8.72	0	-43.7	10.25	25	4	?
crystNaNa-ho	3	4	127	8.27	10.51	0	-68.0	9.97	60	4	?
crystNaNa-wa	3	2	186	10.38	4.46	0	0	10.71	186	4	–
stackNaNa-p	2	1	184		4.52	0	0		899		
stackNaNa-w	2	2	181		9.20	0	0		1042		
polymNaNa	1	1	80				0		3115		
With two K atoms per Ni											
crystKK-s	3	4	147	10.01	10.18	0	-51.8	11.73	0	16, 16x2 1ps	?
crystKK-h	3	2	144	9.08	5.65	-0.50	-71.0	11.72	178		–
crystKK-ho	3	4	135	9.05	11.31	0	-74.8	11.75	189	4 slides	
crystKK-wa	3	2	171	10.40	5.25	0	0	12.61	402	2, 4 s	
crystKK-hc	3	2	131	10.79	5.37	-0.39	-65.2	13.31	720	2 ?	

stackKK-p	2	1	174	5.32	0	0	957
stackKK-w	2	2	171	10.66	0	0	962
polymKK	1	1	106			0	2770

Table S11. The most intensive reflections in simulated powder diffraction patterns for predicted structures. Here *I* is relative intensity, *d* is inter-plane spacing. For each (hkl) and 2*θ* only the most intense reflection is listed. Reflections with nonzero 'h' are largely ignored because they are washed out by thermal motion of polymers along each other. Unstable structures are marked with asterisk.

		pristine			lithiated			sodiated			potassiated		
(hkl)	description	*I*	*d*Å	2*θ*	*I*	*d*Å	2*θ*	*I*	*d*Å	2*θ*	*I*	*d*Å	2*θ*
Herringbone packing:		cryst-hc			crystLiLi-h			crystNaNa-h			crystKK-h		
(011)	interpolymer	100	4.3	21	100	4.4	20	51	4.5	20	23	5.0	18
$(0\bar{1}1)$	interpolymer		=(011)		73	4.3	21	100	4.3	21	100	4.6	19
(020)	interpolymer	58	3.7	24	66	3.6	25	58	4.1	22	34	4.5	20
(021)	unclear	33	3.0	29	24	3.0	29						
$(10\bar{1})$	Ni square layers	14	6.2	14	18	6.3	14	26	6.3	14	16	6.4	14
(110)	Ni triangular layers	14	4.8	19									
Crystals with channels:					crystLiLi-s			crystNaNa-s			crystKK-s		
(002)	interpolymer				27	3.8	23	80	4.4	20	100	5.1	17
(021)	interpolymer				100	4.3	20	100	4.4	20	63	4.5	20
(012)	unclear				42	3.6	25						
(031)	unclear							37	3.2	28			
(032)	polymer plane (K)										64	2.8	32
(011)	channels (K)										41	7.1	12
π-stacked packing:		*cryst-wa			crystLiLi-wa			crystNaNa-wa			*crystKK-wa		
(011)	Ni triangular layers	100	3.7	24	100	3.5	25	89	4.1	22	38	4.7	19
(020)	across H-H interlayer	22	5.0	18	48	5.2	17	100	5.2	17	100	5.2	17
(110)	Ni layers	16	6.1	15	24	6.3	14	37	6.3	14			
(002)	half π-stacking	9	2.0	45	10	1.9	48	30	2.2	40	43	2.6	34

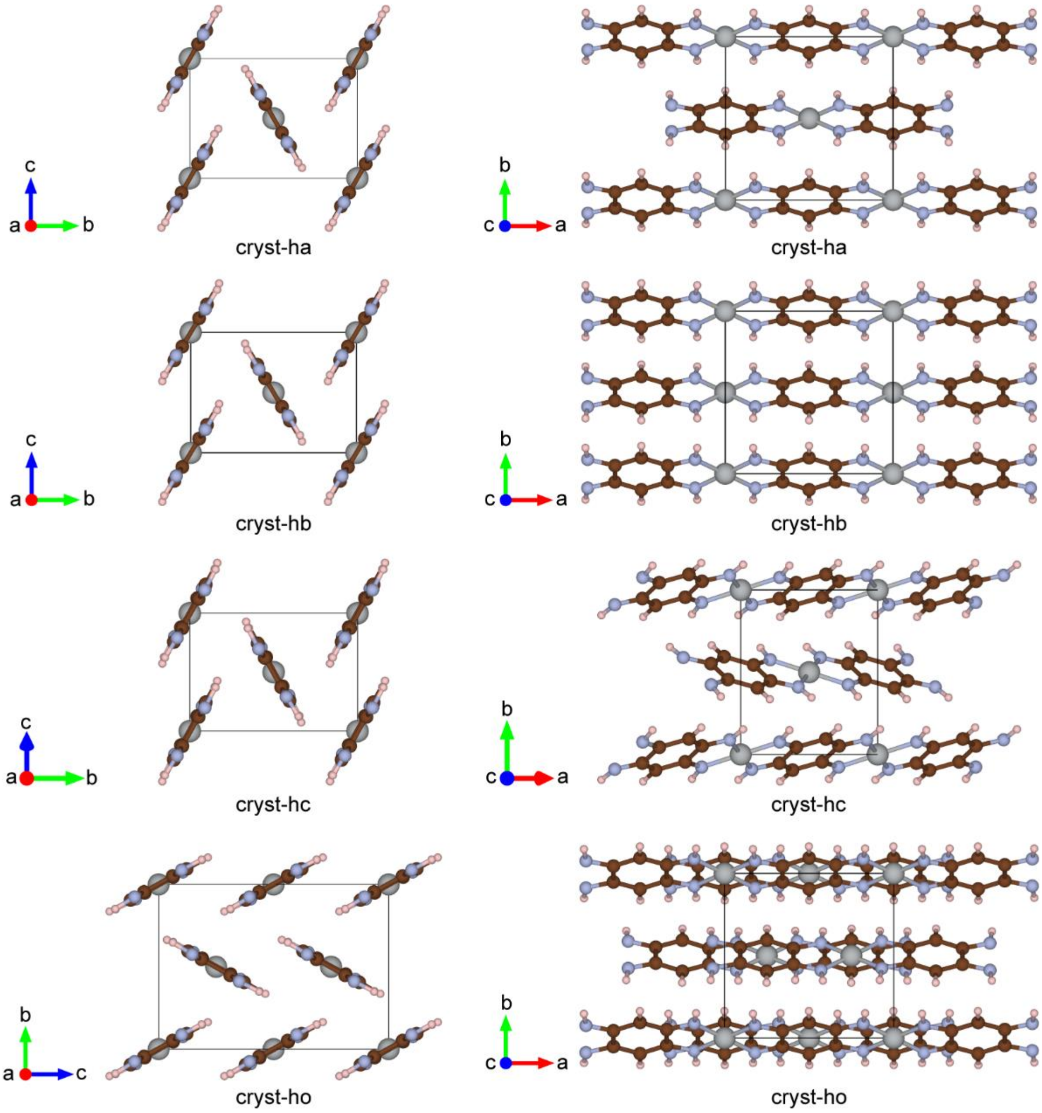

Figure S23. Simulated crystal structures of NiBTA that are most energetically favorable (herringbone-type structures).

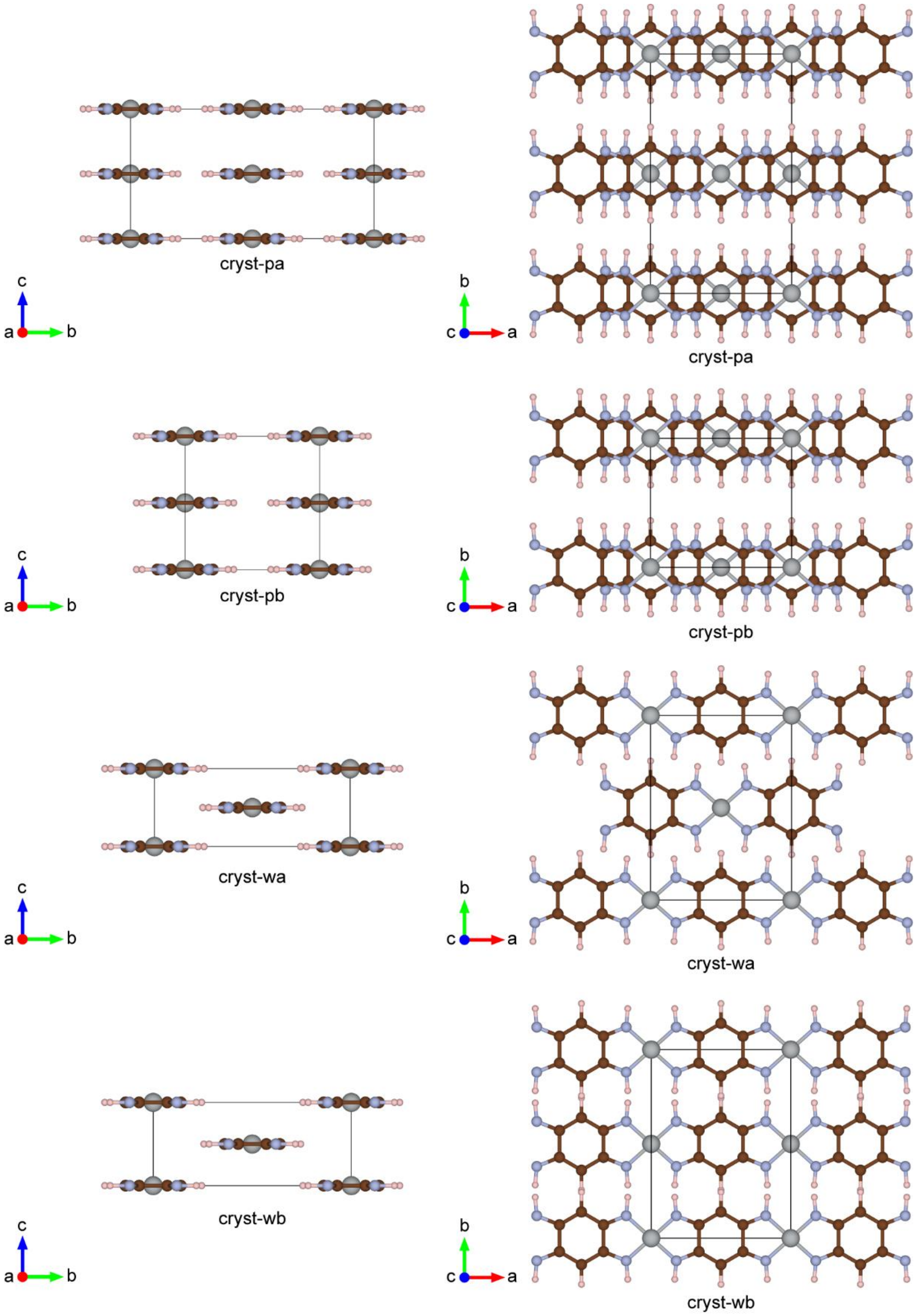

Figure S24. Simulated crystal structures of NiBTA that are less energetically favorable (brickwork and in-plane structures).

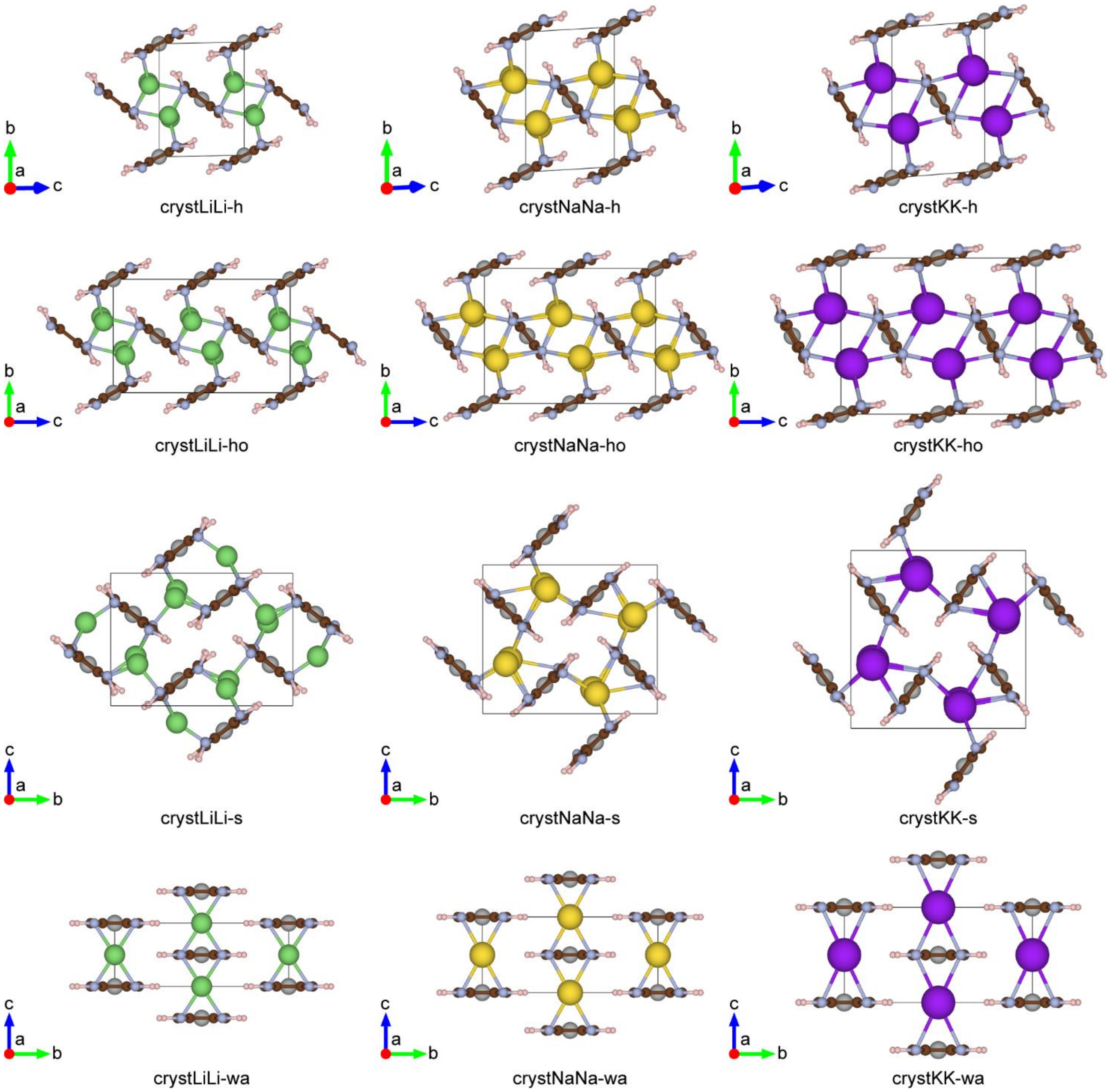

Figure S25. Various simulated crystal structures of fully lithiated/sodiated/potassiated derivatives of NiBTA.

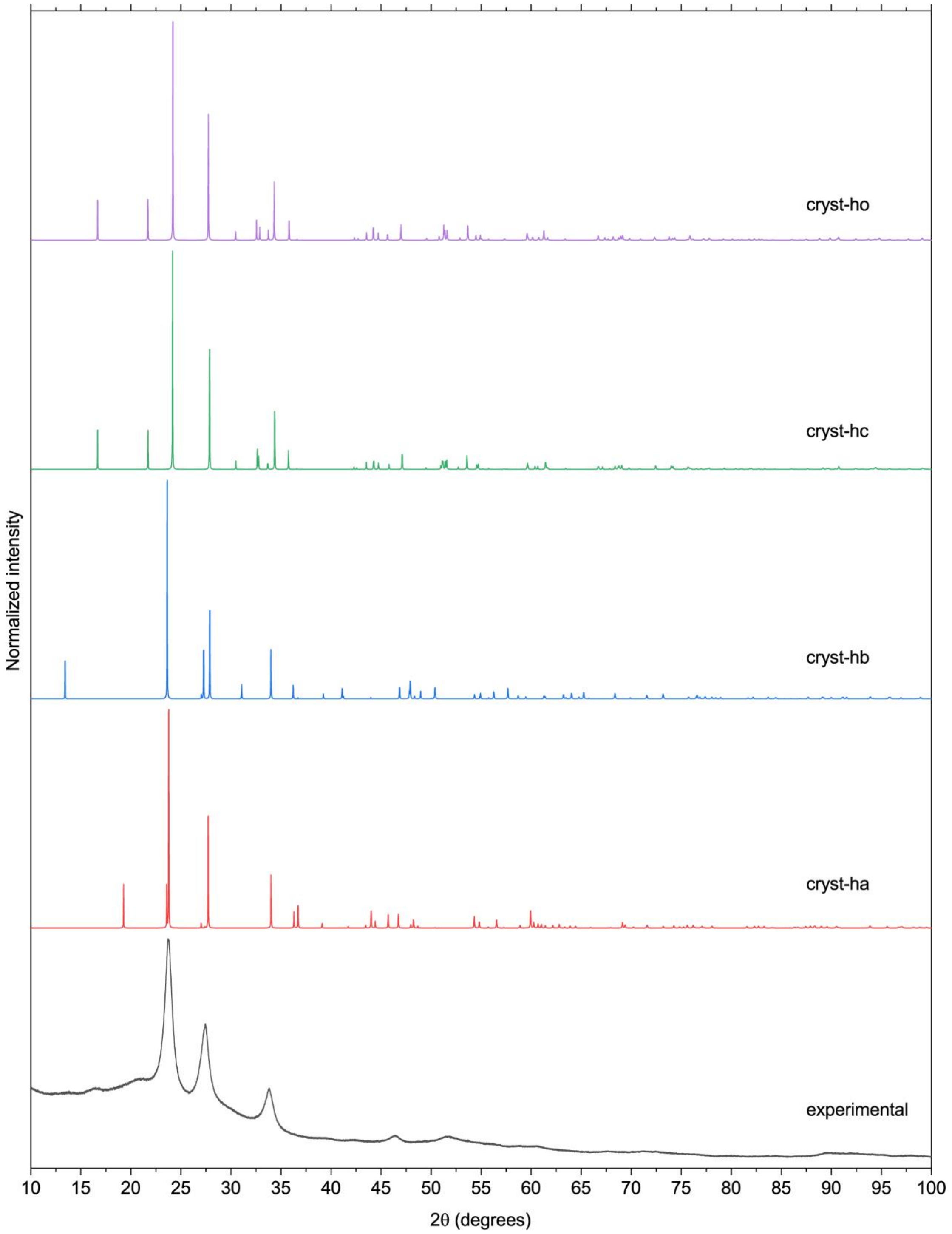

Figure S26. XRD pattern of NiBTA compared to XRD patterns of herringbone-type simulated structures (CoKα radiation, λ = 1.78892 Å).

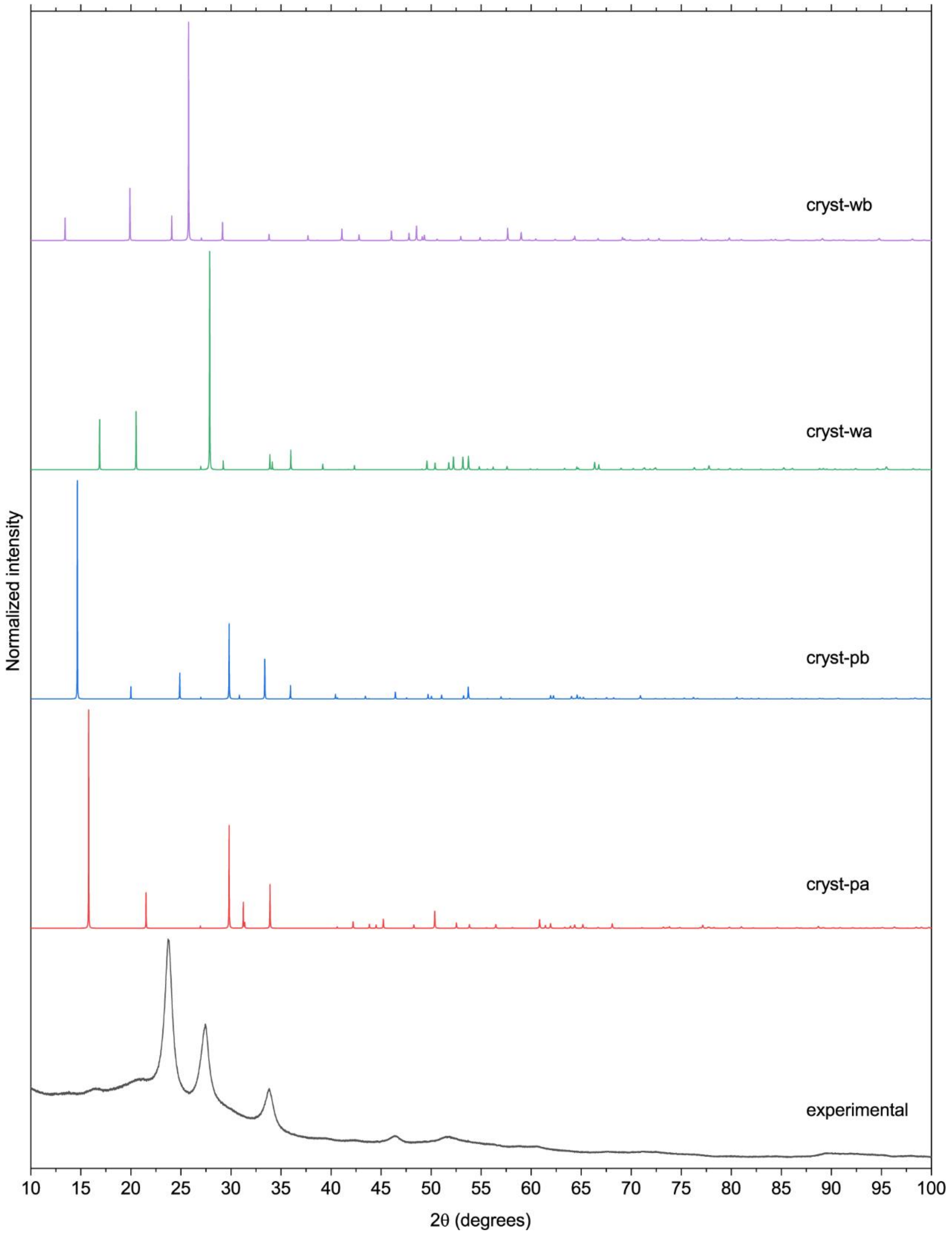

Figure S27. XRD pattern of NiBTA compared to XRD patterns of stacked-type simulated structures (CoKα radiation, λ = 1.78892 Å).

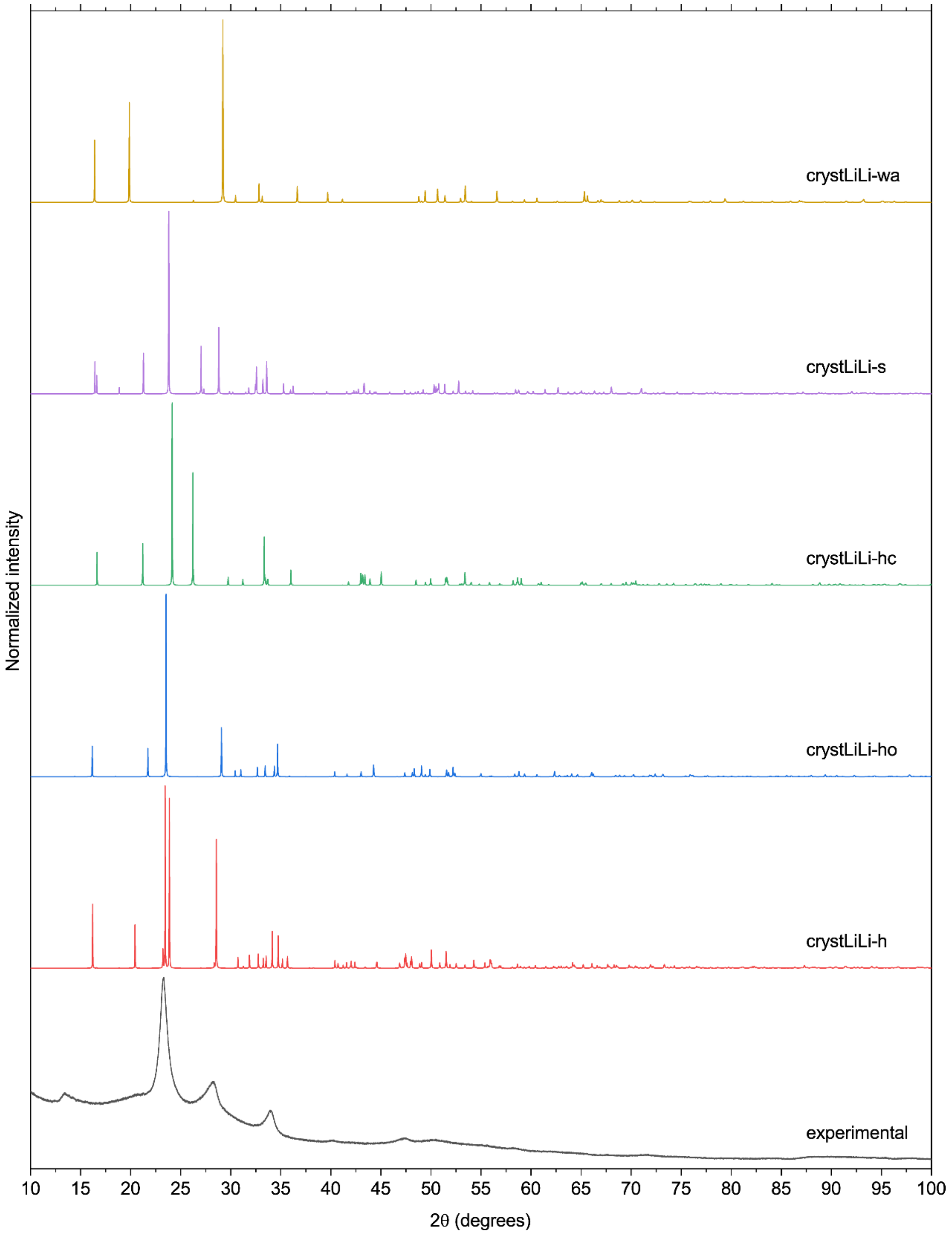

Figure S28. XRD pattern of lithiated NiBTA (0.5 V vs. Li^+/Li) compared to XRD patterns of simulated structures (CoKα radiation, λ = 1.78892 Å).

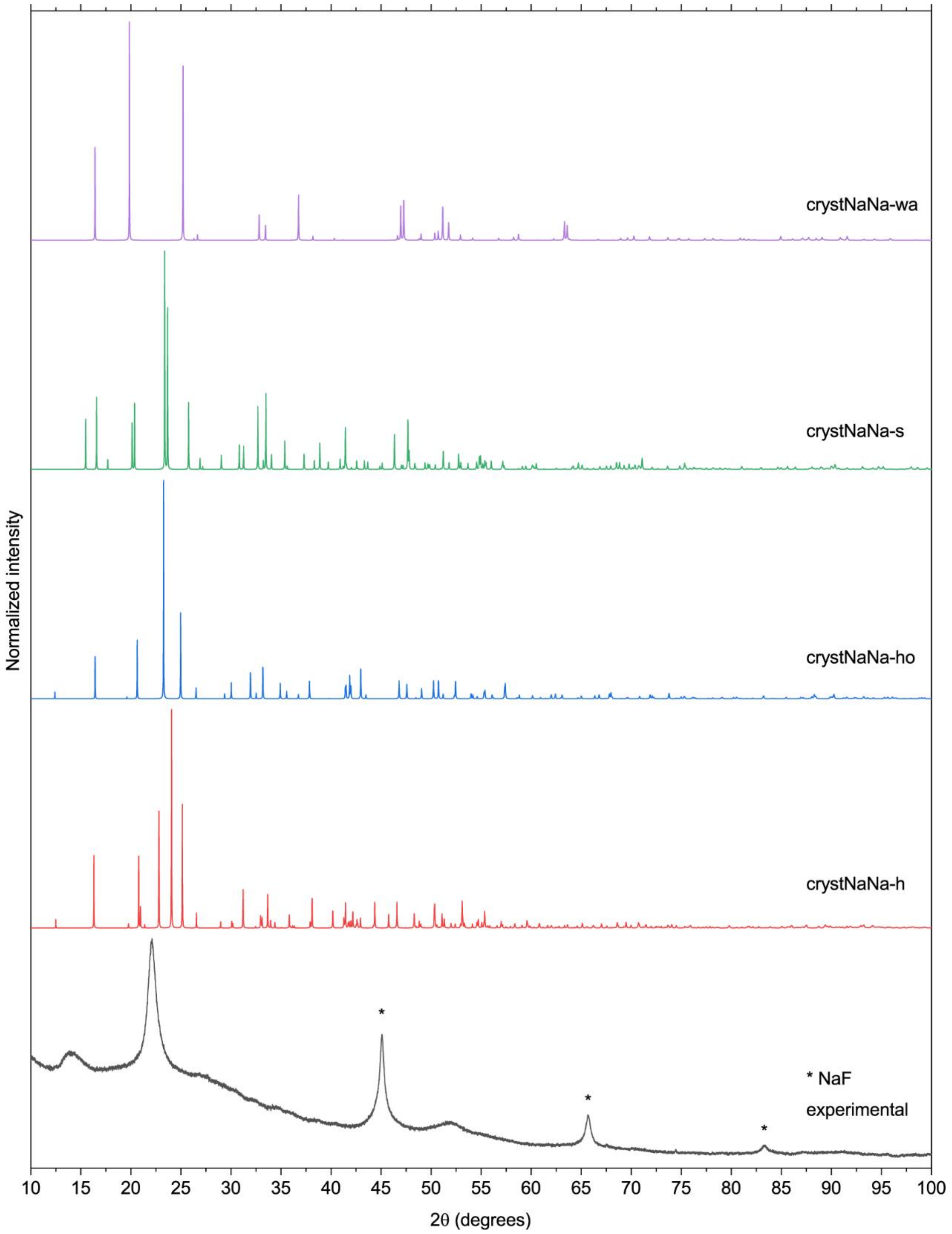

Figure S29. XRD pattern of sodiated NiBTA (0.5 V vs. Na^+/Na) compared to XRD patterns of simulated structures (CoKα radiation, λ = 1.78892 Å). Peaks of sodium fluoride are indicated in the experimental XRD pattern.

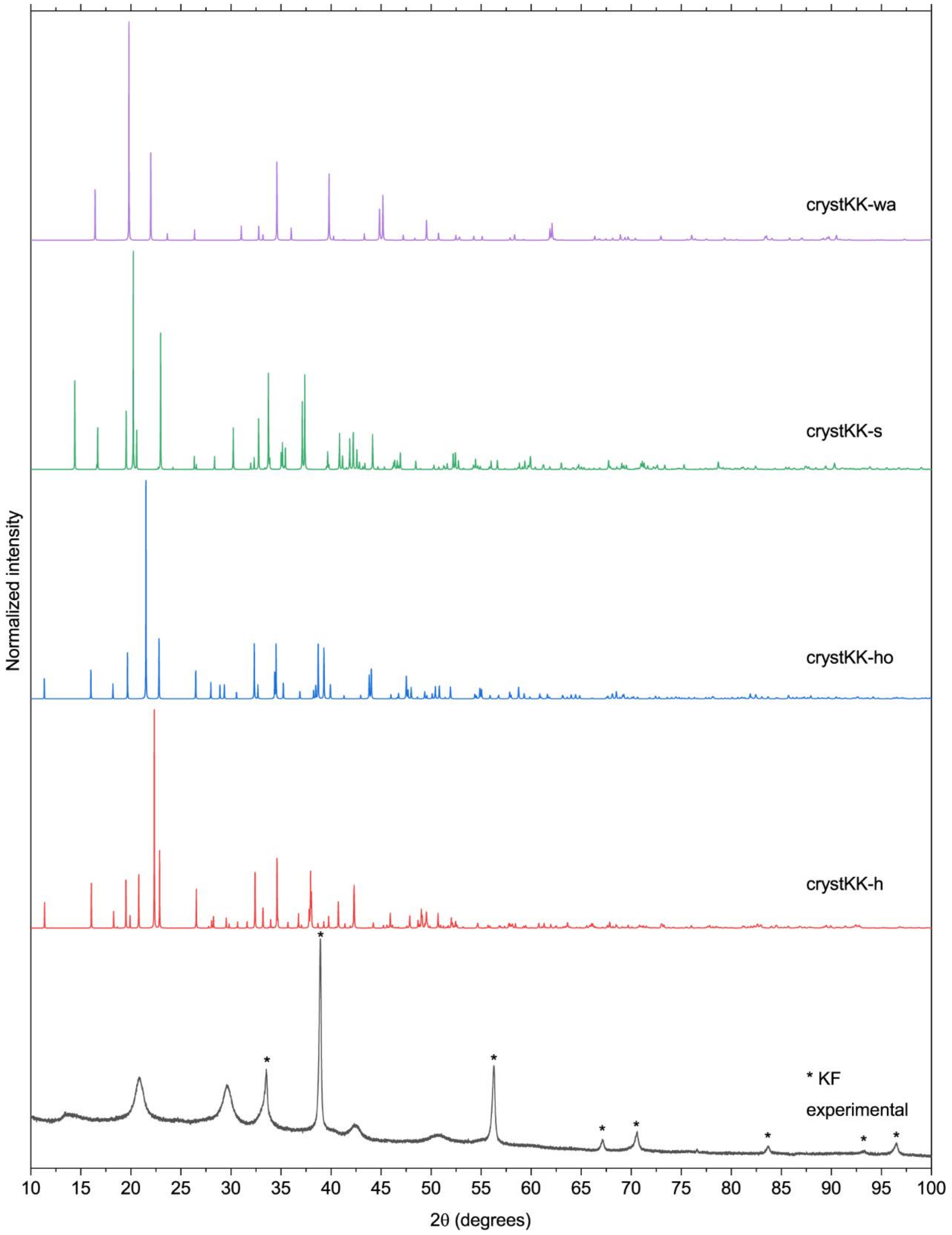

Figure S30. XRD pattern of potassiated NiBTA (0.5 V vs. Na^+/Na) compared to XRD patterns of simulated structures (CoKα radiation, λ = 1.78892 Å). Peaks of potassium fluoride are indicated in the experimental XRD pattern.

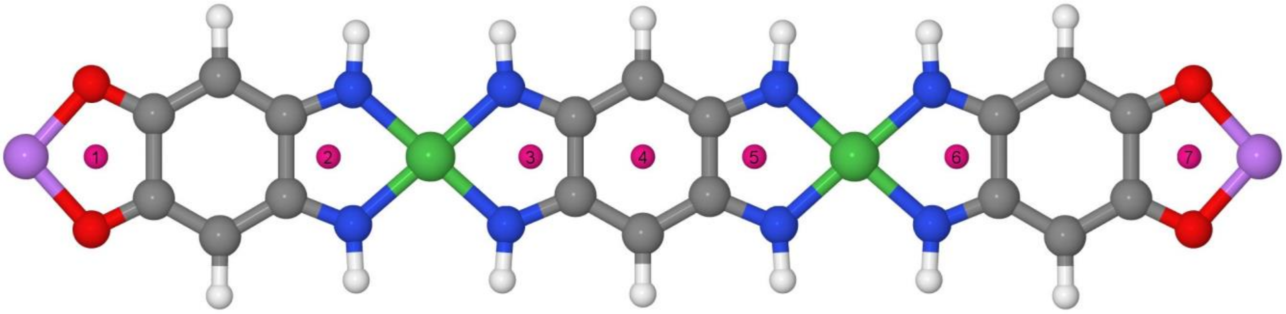

Figure S31. Stable positions of alkali atoms near oligomers. Position #4 is a saddle point. In fact, each shown position corresponds to two possible locations of an alkali atom: above and below the oligomer. Off-centerline positions (e.g., between two N atoms) are unstable.

Table S12. Possible configurations of K atoms attached to the oligomer shown in **Figure S31** at positions 1-7. The most favorable configuration is with two K atoms attached at positions 3 and 5 at the opposite faces of the oligomer. This configuration is used to set the chemical potential of K atoms when calculating energies of other configurations. In particular, from energy of configuration '3' we see that a separated pair of K atoms is 2 × 0.14 eV higher in energy then a pair localized on the same monomer.

E, eV/K-atom	configuration	filename	comment
0		2	
0	35	KKi2	
0.08	2	Kq2	
0.09	26	Ko2_T	triplet
0.11	1267	KKo2	
0.14	3	Ki2	
0.16	2266	KKo2-b	
0.18	236	K2_Q	quintet
0.23	123567	KK2	
0.25	1	Kq2-b	
0.34	4	Ki2-s	
0.34	44	KKi2-s	
0.56	35	KKi2_T	triplet

S10 Electronic structure of simulated crystalline polymers

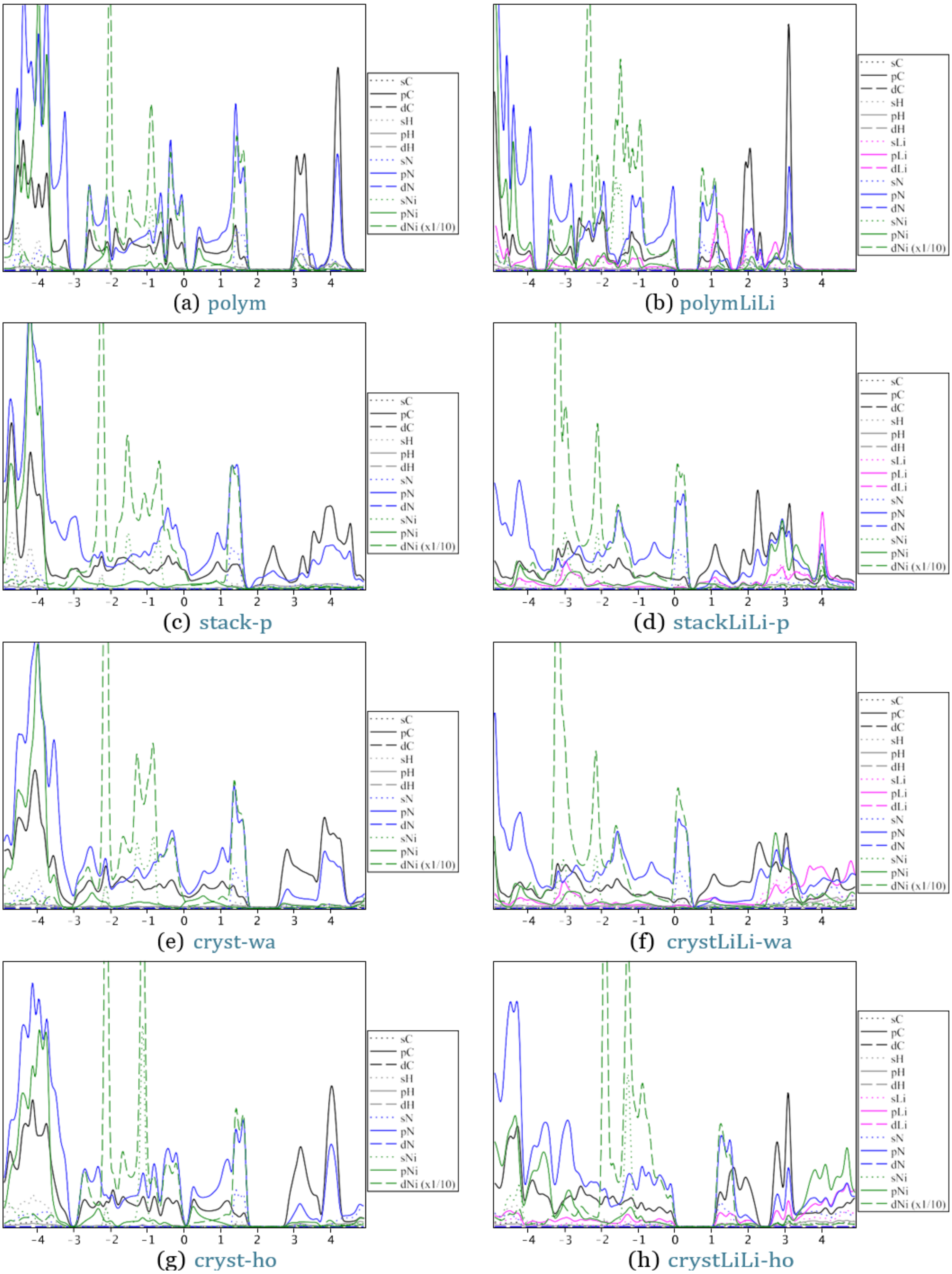

Figure S32. Electronic density of states of simulated structures.

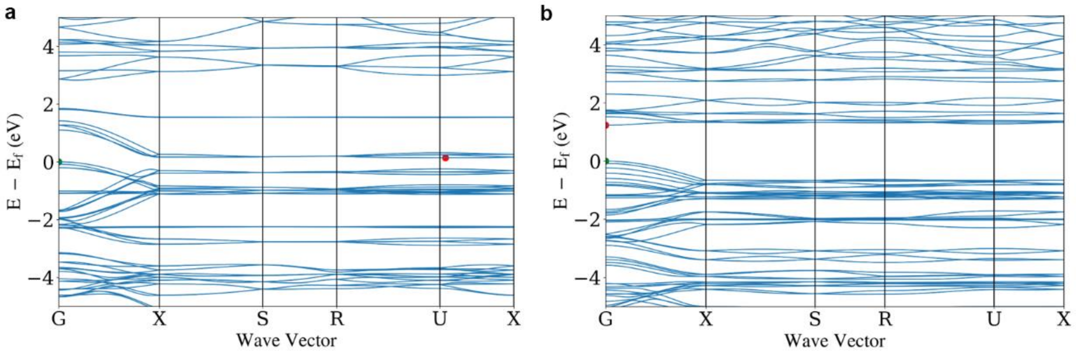

Figure S33. Band structure for crystalline NiBTA (cryst-hc) (a) and Li_2NiBTA (crystLiLi-h) (b). It is seen that pronounced dispersion of energy bands occurs only along the polymer chains (G-X), while in other directions the bands are almost flat. Green and red points show valence band maximum and conduction band minimum, respectively. Band structure of isolated macromolecules are shown in **Figure S3**.

S11 Raman spectra of NiBTA before and after cycling

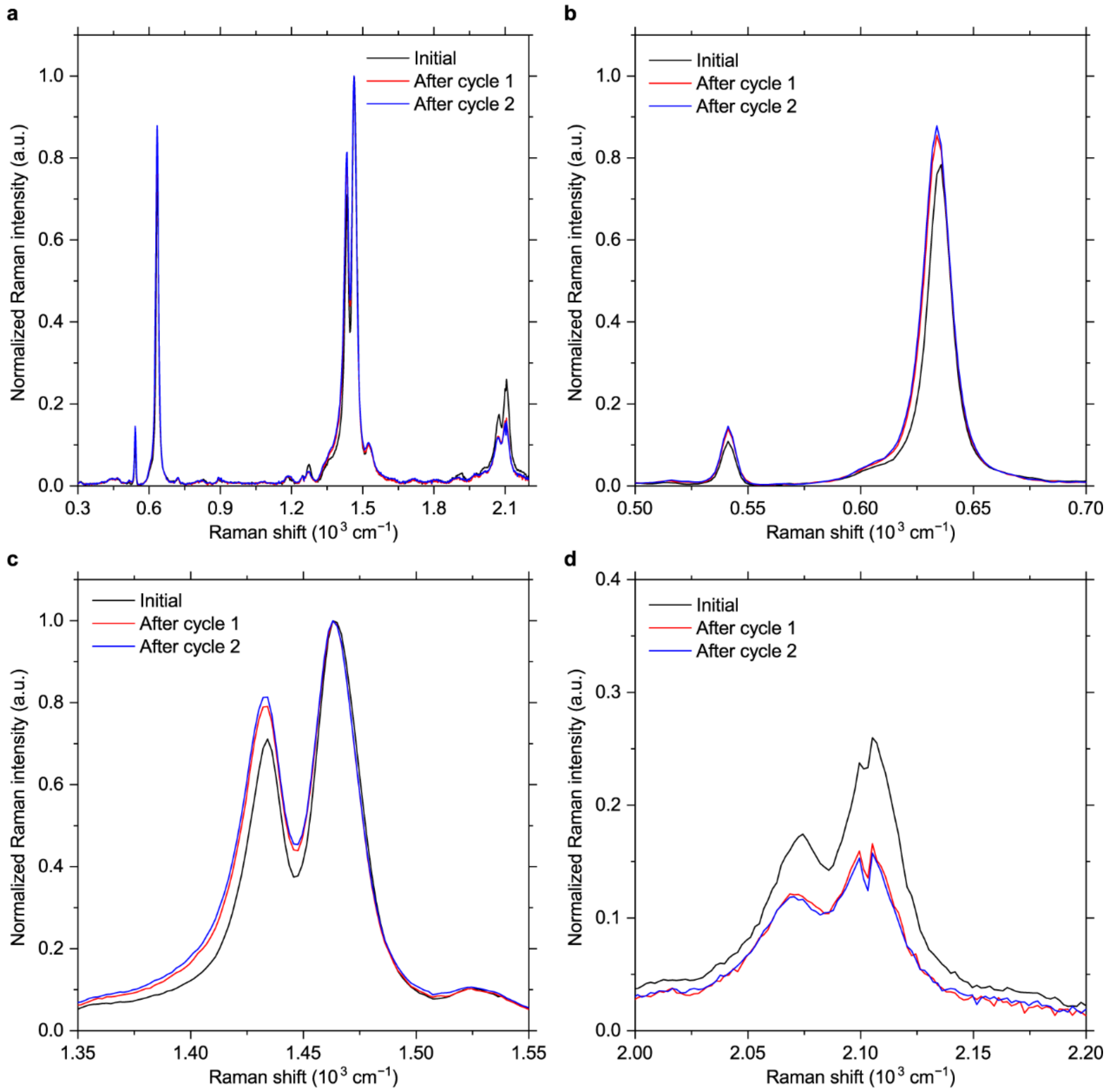

Figure S34. Normalized Raman spectra of NiBTA in the lithium-based cell before and after cycling in the 0.5–2.0 V vs. Li^+/Li potential range. The overview spectra are shown in (a), and selected regions are depicted in (b-d). The near-infrared laser (λ = 780 nm) was used for the measurements.

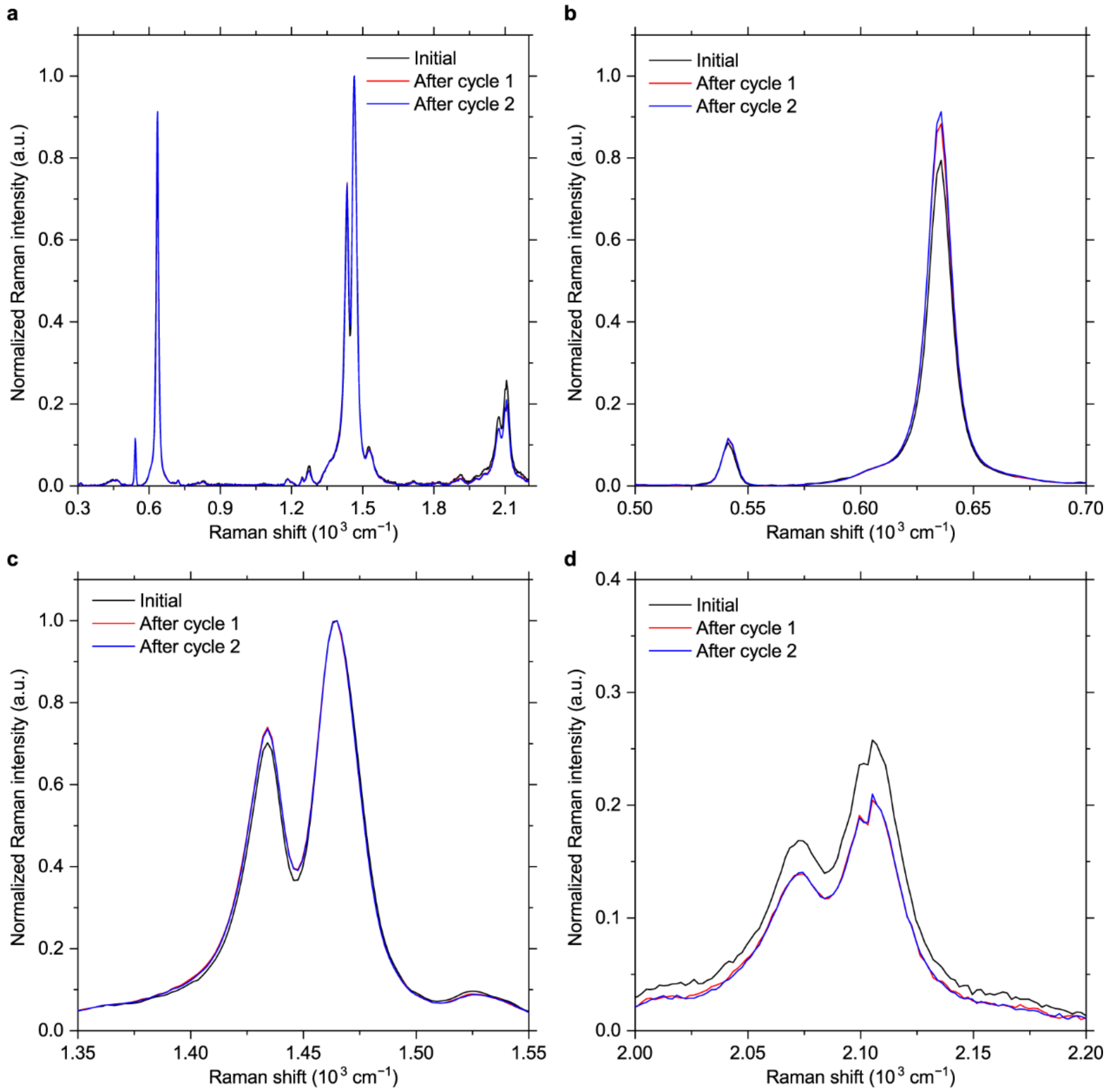

Figure S35. Normalized Raman spectra of NiBTA in the sodium-based cell before and after cycling in the 0.5–2.0 V vs. Na^+/Na potential range. The overview spectra are shown in (a), and selected regions are depicted in (b-d). The near-infrared laser (λ = 780 nm) was used for the measurements.

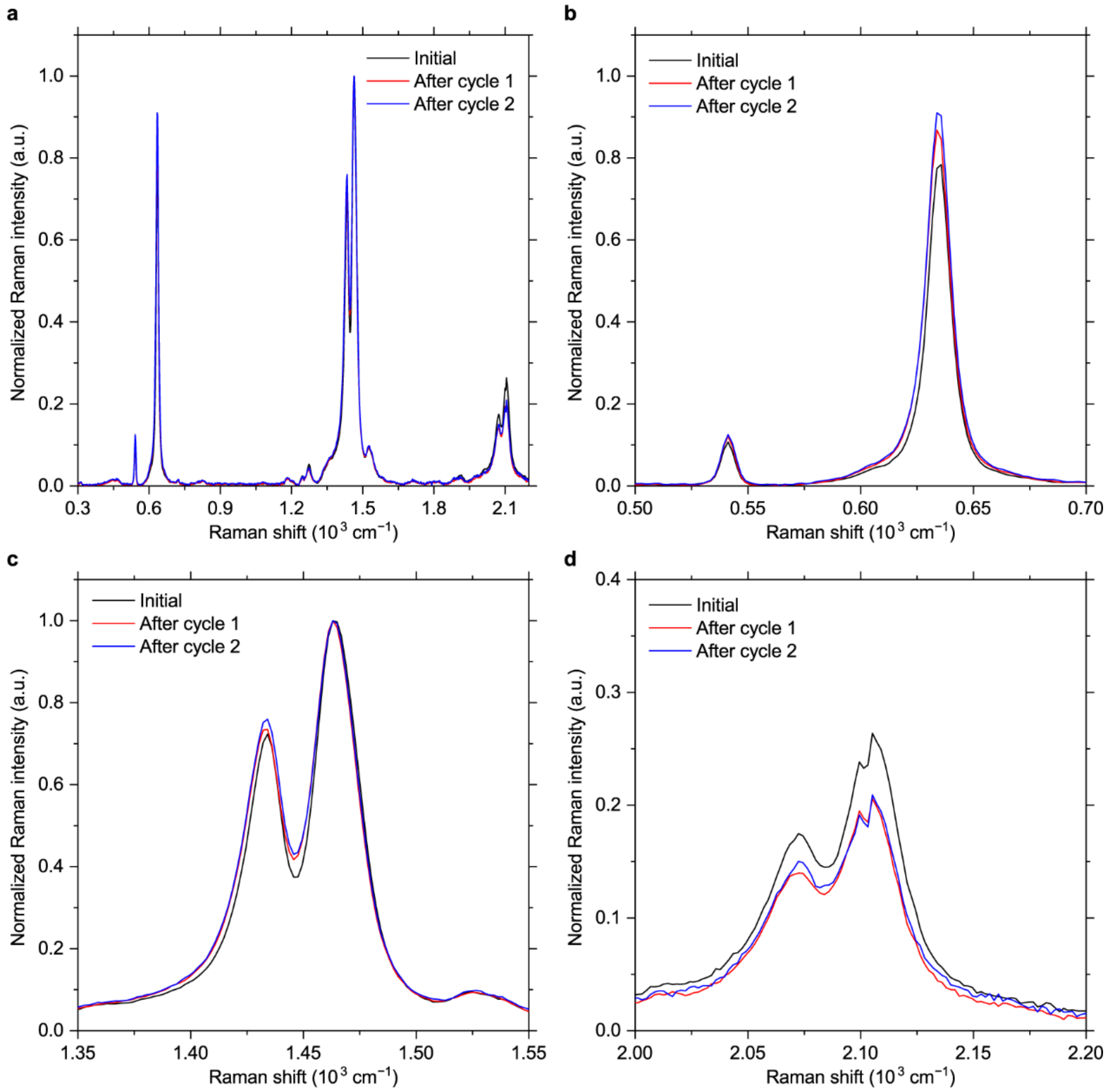

Figure S36. Normalized Raman spectra of NiBTA in the potassium-based cell before and after cycling in the 0.5–2.0 V vs. K^+/K potential range. The overview spectra are shown in (a), and selected regions are depicted in (b-d). The near-infrared laser (λ = 780 nm) was used for the measurements.

S12 Conductivity measurements

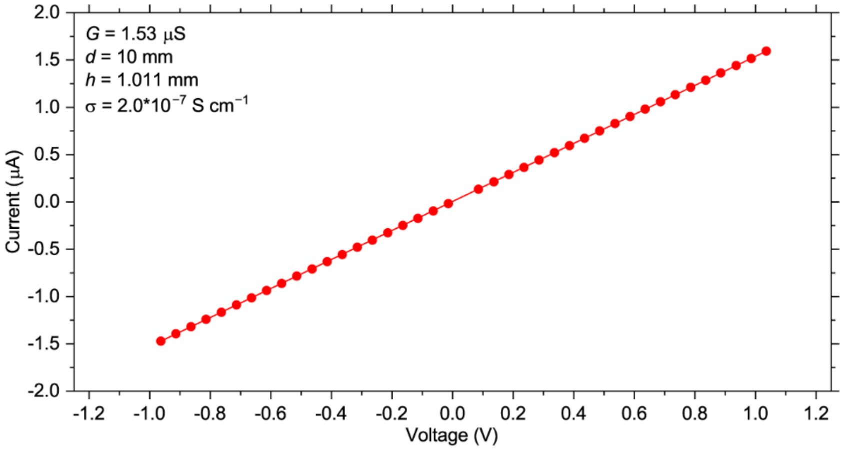

Figure S37. Direct-current polarization data for a cylindrical pellet of NiBTA. Height and diameter of the pellet are given.

S13 Raman spectra of NiBTA after chemical reduction

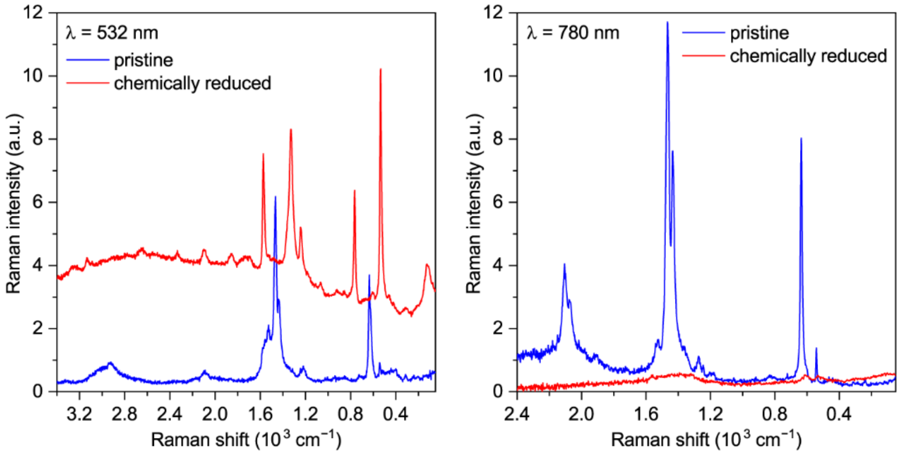

Figure S38. Raman spectra of NiBTA-based films before and after chemical reduction measured with the green (left) and near-infrared (right) lasers.

S14 Simulations of UV-Vis-NIR spectra

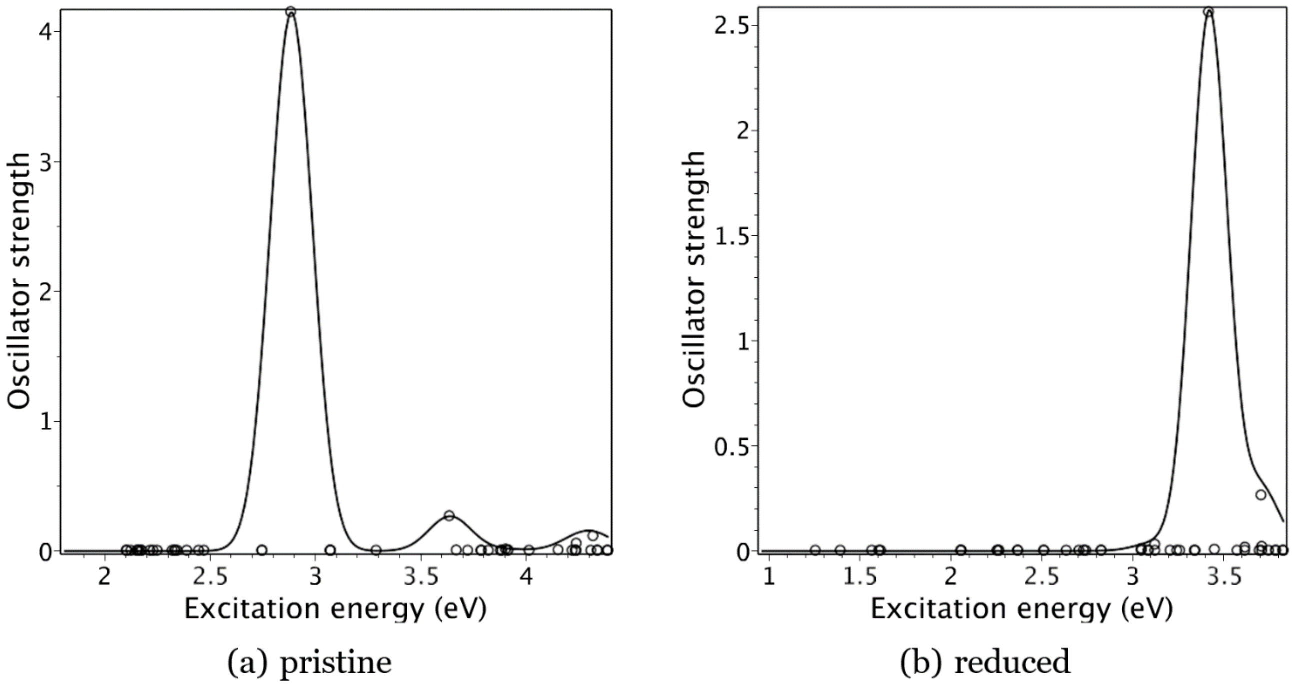

Figure S39. Typical spectra of excitations. The spectra were modeled by oligomer of 4 BTA blocks terminated as in **Figure S52**i ("reduced" state is modeled by negatively charged molecules). The lowest bright transition has B1u symmetry and corresponds to LMO5→LMO7 excitation. For the reduced oligomers the lowest bright transition is blue- shifted and corresponds to LMO7→LMO8 excitation.

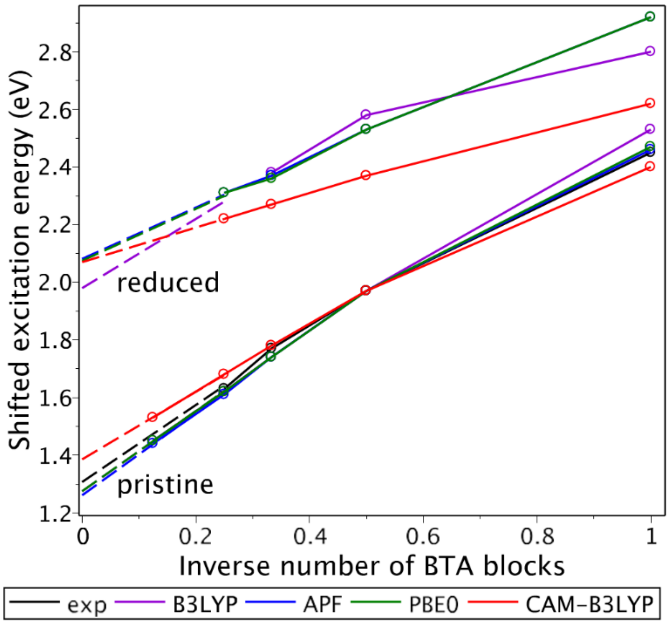

Figure S40. Calculated bright excitation energy for oligomers of various length. The oligomers are terminated as in **Figure S52**i. Experimental data are from [Audi2014]. Calculated energies are shifted by a constant to match the experimental value at a single point (pristine oligomer with 2 BTA blocks). The extrapolated energies match experimental UV-Vis peak positions for both pristine and reduced NiBTA. For the pristine polymer, the left flank of the observed absorption "band" (500–900 nm) can be attributed to oligomers since the size-dependent energy of their bright states (1.3–2.4 eV) perfectly fits this range, whereas the right flank of the absorption (>1000 nm) is formed presumably by a multitude of dipole-forbidden transitions and excitations originating from

non-passivated polymer terminations. In the reduced state, the π-conjugated system is half-filled, leading to the bandgap increase. At shorter wavelengths, the intensive absorption should originate from higher excitations and from oligomers of various length. The observed decrease of the long-wavelength absorption tail is probably associated with passivation of electronic traps (e.g., radical-like terminations of the polymer) upon NiBTA reduction, which produces intense NIR absorption of the pristine material.

S15 Calculated Raman activities

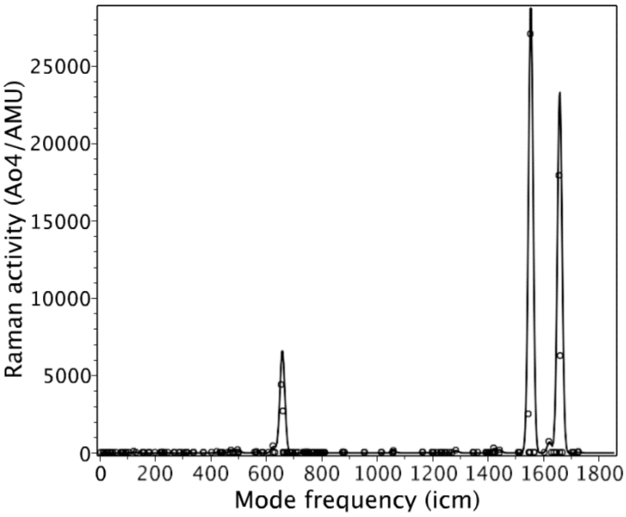

(a) pristine oligomer with 3 BTA blocks

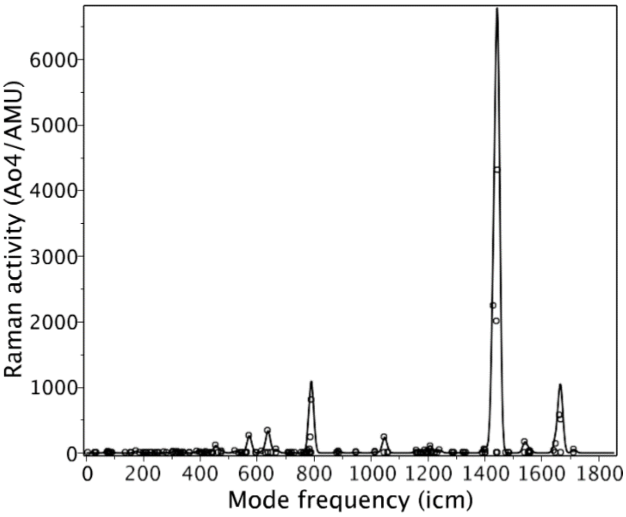

(b) reduced oligomer with 3 BTA blocks

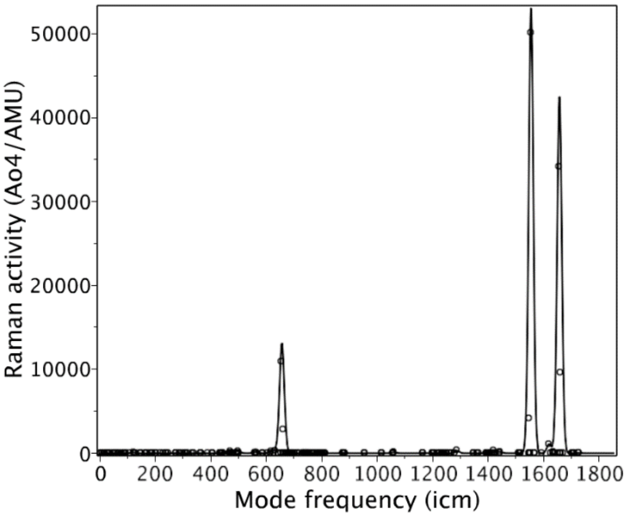

(a) pristine oligomer with 4 BTA blocks

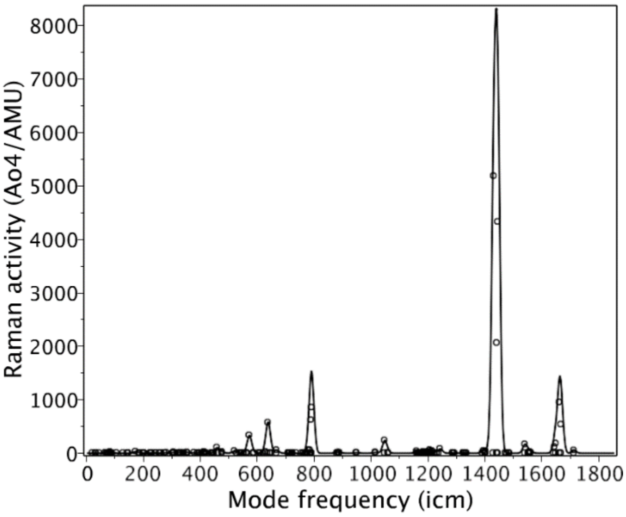

(b) reduced oligomer with 4 BTA blocks

Figure S41. Calculated Raman activities for oligomers. The oligomers are terminated as in **Figure S52**i. The reduced state is simulated by anion in a highly polarizable dielectric medium, where the charge is two times the number of BTA blocks. For the pristine oligomer, the mode at 660 cm^{-1} represents Ni-N bond stretching whereas high frequency modes at 1560 and 1660 cm^{-1} are two different BLA modes. For the reduced polymer, high frequency modes at 1450 and 1660 cm^{-1} are again BLA modes. However, at 790 cm^{-1} a new mode appears – BTA-breathing mode, whereas the Ni-N stretching mode at 640 cm^{-1} is less intense. There is also small peak at 570 cm^{-1} involving angle bending in BTA and NiN_4 fragments.

S16 Charge distributions in crystal structures

Table S13. Bader atomic charges calculated for crystalline NiBTA (cryst-hc, **Figure S23**) and Li_2NiBTA (crystLiLi-h, **Figure S25**).The nearest neighbor elements are given in parentheses. For C(-N), two non-equivalent positions are provided. It is seen that Li insertion into NiBTA causes noticeable reduction of ligands, which is further confirmed by the differential charge density revealing accumulation of electron density mainly near carbon and nitrogen atoms (**Figure S42**).

	H(-C)	H(-N)	Li	C(-N)	C*(-N)	C(-H)	N	Ni
NiBTA	+0.1	+0.4	-	+0.6	+0.5	+0	−1.2	+0.9
Li_2NiBTA	+0.0	+0.3	+0.8	+0.5	+0.1	−0.1	−1.1	+0.5

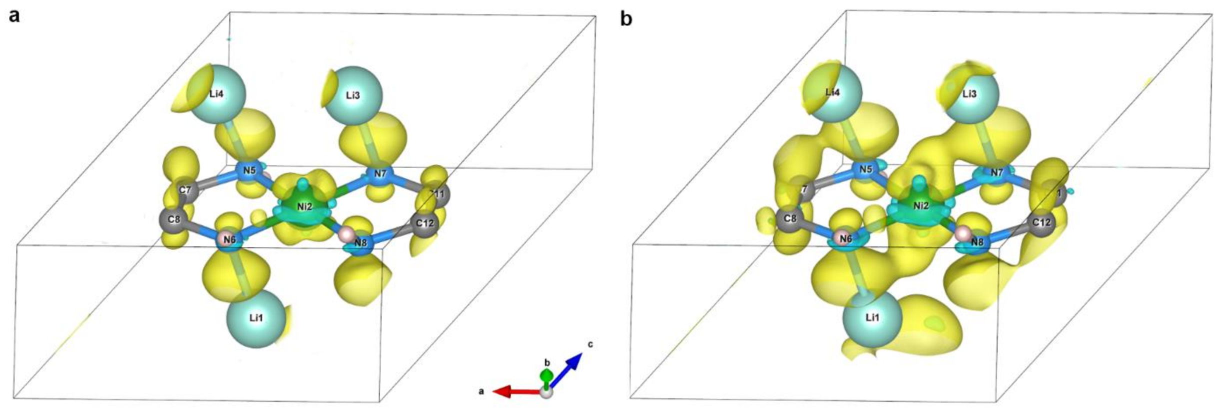

Figure S42. Differential charge density dn = n(Li_2NiBTA) - n(NiBTA) drawn for isosurface levels of 0.0085 (a) and 0.006 (b). Calculations were done for crystalline structures (cryst-hc for NiBTA and crystLiLi-h for Li_2NiBTA, see **Figure S23** and **Figure S25**). Accumulation and depletion of electron density are shown as yellow and cyan isosurfaces, respectively. During lithiation of NiBTA, electron density to is donated primarily to p_z states of nitrogen and carbon. Slight accumulation of electron density at π-hybridised d_{xz} and d_{yz} states of Ni is counteracted by density withdrawn from its d_{xy} and d_{z2} orbitals. For clarity, only central atoms are shown inside the unit cells.

S17 Electrochemistry in the 0.01–2.0 V vs. M^+/M potential ranges

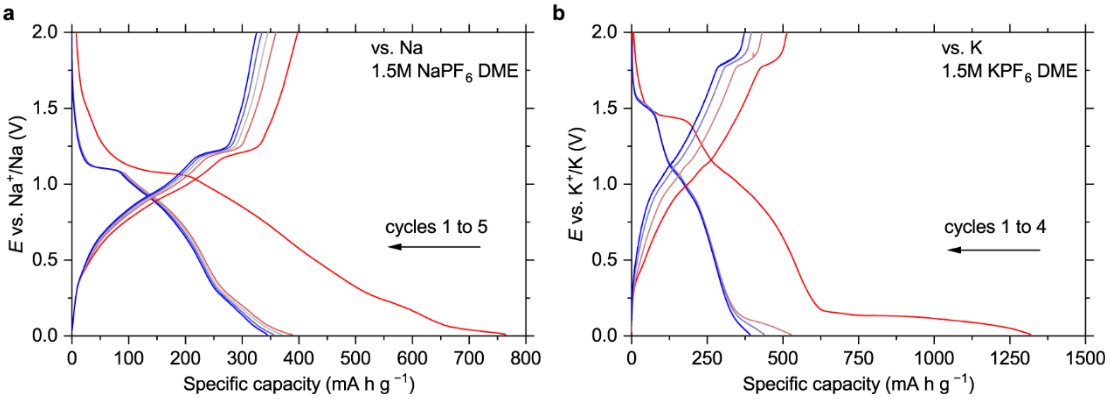

Figure S43. Electrochemistry of NiBTA in Na- and K-based cells in the 0.01–2.0 V vs. M^+/M potential ranges.Charge-discharge curves of NiBTA in sodium- (a) and potassium-based (b) cells at 0.1 A g^{-1} for initial cycles. The capacities are given per NiBTA mass. Estimated contributions from Super P are 30 and 45 mA h g^{-1} for the Na- and K-based cells, respectively (see **Figure S17**).

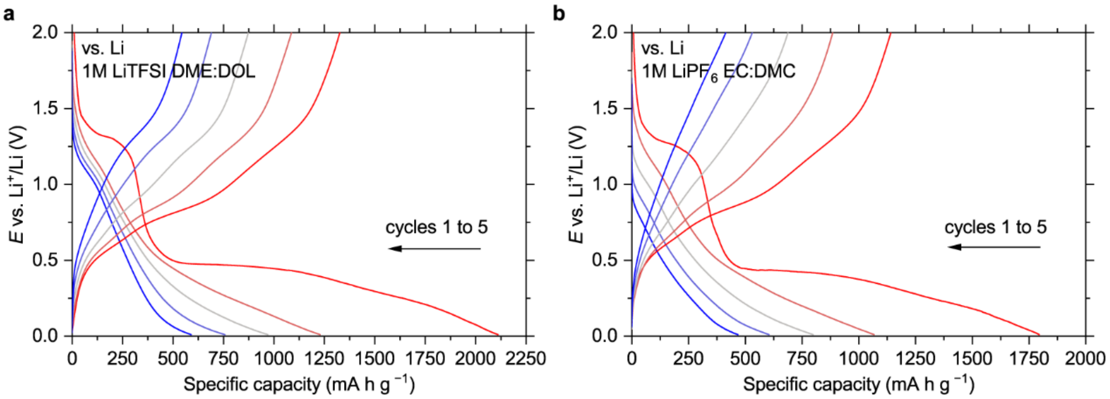

Figure S44. Electrochemistry of NiBTA in Li-based cells in the 0.01–2.0 V vs. Li^+/Li potential range.Charge-discharge curves of **NiBTA** in lithium-based cells with the ether-based (a) and carbonate-based (b) electrolytes at 0.1 A g^{-1} for initial five cycles. The capacities are given per **NiBTA** mass. Estimated contributions from Super P are 50 and 55 mA h g^{-1} for the carbonate- and ether-based electrolytes, respectively (see **Figure S16**).

S18 Raman spectroscopy in the 0.01–2.0 V vs. M^+/M potential ranges

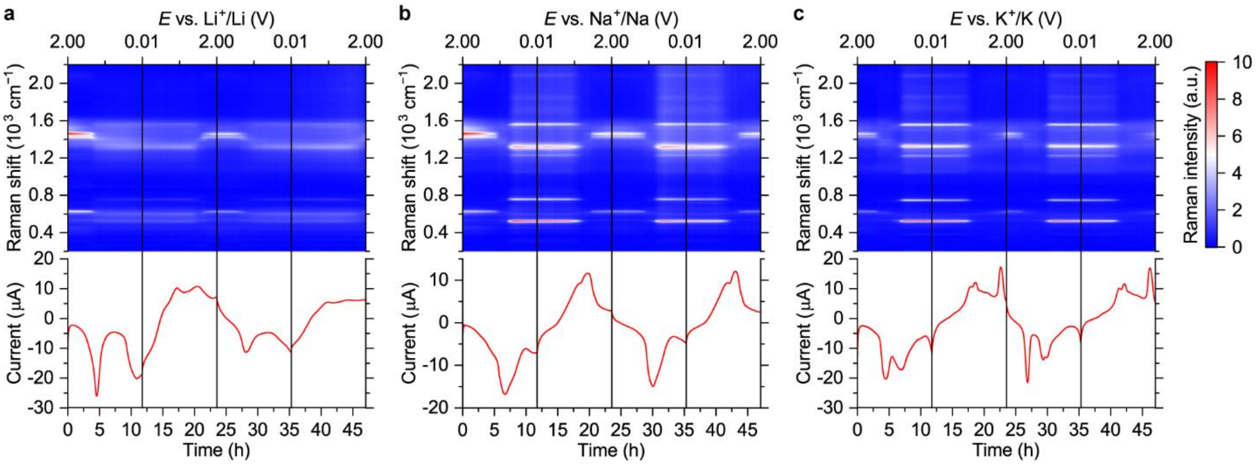

Figure S45. Evolution of Raman spectra of NiBTA in the 0.01–2.0 V vs. M^+/M potential ranges for Li- (a), Na- (b) and K-based (c) cells. Raman intensity maps and current vs. time (vs. potential) profiles are plotted. The green laser (λ = 532 nm) was used for the measurements.

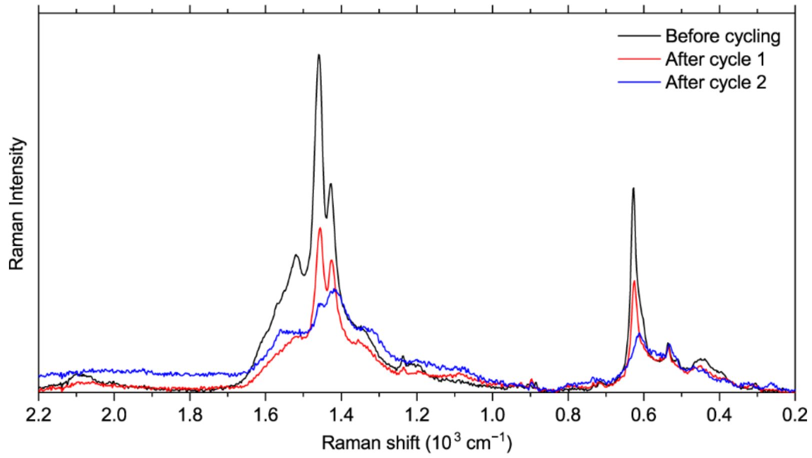

Figure S46. *Operando* Raman spectra of NiBTA in the Li-based cell at 2.0 V vs. Li^+/Li before and after cycling. The green laser (λ = 532 nm) was used for the measurements.

S19 TEM EDX data before and after deep reduction

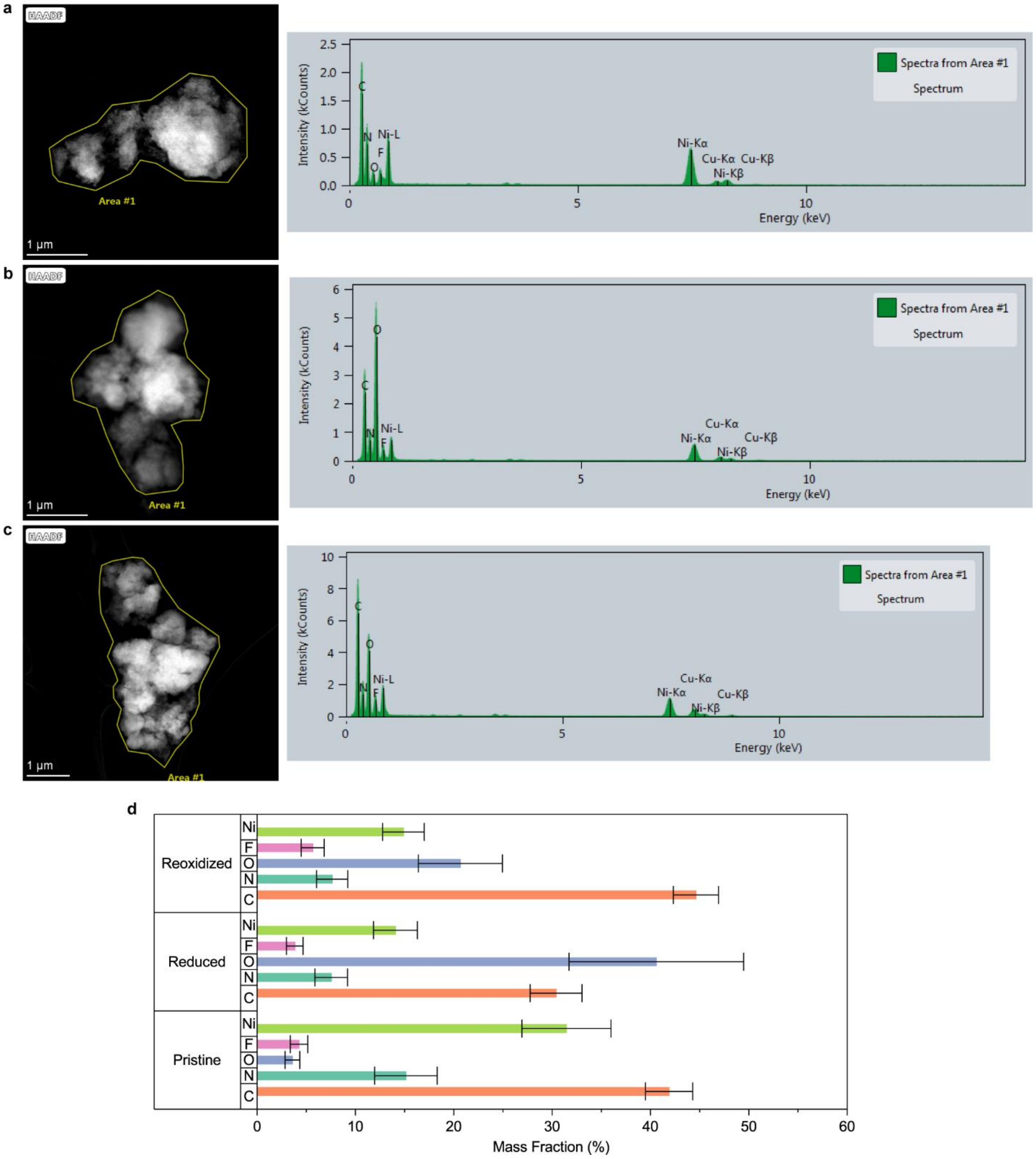

Figure S47. EDX data of NiBTA before/after deep cycling in Li-based cells.EDX spectra for selected sample areas of pristine electrode (a), electrode after lithiation to 0.01 V vs. Li^+/Li (reduced) (a), and electrode after lithiation to 0.01 V and delithiation to 2.0 V vs. Li^+/Li (reoxidized) (c); estimated mass fractions of elements (d).

S20 Benchmarking for diradical-type oligomer Ni-OPD_2

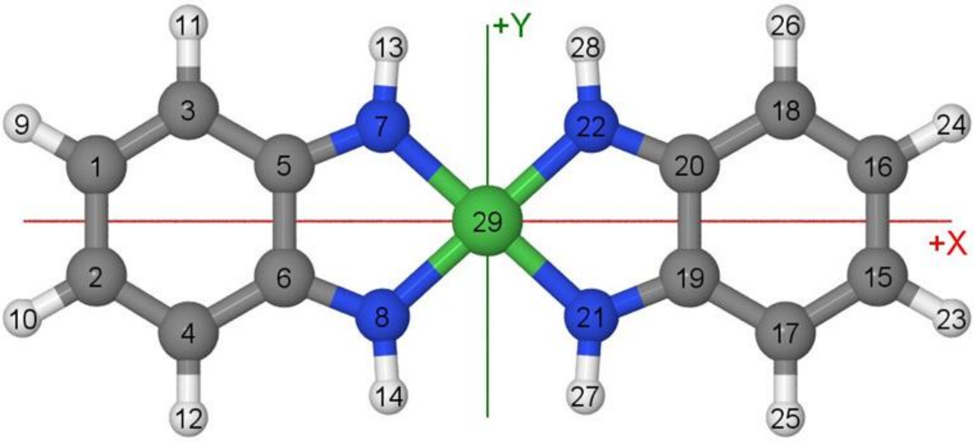

Figure S48. Orientation and atom indexing of Ni-OPD_2.

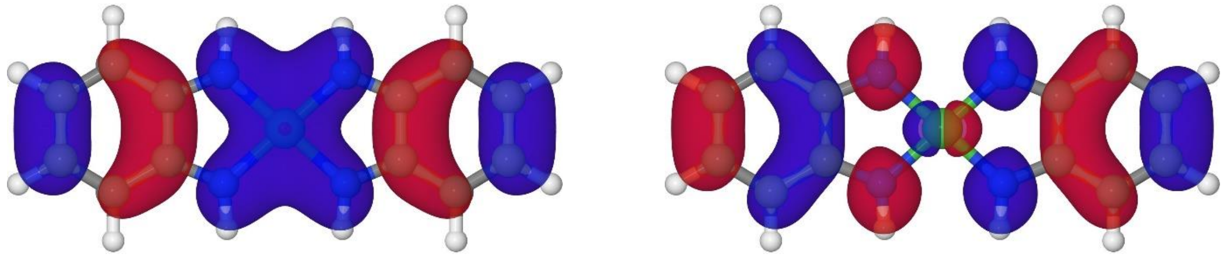

HOMO, B1u Z　　　　LUMO, B2g XZ

Figure S49. Frontier molecular orbitals of Ni-OPD_2.

Table S14. Methods benchmarking for IP and EA of the neutral singlet state. Here IP is ionization potential, EA is electron affinity, E^H is HOMO energy, E^L is LUMO energy, E_g^c = IP − EA is charge gap, $E_g^{HL} = E^L - E^H$ is HOMO-LUMO gap, $\Delta E_g = (E_g^c - E_g^{HL})/2 \equiv (\Delta IP - \Delta EA)/2$, $\Delta\chi = (\Delta IP + \Delta EA)/2$, ΔIP = IP + E^H, ΔEA = EA + E^L. All entries are energies in eV. Rows are ordered by ΔE_g.

method	ΔE_g	$\Delta\chi$	E_g^c	E_g^{HL}	ΔIP	IP	$-E^H$	ΔEA	EA	$-E^L$
Different functionals with 'a3p' basis										
PBE	1.92	-0.01	4.76	0.92	1.92	6.30	4.39	-1.93	1.54	3.47
B3LYP	1.41	0.02	4.34	1.52	1.43	6.13	4.70	-1.39	1.79	3.18
APF	1.33	0.02	4.33	1.68	1.34	6.15	4.81	-1.31	1.82	3.13
PBE0	1.27	0.02	4.29	1.75	1.29	6.12	4.83	-1.25	1.83	3.08
CAM-B3LYP	0.37	0.01	3.91	3.16	0.39	5.95	5.57	-0.36	2.05	2.41
WB97X	-0.43	0.00	3.61	4.47	-0.43	5.80	6.23	0.43	2.19	1.76
Different basis sets with CAM-B3LYP functional										
p2p/a3p	0.41	0.01	4.02	3.20	0.43	5.68	5.25	-0.40	1.66	2.06
a3p	0.37	0.01	3.91	3.16	0.39	5.95	5.57	-0.36	2.05	2.41
p2p	0.36	0.02	4.14	3.42	0.38	5.79	5.41	-0.33	1.66	1.99

Table S15. Methods benchmarking for singlet excitations of the neutral singlet state. The three lowest excited states are considered: B3u, B1g, B2g. Here E is the excitation energy and f is the oscillator strength (zero for B1g and B2g), the superscript 'rel' means geometrically relaxed state. All energies are given in eV. Rows are ordered by E_g^{HL}.

method	E_g^{HL}	E_{B3u}	f_{B3u}	E_{B1g}	E_{B2g}	E^{rel}_{B3u}	E^{rel}_{B1g}
Different functionals with 'a3p' basis							
PBE	0.92	1.94	(0.53)	1.38	1.80	—	1.27
B3LYP	1.52	1.82	(0.58)	1.71	2.07	1.71	1.51
APF	1.68	1.87	(0.61)	1.74	2.13	1.75	1.54
PBE0	1.75	1.86	(0.62)	1.78	2.17	1.75	1.56
CAM-B3LYP	3.16	1.71	(0.64)	1.86	2.23	1.59	1.60
WB97X	4.47	1.58	(0.65)	1.84	2.19	1.44	1.56
Different basis sets with CAM-B3LYP functional							
a3p	3.16	1.71	(0.64)	1.86	2.23	1.59	1.60
p2p/a3p	3.20	1.75	(0.62)	1.85	2.25	1.62	1.60
p2p	3.42	1.89	(0.67)	1.66	2.07	1.78	1.45

Table S16. Benchmarking methods for spin gaps with relaxed geometries at mmm-symmetry. ST = singlet- triplet, TQ = triplet-quintet, DQ = doublet-quartet. The superscript denotes the charge of the molecule. For the anion, the Ag state is chosen. All energies are given in eV. Rows are ordered by E_g^{HL}.

method	E_g^{HL}	ΔE_{ST}	ΔE_{TQ}	ΔE^{-}_{DQ}	ΔE^{2-}_{DQ}
Different functionals with 'a3p' basis					
PBE	0.92	0.68	1.10	—	0.50
B3LYP	1.52	0.22	0.66	0.84	0.29
APF	1.68	0.20	0.62	0.82	0.27
PBE0	1.75	0.15	0.55	0.76	0.23

CAM-B3LYP	3.16	-0.24	0.64	0.88	0.35
WB97X	4.47	-0.52	0.63	0.92	—
Different basis sets with CAM-B3LYP functional					
a3p	3.16	-0.24	0.64	0.88	0.35
p2p/a3p	3.20	-0.25	0.61	0.86	0.32
p2p	3.42	-0.01	0.53	0.85	1.19

Table S17. Methods benchmarking for Raman active modes. The column ‘IR’ shows maximum IR intensity in KM/Mole. The column ‘Raman’ shows maximum Raman activity in Å^4/AMU. Then the five Raman most active modes are listed: frequency in cm^{-1} and activity relative to the maximum value given in the 4th column. Rows are ordered by E_g^{HL}. Three states are considered here: singlet, triplet and dianion. Modes numbers for CAM-B3LYP/a3p are given in the same row as the state is defined.

method	E_g^{HL}	IR	Raman	ω_1	f_1	ω_2	f_2	ω_3	f_3	ω_4	f_4	ω_5	f_5
Ground state singlet ‘S’				59		63		26		67		8	
Different functionals with ‘a3p’ basis													
PBE	0.92	383	2510	1366	(1)	1489	(.18)	655	(.08)	1542	(.17)	226	(.06)
B3LYP	1.52	1090	4754	1394	(1)	1522	(.17)	654	(.09)	1580	(.11)	229	(.05)
APF	1.68	1180	4708	1419	(1)	1542	(.26)	664	(.11)	1601	(.12)	233	(.05)
PBE0	1.75	1331	4808	1424	(1)	1548	(.28)	664	(.11)	1606	(.11)	233	(.05)
CAM-B3LYP	3.16	3675	12621	1421	(1)	1545	(.30)	670	(.14)	1618	(.09)	237	(.04)
WB97X	4.47	5323	28322	1427	(1)	1546	(.43)	673	(.18)	1630	(.10)	239	(.03)
Different basis sets with CAM-B3LYP functional													
a3p	3.16	3675	12621	1421	(1)	1545	(.30)	670	(.14)	1618	(.09)	237	(.04)
p2p/a3p	3.20	3442	10376	1438	(1)	1563	(.33)	672	(.14)	1636	(.08)	238	(.04)
p2p	3.42	2883	9811	1437	(1)	1558	(.34)	701	(.14)	1642	(.09)	249	(.03)
Ground state triplet ‘T’				65		67		28		62		59	
Different functionals with ‘a3p’ basis													
PBE	1.08	205	3543	1502	(1)	1530	(.06)	635	(.07)	1469	(.15)	1375	(.00)
B3LYP	3.29	245	2807	1538	(1)	1569	(.09)	636	(.22)	1514	(.08)	1397	(.02)
APF	3.76	253	3139	1564	(1)	1590	(.10)	642	(.22)	1534	(.08)	1423	(.02)
PBE0	4.02	256	2817	1571	(1)	1595	(.13)	643	(.23)	1540	(.09)	1429	(.02)
CAM-B3LYP	6.39	376	1543	1580	(1)	1607	(.30)	648	(.24)	1548	(.16)	1418	(.12)
WB97X	8.26	467	1055	1595	(1)	1619	(.69)	643	(.27)	1555	(.23)	1420	(.26)
Different basis sets with CAM-B3LYP functional													
a3p	6.39	376	1543	1580	(1)	1607	(.30)	648	(.24)	1548	(.16)	1418	(.12)
p2p/a3p	6.39	319	1602	1601	(1)	1623	(.21)	647	(.22)	1567	(.16)	1436	(.13)
p2p	6.74	236	4623	1596	(1)	1624	(.04)	668	(.20)	1568	(.08)	1435	(.03)
Dianion ‘NN’				61		66		40		57		30	
Different functionals with ‘a3p’ basis													
PBE	1.14	887	1019	1405	(1)	1527	(.06)	769	(.33)	1311	(.50)	620	(.35)
B3LYP	3.41	1246	420	1439	(1)	1554	(.31)	784	(.45)	1326	(.35)	631	(.30)
APF	3.85	1338	440	1451	(1)	1578	(.27)	794	(.44)	1351	(.26)	637	(.32)
PBE0	4.03	1367	428	1454	(1)	1583	(.29)	796	(.44)	1355	(.23)	638	(.31)

CAM-B3LYP	5.90	1548	359	1460	(1)	1579	(.54)	798	(.42)	1341	(.22)	645	(.22)
WB97X	7.91	1712	356	1461	(1)	1585	(.64)	799	(.39)	1343	(.18)	638	(.17)
Different basis sets with CAM-B3LYP functional													
a3p	5.90	1548	359	1460	(1)	1579	(.54)	798	(.42)	1341	(.22)	645	(.22)
p2p/a3p	6.54	1407	281	1482	(1)	1612	(.74)	803	(.44)	1368	(.26)	644	(.22)
p2p	6.01	1285	400	1485	(1)	1615	(.58)	808	(.31)	1368	(.32)	666	(.25)

Table S18. Crystal geometry: experiment at 295 K vs. calculated (at 0 K). The space group is P21/c.

method	volume $Å^3$/atom	*a* Å	*b* Å	*c* Å	*β* deg
experiment [Hall1968]	9.53	5.84	7.48	25.27	90.2
PBE-D3/PAW600	9.23	5.79	7.36	25.12	90.4
PBE-D3/PAW900	9.19	5.82	7.29	25.15	90.1

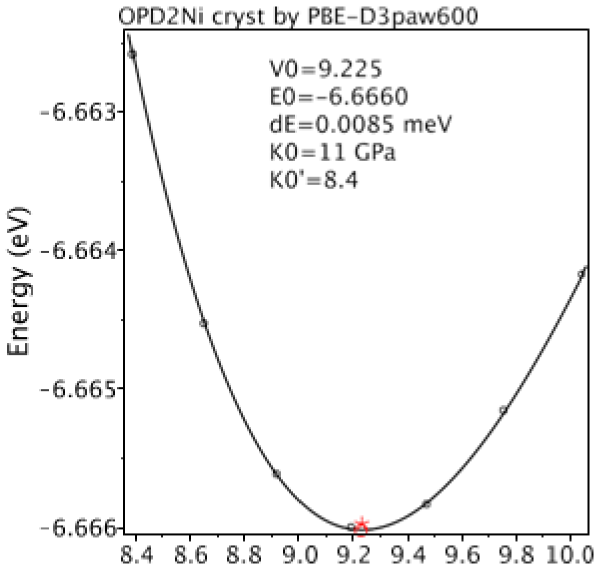

Figure S50. Relaxed volume scan for determination of parameters of equation of state of the crystal.

Table S19. Summary table for vibrations of the crystal (Γ-point only).

```
PBE-D3/paw600
N=348, ZPE=69.95 meV/mode, F(300K)=74.54 meV/mode
Frequencies: -1.61,-1.28,-0.30,2.13,2.82,3.17,4.43,5.12,5.96 .. 426,426,426 meV
Elastic tensor in GPa:
 XX -119.147
 YY -127.895 -126.899
ZZ  -82.851  -90.007  -26.266
XY   -0.000   -0.000   -0.000    8.944
YZ   -0.000   -0.000    0.000    1.467    1.695
ZX    1.273    1.332   -6.867    0.000   -0.000    6.636
       XX       YY       ZZ       XY       YZ       ZX
ts Ieigenelements:
   -304.700    1.409    3.714    5.393    9.229   29.916
XX    0.634    0.000   -0.568   -0.491   -0.000    0.185
YY    0.660   -0.000    0.384    0.541    0.000    0.352
ZZ    0.402   -0.000    0.259   -0.108    0.000   -0.871
XY    0.000    0.191   -0.000    0.000   -0.982   -0.000
YZ    0.000   -0.982   -0.000   -0.000   -0.191   -0.000
ZX    0.003   -0.000    0.681   -0.674   -0.000    0.287
Pulay stress: false pressure=-0.323, external pressure by components:
     -2.880   -1.157   -3.597   -0.315   -0.307    0.030
      2.470    0.566    3.312   -0.317   -0.307   -0.634
```

S21 Geometric definitions used for the calculations

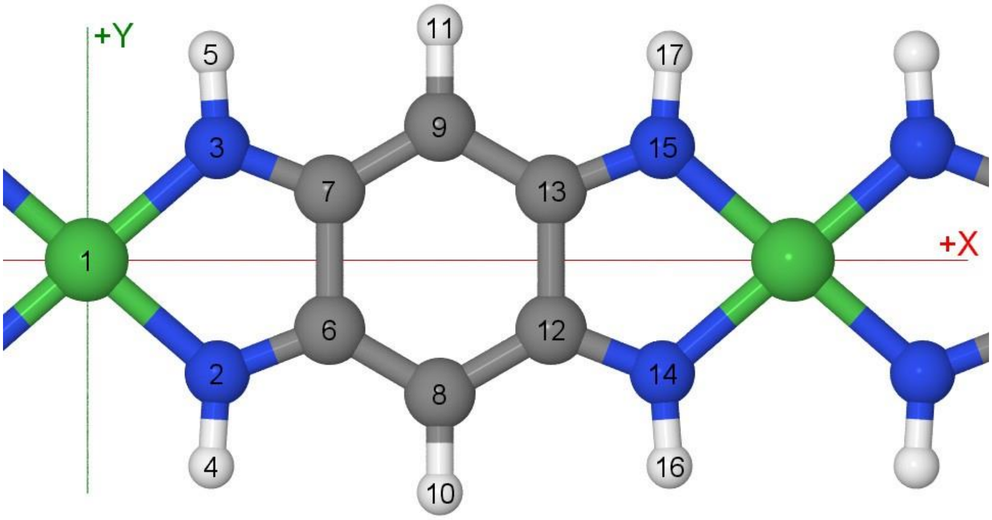

Figure S51. Orientation and atom indexing of NiBTA used for the calculations.

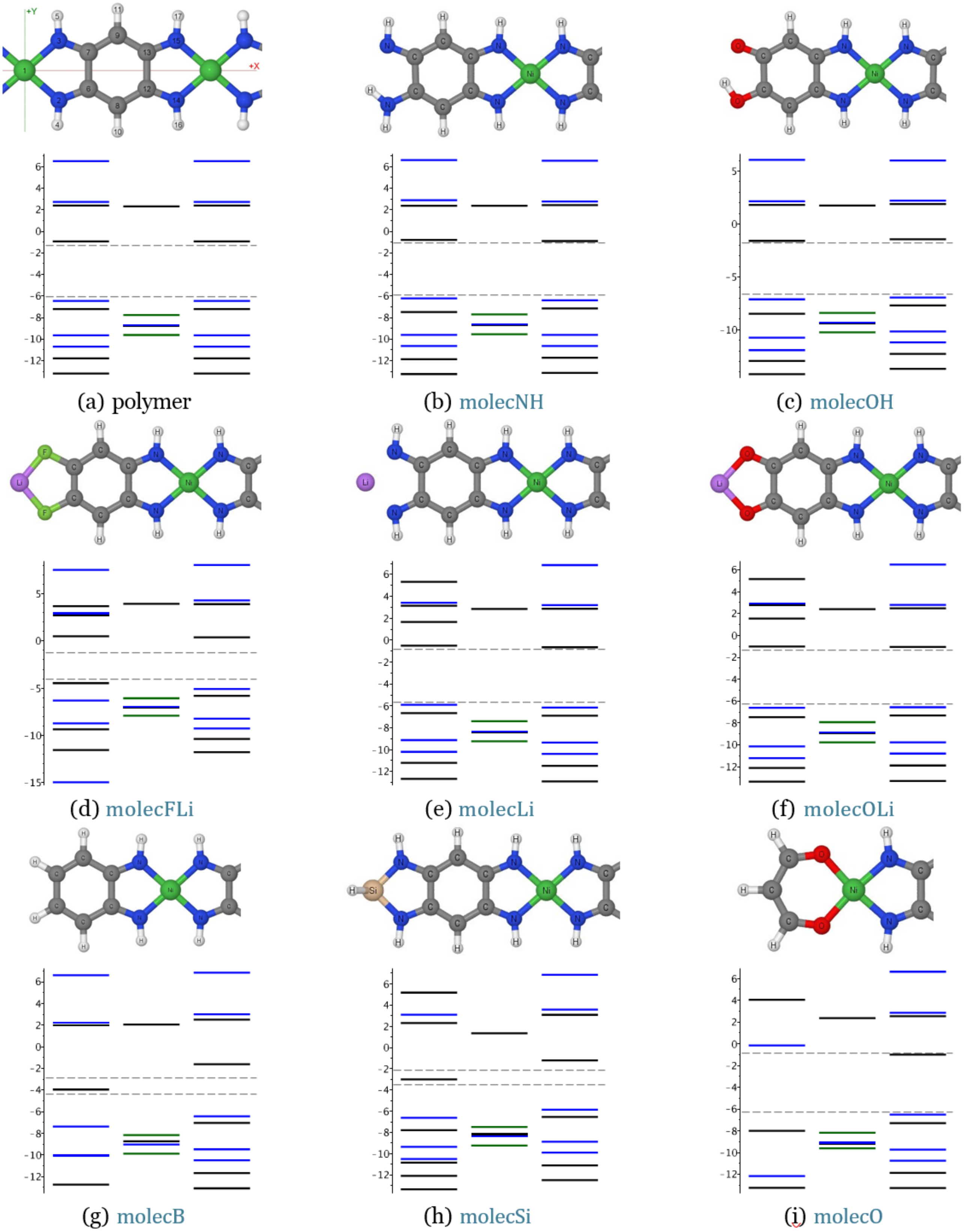

Figure S52. Considered terminations of NiBTA oligomers. The electronic levels are given for LMOs localized on the terminal group (left), Ni (center) and BTA monomer (right). Evidently, all considered terminations do not distort LMOs of the BTA substantially. Natural terminations (b, c) induce the smallest distortion but have low symmetry which is inconvenient for calculations. Replacement of nonsymmetric hydrogen by an alkaline atom (d, e, f) is consistent with the chemistry of the studied system, but the valence *p*-orbitals of the metal stands next to the LUMO

(working LMO) of the BTA. Symmetric passivations (g, h) are free from intruder LMOs but they are diradicals (the LMO in the bandgap is half-occupied).

References

AUDI, H., CHEN, Z., CHARAF-EDDIN, A., D'ALÉO, A., CANARD, G., JACQUEMIN, D. & SIRI, O. 2014. Extendable nickel complex tapes that reach NIR absorptions. *Chemical Communications,* 50**,** 15140-15143.

HALABY, S., MARTYNOWYCZ, M. W., ZHU, Z., TRETIAK, S., ZHUGAYEVYCH, A., GONEN, T. & SEIFRID, M. 2021. Microcrystal Electron Diffraction for Molecular Design of Functional Non-Fullerene Acceptor Structures. *Chemistry of Materials,* 33**,** 966-977.

HALL, G. S. & SODERBERG, R. H. 1968. Crystal and molecular structure of bis(o-phenylenediamino)nickel, Ni[C6H4(NH)2]2. *Inorganic Chemistry,* 7**,** 2300-2303.

KAPAEV, R. R., OLTHOF, S., ZHIDKOV, I. S., KURMAEV, E. Z., STEVENSON, K. J., MEERHOLZ, K. & TROSHIN, P. A. 2019. Nickel(II) and Copper(II) Coordination Polymers Derived from 1,2,4,5-Tetraaminobenzene for Lithium-Ion Batteries. *Chemistry of Materials,* 31**,** 5197-5205.

LERICHE, J. B., HAMELET, S., SHU, J., MORCRETTE, M., MASQUELIER, C., OUVRARD, G., ZERROUKI, M., SOUDAN, P., BELIN, S., ELKAÏM, E. & BAUDELET, F. 2010. An Electrochemical Cell for Operando Study of Lithium Batteries Using Synchrotron Radiation. *Journal of The Electrochemical Society,* 157**,** A606.

ONG, S. P., RICHARDS, W. D., JAIN, A., HAUTIER, G., KOCHER, M., CHOLIA, S., GUNTER, D., CHEVRIER, V. L., PERSSON, K. A. & CEDER, G. 2013. Python Materials Genomics (pymatgen): A robust, open-source python library for materials analysis. *Computational Materials Science,* 68**,** 314-319.

TANG, W., SANVILLE, E. & HENKELMAN, G. 2009. A grid-based Bader analysis algorithm without lattice bias. *Journal of Physics: Condensed Matter,* 21**,** 084204.

TUKACHEV, N. V., MASLENNIKOV, D. R., SOSOREV, A. Y., TRETIAK, S. & ZHUGAYEVYCH, A. 2019. Ground-State Geometry and Vibrations of Polyphenylenevinylene Oligomers. *The Journal of Physical Chemistry Letters,* 10**,** 3232-3239.

VASILCHENKO, V., LEVCHENKO, S., PEREBEINOS, V. & ZHUGAYEVYCH, A. 2021. Small Polarons in Two-Dimensional Pnictogens: A First-Principles Study. *The Journal of Physical Chemistry Letters,* 12**,** 4674-4680.

YANAI, T., TEW, D. P. & HANDY, N. C. 2004. A new hybrid exchange–correlation functional using the Coulomb-attenuating method (CAM-B3LYP). *Chemical Physics Letters,* 393**,** 51-57.

ZHUGAYEVYCH, A., MAZALEVA, O., NAUMOV, A. & TRETIAK, S. 2018. Lowest-Energy Crystalline Polymorphs of P3HT. *The Journal of Physical Chemistry C,* 122**,** 9141-9151.
